# DNSN-1 recruits GINS for CMG helicase assembly during DNA replication initiation in *C. elegans*

**DOI:** 10.1126/science.adi4932

**Published:** 2023-08-17

**Authors:** Yisui Xia, Remi Sonneville, Michael Jenkyn-Bedford, Liqin Ji, Constance Alabert, Ye Hong, Joseph T.P. Yeeles, Karim P.M. Labib

**Affiliations:** 1 The MRC Protein Phosphorylation and Ubiquitylation Unit, School of Life Sciences, University of Dundee, Dundee, U.K; 2 MRC Laboratory of Molecular Biology, Francis Crick Avenue, Cambridge, U.K; 3 Shandong Provincial Key Laboratory of Animal Cell and Developmental Biology, School of Life Sciences, Shandong University, Qingdao, China; 4 Division of Molecular, Cell & Developmental Biology, School of Life Sciences, University of Dundee, Dundee, U.K

## Abstract

Assembly of the CDC45-MCM-GINS helicase is the key regulated step during eukaryotic DNA replication initiation. Until now, it was unclear whether metazoa require additional factors not present in yeast. Here we show that *Caenorhabditis elegans* DNSN-1, the orthologue of human DONSON, functions during helicase assembly in a complex with MUS-101/TOPBP1. DNSN-1 is required to recruit the GINS complex to chromatin, and a cryo-electron microscopy structure indicates that DNSN-1 positions GINS on the MCM-2-7 helicase motor, by direct binding of DNSN-1 to GINS and MCM-3, using interfaces that we show are important for initiation and essential for viability. These findings identify DNSN-1 as a missing link in our understanding of DNA replication initiation, suggesting that initiation defects underlie the human disease syndrome resulting from DONSON mutations.

The initiation of chromosome replication is highly regulated in eukaryotic cells, to ensure that a single copy of each chromosome is produced in every cell cycle (1, 2). Initiation is frequently mis-regulated in human cancer and represents an interesting target for new anti-cancer therapeutics (3, 4). The key regulated step during initiation is the assembly at multiple replication origins of the DNA helicase known as CMG (CDC45-MCM2-7-GINS), around which the replisome assembles (5–7). CMG unwinds the parental DNA duplex at a replication fork and associates stably with the DNA template until neighboring forks converge during DNA replication termination. CMG is then ubiquitylated and disassembled, by the CDC-48/p97 ATPase with its ubiquitin receptors UFD-1 and NPL-4 (1, 8, 9).

The mechanism by which eukaryotic DNA replication initiates is best characterized in the budding yeast *Saccharomyces cerevisiae* (Figure S1), initially based on cellular studies and more recently through reconstituted CMG assembly reactions with purified budding yeast proteins (7, 10–12). These studies showed that the motor of the CMG helicase comprises a hetero-hexameric ring of the Mcm2-7 ATPases. Two such rings are loaded in a concerted fashion around double-stranded DNA at replication origins during G1-phase (Figure S1, step 1), thereby forming a head-to-head double hexamer of Mcm2-7 that lacks helicase activity (13, 14). Upon entry into S-phase, Cdc7 kinase (also known as Dbf4-dependent kinase or DDK) phosphorylates Mcm2-7 double hexamers (Figure S1, step 2) to create binding sites for Sld3 that recruits Cdc45 (7, 12, 15–17). Meanwhile, CDK phosphorylates both Sld3 and Sld2 (Figure S1, step 3) at sites that are recognized by pairs of BRCT domains in the amino-terminus (binds phospho-Sld3) and carboxyl-terminus (binds phospho-Sld2) of Dpb11 (18, 19). In this way, Dpb11 bridges phospho-Sld3 and Cdc45 on Mcm2-7 double hexamers to phospho-Sld2 that associates with GINS and DNA polymerase ε (20, 21), leading to the formation of a pre-initiation complex (Figure S1, step 4). Recruitment of Cdc45 and GINS triggers the assembly of two CMG helicase complexes (Figure S1, step 5), with their Mcm2-7 rings still encircling double-stranded DNA (22–24). Subsequently, the Mcm10 protein is required for a poorly understood step in which the Mcm2-7 ring is opened transiently to exclude one DNA strand (22, 23), leading to full activation of the helicase (Figure S1, step 6).

Until now, the molecular details of CMG assembly and activation have not been elucidated, or the reactions reconstituted with purified proteins, for species other than budding yeast. A functional human replisome can be assembled in vitro, if the initiation step is bypassed by mixing purified human CMG with replication fork DNA and purified orthologues of yeast replisome components (25). However, there is evidence for diversification and additional complexity amongst metazoan initiation factors, compared to the predictions of the budding yeast model. CDC7 kinase was found to be dispensable in untransformed mammalian cells due to redundancy with CDK1-Cyclin B (26). Moreover, the RECQL4 helicase has an amino terminal region with homology to yeast Sld2 and is important for replication in *Drosophila* (27), *Xenopus laevis* egg extracts (28) and chicken DT40 cells (29), yet studies of *Xenopus* RECQL4 indicate a role after CMG assembly rather than before (28, 30). Finally, the metazoan orthologue of yeast Dpb11, known variously as TOPBP1 or MUS101, contains more BRCT domains than Dpb11 (31), and a study of *Xenopus* TOPBP1 indicated that BRCTs 4-8 are dispensable for replication initiation (32), despite containing the presumed binding site for RECQL4. In contrast, BRCT3 of *Xenopus* TOPBP1 was found to be essential for replication initiation, in addition to BRCT1-2 that bind to CDK-phosphorylated TRESLIN, the orthologue of yeast Sld3 (32). However, a partner for BRCT3 of TOPBP1/MUS101 was not identified in previous studies. These findings suggested that additional initiation factors might remain to be identified in metazoan cells, and potentially also in other eukaryotes. Here we show that the *Caenorhabditis elegans* protein DNSN-1 is essential for replication initiation and acts together with MUS-101 during CMG helicase assembly. These findings provide a model for understanding the human orthologue of DNSN-1, known as DONSON (downstream neighbor of SON), which was previously identified as a genome stability factor that is mutated in microcephalic primordial dwarfism (33, 34).

## Results

### DNSN-1 binds MUS-101 in *C. elegans* early embryos and co-purifies with the CMG helicase

**Figure 1:**
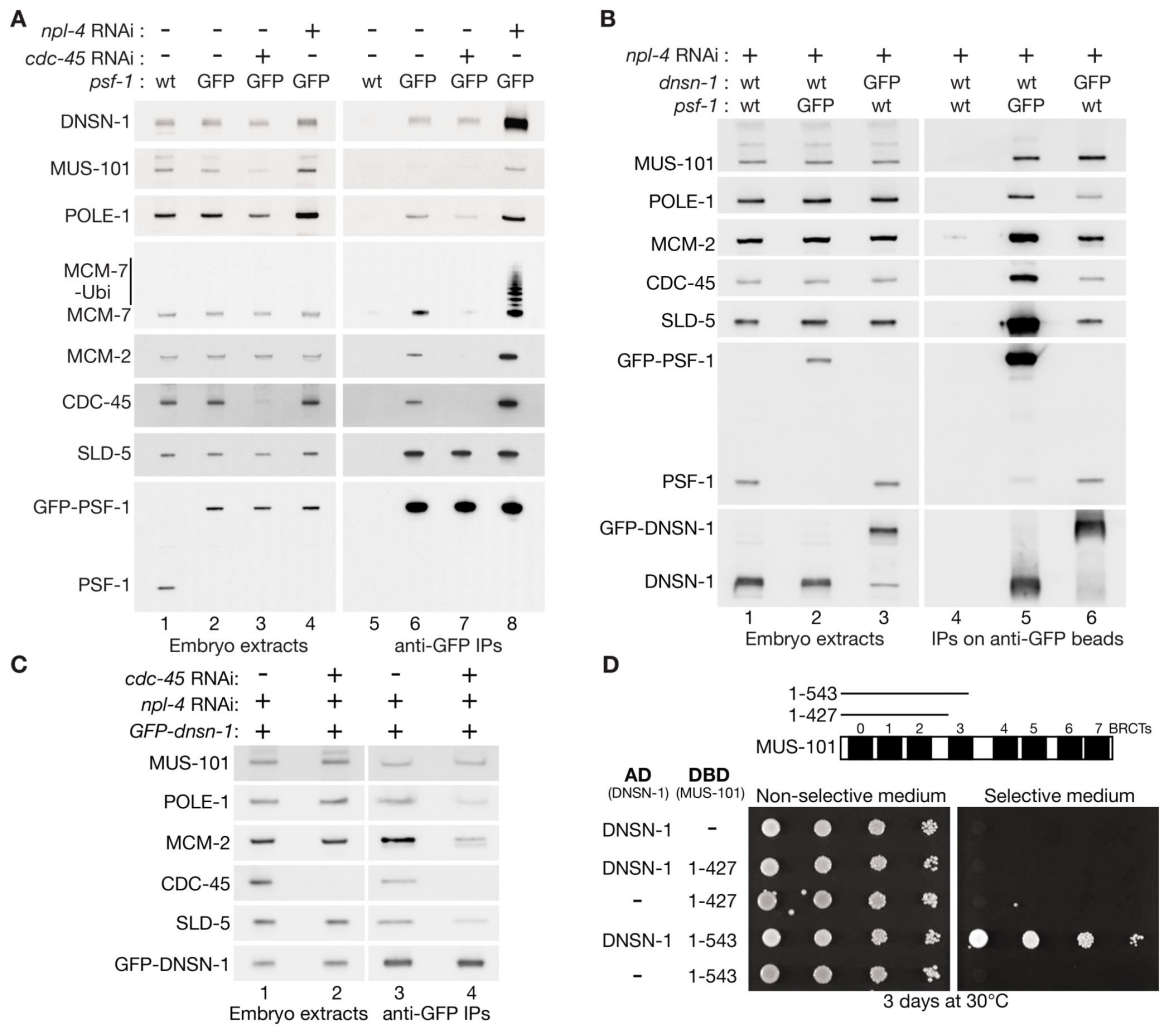
DNSN-1 associates with the *C. elegans* replisome and with MUS-101. (**A**) Control (N2) and *GFP-psf-1* (KAL1) worms were fed on bacteria expressing the indicated RNAi treatments before preparation of embryonic cell extracts and immunoprecipitation on beads coated with anti-GFP antibodies. The indicated factors were detected by immunoblotting. (**B**) Embryo extracts from the indicated strains (N2, KAL1 and KAL213) were processed as above in (A). (**C**) Worms expressing GFP-DNSN-1 (KAL213) were grown on bacteria expressing the indicated RNAi and then processed as described above in (A). (**D**) Interaction between *C. elegans* DNSN-1 and the indicated fragments of MUS-101 was monitored via the yeast two-hybrid assay. Yeast cells expressing fusions to the Gal4 Activation Domain (AD) or DNA-binding domain (DBD) were grown on ‘non-selective medium’ or ‘selective medium’, as described in Materials and Methods.

To screen for regulators of the CMG helicase in the *C. elegans* embryo, which is a rich source of replisomes, we used worms in which the PSF-1 subunit of GINS was tagged with GFP (35) to isolate GINS and sub-stoichiometric partners on anti-GFP beads (Figure S2A-B), including the CMG helicase and associated factors (Figure 1A). Extracts of control embryos were compared with extracts of *GFP-psf-1* embryos from worms that were either untreated or exposed to *cdc-45* RNAi to block replisome assembly, or treated with *npl-4* RNAi to block CMG disassembly and cause accumulation of post-termination CMG with poly-ubiquitylated MCM-7 subunit (Figure 1A lane 8), without affecting the level of GINS (35).

Mass spectrometry analysis of anti-GFP pulldowns (Table S1A and Data S1), validated subsequently by immunoblotting (Figure 1A-B), confirmed the presence of all 11 CMG subunits in GFP-PSF-1 samples, together with a set of replisome components that were previously shown to associate with the CMG helicase in *C. elegans* embryo extracts (35). DNSN-1 also co-purified with GFP-PSF-1 (Figure 1A, lane 6), in a manner that was enhanced when *npl-4* RNAi was used (Figure 1A, lane 8). This suggested that DNSN-1 might interact not only with GINS but also with other components or partners of the CMG helicase. The specificity of these interactions was confirmed via analogous pull-downs of GFP-tagged DNSN-1 from embryo extracts, analyzed by immunoblotting (Figure 1B, Figure S2C) and mass spectrometry (Figure S2D, Table S2, and Data S2).

The initiation factor MUS-101 was also enriched in pulldowns of GFP-PSF-1 from embryos treated with *npl-4* RNAi (Figure 1A, Table S1), indicating that post-termination CMG complexes were a useful tool for identifying additional replisome-binding proteins. Moreover, MUS-101 was enriched in pulldowns of GFP-tagged DNSN-1 (Figure 1B, Table S2), even after *cdc-45* RNAi treatment that impaired the interaction of DNSN-1 with CMG components (Figure 1C). These data suggested that DNSN-1 was a partner of MUS-101. A yeast two-hybrid assay indicated a direct interaction that was dependent upon BRCT3 of MUS-101 (Figure 1D), consistent with predictions that we made using AlphaFold Multimer (36) as described below.

### DNSN-1 is essential for the initiation of DNA replication

*C. elegans* DNSN-1 had not previously been linked to chromosome replication and RNAi depletion was reported to cause behavioral phenotypes linked to neuromuscular defects (37). However, mutation of the *Drosophila* orthologue, known as *humpty dumpty*, produced DNA synthesis defects (38). Moreover, mutations in human DONSON cause microcephalic dwarfism and associated DNA replication defects and DNA damage (33, 34). In addition, human DONSON co-purified with several replisome factors including subunits of the CMG helicase (34, 39).

We deleted 87% of the *dnsn-1* coding region by CRISPR-Cas9 (Figure S3A) and found that homozygous *dnsn-1Δ* was lethal during larval development (Figure S3A). Similarly, we deleted almost the entire coding region of *tres-1* (Figure S3B; worm *TRESLIN), mus-101* (Figure S3C) and *sld-2* (Figure S3D) and found that worms homozygous for the deleted alleles were sterile (Figure S3), indicating a cell proliferation defect that was consistent with previous data showing that injection of RNAi specific to *sld-2* and *tres-1/sld-3* caused embryonic lethality (40, 41).

**Figure 2:**
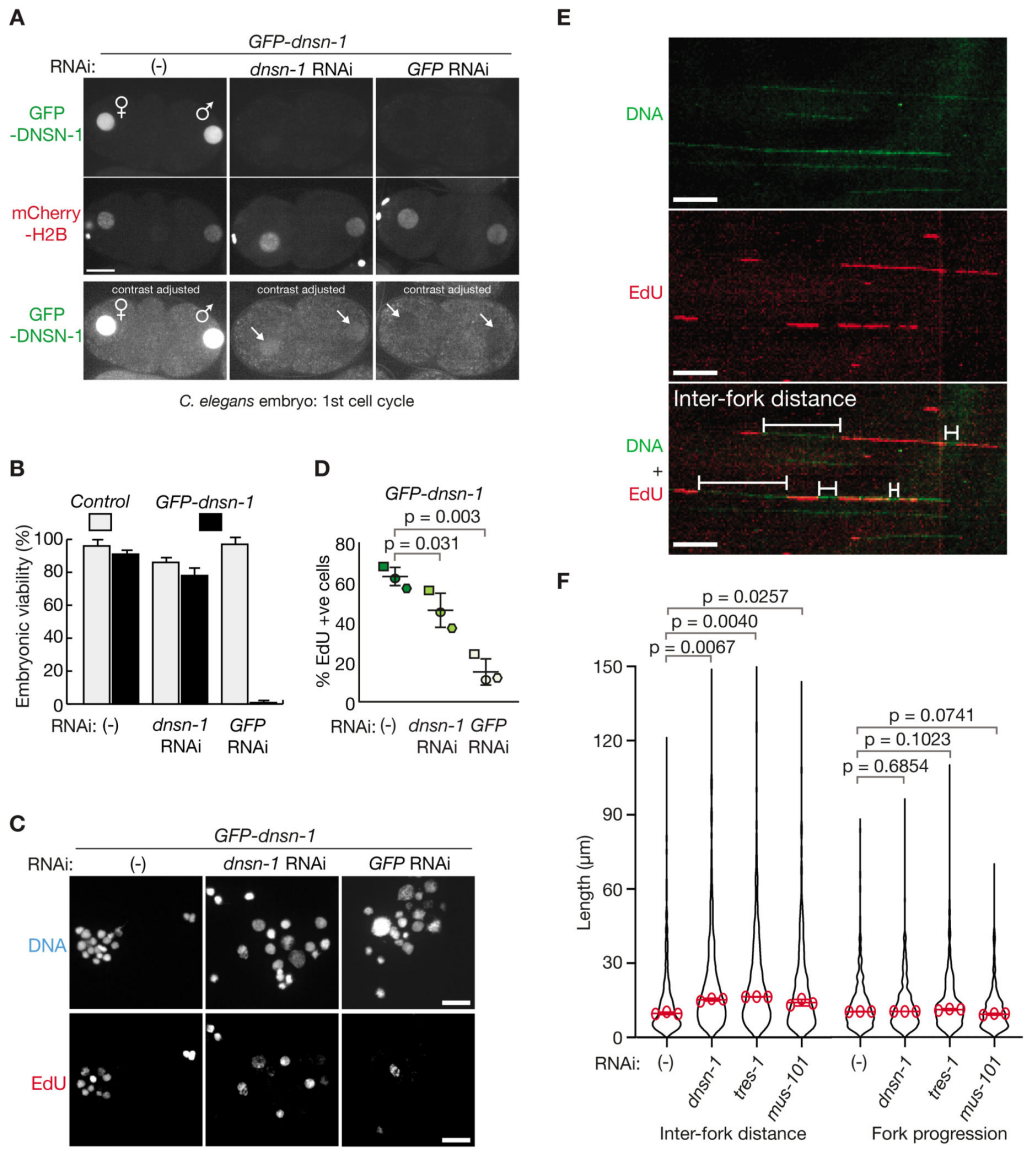
DNSN-1 is important for the initiation of DNA replication. (**A**) Worms expressing GFP-DNSN-1 and mCherry-Histone H2B (KAL267) were fed bacteria expressing the indicated RNAi and embryos were then examined by spinning disk confocal microscopy. The images show late S-phase of the first embryonic cell cycle. In the lower panels, contrast was adjusted to monitor residual GFP-DNSN-1 (arrows). Scalebars correspond to 10μm. (**B**) Control (N2) and *GFP-dnsn-1* (KAL213) worms were fed on bacteria expressing the indicated RNAi, before measurement of embryonic viability (see Materials and Methods). The data represent the means and standard deviations from three biological replicates. (**C**) *GFP-dnsn-1* worms (KAL213) were fed on bacteria expressing the indicated RNAi, before a population of single cells was isolated from embryos as described in Materials and Methods. The cells were incubated with ethynyl deoxyuridine (EdU) for 20 minutes at room temperature and then fixed before EdU detection and DNA staining with Hoechst 33342. Scale bars correspond to 10 *μ*m. (**D**) Quantification of the data in (C), corresponding to the mean values and standard deviations from three biological replicates. The samples were compared by a Kruskal–Wallis test followed by Dunn’s test, yielding the indicated p values. (**E**) Control worms (N2) were treated with a pulse of EdU and processed for molecular combing of DNA fibers, as described in Materials and Methods. DNA fibers were stained with YOYO-1 and EdU labelling was detected as above. Fork progression was defined as the length of EdU labelled tracks, whereas inter-fork distance corresponded to the distance between two EdU labelled tracks on the same fiber. The scalebar corresponds to 20 *μ*m. (**F**) Control worms (N2) were treated with the indicated RNAi and then processed as for (E). The data points from three independent experiments are depicted in a violin plot, with median values shown as red circles. The average of the median values is shown as a red bar with the associated standard deviation. The median values for each experiment were then compared by a Kruskal–Wallis test followed by Dunn’s test, yielding the p values.

Worms fed on bacteria that expressed long double-stranded RNA corresponding to *dnsn-1*, *tres-1*, *mus-101* or *sld-2* remained viable (see Figure 5A below and (40–42)), consistent with previous data from large-scale RNAi feeding screens in *C. elegans* (37, 43). This likely reflects residual protein that can still provide some level of function, despite efficient depletion, and we found that a small amount of DNSN-1 protein remained in the nucleus (Figure 2A) when *GFP-dnsn-1* worms were fed on bacteria expressing *dnsn-1* RNAi. In contrast, RNAi specific to the GFP tag caused further depletion of nuclear GFP-DNSN-1 (Figure 2A) and led to a total loss of viability in *GFP-dnsn-1* worms, without affecting control animals (Figure 2B). Together with the lethal phenotype of *dnsn-1Δ*, these findings demonstrated that DNSN-1 is essential for viability in *C. elegans* and indicated that *dnsn-1* RNAi and GFP RNAi could be used to achieve milder and stronger depletion respectively of GFP-tagged DNSN-1 for phenotypic analysis.

To examine the role of DNSN-1 during DNA replication in the *C. elegans* embryo, we exposed embryonic cells from *GFP-dnsn-1* embryos to a pulse of the nucleoside precursor EdU (5-ethynyl-2’-deoxyuridine). Incorporation of EdU into genomic DNA was impaired by *dnsn-1* RNAi and strongly inhibited by GFP RNAi (Figure 2C-D), indicating that DNSN-1 is essential for chromosome replication. To determine at which stage DNSN-1 acts, we exposed embryonic cells to a pulse of EdU as above, after partial depletion of DNSN-1, TRES-1 or MUS-101 by RNAi, and then used DNA combing (44), to monitor origin firing and the progression of DNA replication forks (Figure 2E). Whereas fork progression was not significantly affected in worms treated with RNAi to *dnsn-1*, *tres-1* or *mus-101*, the spacing between replication forks increased in all cases (Figure 2F), corresponding to a defect in origin firing. These findings indicate that DNSN-1 is dispensable for fork progression but is required for the initiation of DNA replication in *C. elegans*, together with TRES-1 and MUS-101 (Figure 2F) and SLD-2 (40).

### MUS-101-dependent association of DNSN-1 with pre-initiation complexes

**Figure 3:**
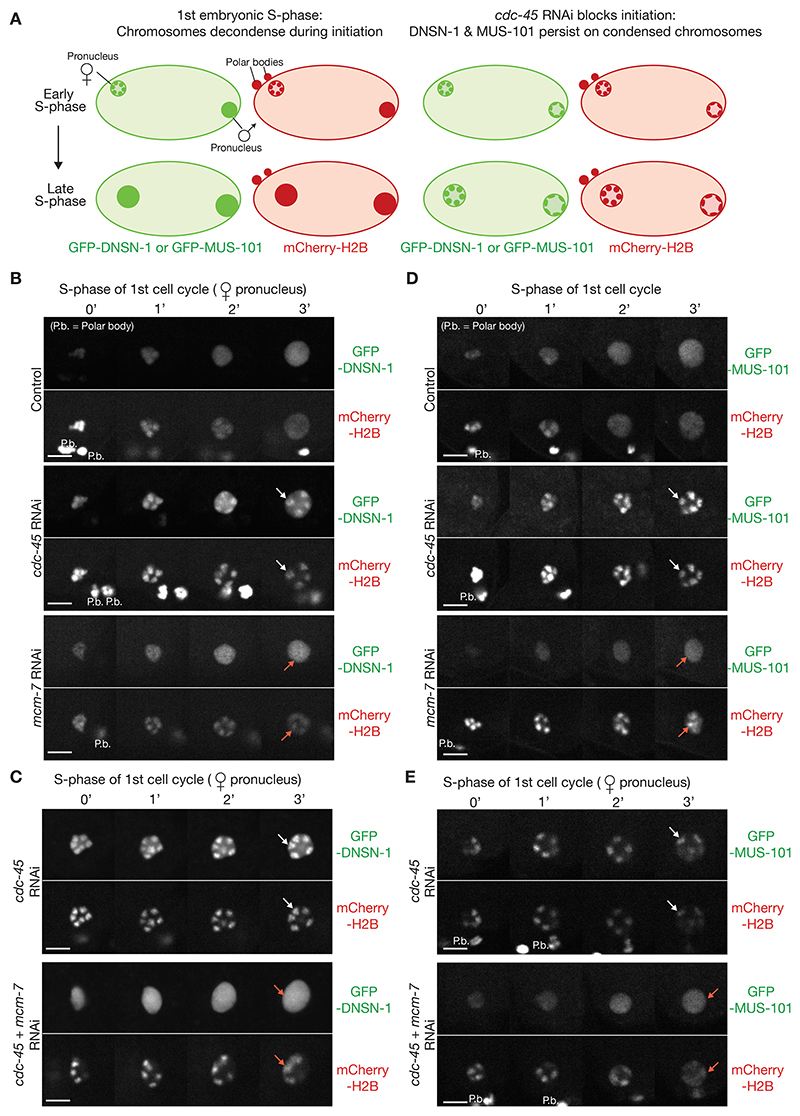
DNSN-1 and MUS-101 are recruited to pre-initiation complexes on chromatin during early S-phase. (**A**) Illustration of how DNSN-1 and MUS-101 associate with condensed chromatin during early S-phase of the first embryonic cell cycle (left) and remain on chromatin when decondensation is delayed in worms lacking CDC-45 (right). (**B**) Embryos expressing GFP-DNSN-1 and mCherry-Histone H2B (KAL267) were treated with the indicated RNAi, before analysis by time-lapse video microscopy. The images show progression of the female pronucleus through early S-phase of the first embryonic cell cycle. White arrows indicate DNSN-1 on persistently condensed chromosomes in the absence of CDC-45. Red arrows indicate loss of DNSN-1 from condensed chromatin in the absence of MCM-7. (**C**) Similar experiment comparing embryos treated with *cdc-45* RNAi or *cdc-45* + *mcm-7* double RNAi (white arrows show DNSN-1 on condensed chromosomes, red arrows indicate loss of DNSN-1 from condensed chromosomes without MCM-7). (**D**-**E**) Equivalent experiments to those in (B) and (C) but with embryos expressing GFP-MUS-101 and mCherry-Histone H2B (KAL276). Scalebars correspond to 5μm. Note that the female pronucleus is located at a variable depth within the embryo, which can lead to differences in brightness between images.

The *C. elegans* early embryo divides rapidly and DNA replication initiates on condensed chromatin in the first cell cycle. CDC-45 is detected on the six condensed chromosomes at the end of the second meiotic division, dependent upon MCM-2-7 proteins (45). Previous work suggests that the CMG helicase is required for rapid chromosome decondensation in the *C. elegans* early embryo (45). Although chromosome decondensation proceeds normally when DNA synthesis is inhibited downstream of CMG assembly by RNAi depletion of the single-strand DNA binding protein RPA, the ribonucleotide reductase RNR-1, or the polymerase accessory factor PCN-1 (PCNA), RNAi depletion of *cdc-45* causes a profound delay in chromosome decondensation upon entry into S-phase of the first embryonic cell cycle (45). These findings suggest that the DNA unwinding activity of the CMG helicase at replication forks drives rapid chromosome decondensation in the early embryonic cell cycles (Figure 3A).

When cells entered S-phase in the absence of CDC-45, both DNSN-1 (Figure 3A-C, *cdc-45* RNAi) and MUS-101 (Figure 3D-E, *cdc-45* RNAi) were detected on the chromosomes that remained condensed during early S-phase. In contrast, depletion of MCM-2-7 (Figure 3B+D, *mcm-7* RNAi), or co-depletion of CDC-45 and MCM-2-7 (Figure 3C+E, *cdc-45 + mcm-7* RNAi), delayed chromosome decondensation without detectable chromatin association of DNSN-1 or MUS-101. These data indicate that DNSN-1 and MUS-101 associate with pre-initiation complexes containing the loaded MCM-2-7 ATPases, which persist on condensed chromosomes when CMG assembly is blocked. Consistent with this view, DNSN-1 was also observed on condensed chromosomes during early S-phase after depletion of PSF-1 (Figure S4, *psf-1* RNAi). However, DNSN-1 was not detected on condensed chromosomes after depletion of MUS-101 (Figure S4, *mus-101* RNAi). Since MUS-101 is a partner of DNSN-1 (Figure 1), this indicates that MUS-101 helps to recruit DNSN-1 to the pre-initiation complexes that assemble on MCM-2-7 double hexamers before CMG helicase assembly.

### DNSN-1 is required for chromatin recruitment of GINS but not CDC-45 during CMG assembly

**Figure 4:**
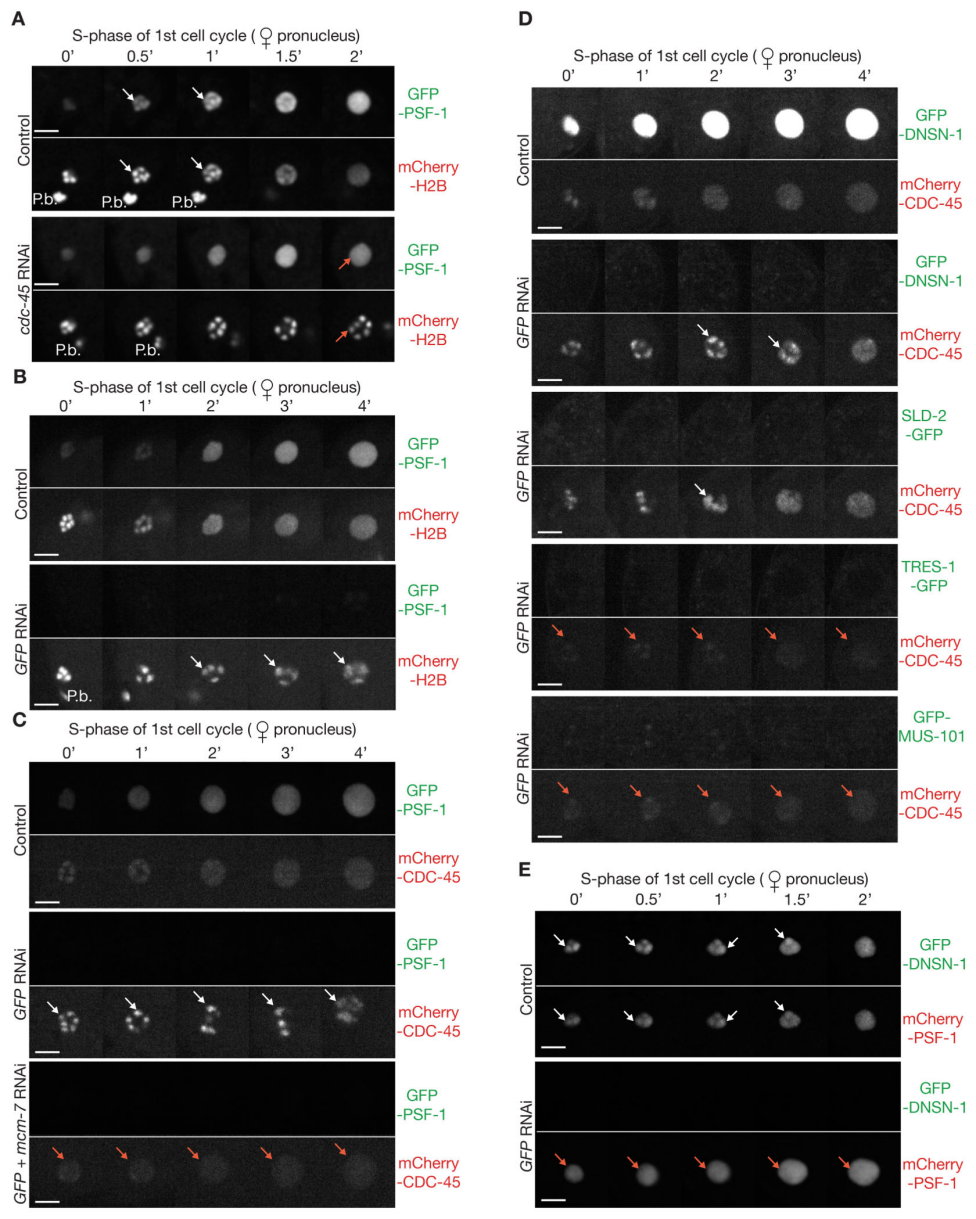
DNSN-1 and SLD-2 are required for chromatin recruitment of GINS but not CDC-45 during early S-phase. (**A**) Embryos expressing GFP-PSF-1 and mCherry-Histone H2B (KAL3) were grown with or without *cdc-45* RNAi and then analyzed by time-lapse video microscopy. White arrows denote GINS on condensed chromosomes in early S-phase (63% embryos, n = 16); red arrows show loss of GINS from condensed chromosomes in absence of CDC-45 (100% embryos, n = 12). (**B**) Similar experiment +/- GFP RNAi. White arrows indicate the persistence of condensed chromosomes in the absence of GFP-PSF-1. (**C**) Embryos expressing GFP-PSF-1 and mCherry-CDC-45 (KAL266) were exposed to the indicated RNAi treatment and analyzed as above. White arrows indicate CDC-45 on chromatin in the absence of PSF-1. Red arrows denote loss of CDC-45 from chromatin in combined absence of PSF-1 + MCM-7. (**D**) The indicated strains (KAL268, KAL271, KAL274 and KAL277) were grown as shown in the presence or absence of *GFP* RNAi, before analysis as above. White arrows indicate persistence of CDC-45 on chromatin in the absence of DNSN-1 or SLD-2, compared to control embryos. Red arrows show loss of CDC-45 from chromatin in absence of TRES-1 or MUS-101. (**E**) Embryos expressing GFP-DNSN-1 and mCherry-PSF-1 (KAL269) were processed as above. White arrows denote co-localization on chromatin of mCherry-PSF-1 and GFP-DNSN-1 during early S-phase in control embryos. Red arrows indicate loss of GFP-PSF-1 from chromatin in absence of DNSN-1. Scalebars correspond to 5μm. Note that the female pronucleus is located at a variable depth within the embryo, which can lead to differences in brightness between images.

Upon entry into S-phase of the first embryonic cell cycle, GFP-PSF-1 was observed on condensed chromosomes (Figure 4A, Control), but this was lost upon depletion of CDC-45 (Figure 4A, *cdc-45* RNAi), indicating that chromatin-bound PSF-1 corresponds to assembled CMG helicase complexes. Depletion of GFP-PSF-1 delayed chromosome decondensation upon entry into the first embryonic S-phase as expected (Figure 4B, *GFP* RNAi). However, CDC-45 was still detected on condensed chromosomes in the absence of PSF-1 under such conditions (Figure 4C, *GFP* RNAi) and this was dependent upon the MCM-2-7 complex (Figure 4C, *GFP* + *mcm-7* RNAi). This indicated that the initial recruitment of CDC-45 to chromatin-loaded MCM-2-7 in early S-phase does not require GINS and can be monitored independently. Therefore, the *C. elegans* early embryo provides a useful experimental system to distinguish which metazoan initiation factors are required for recruitment of CDC-45 or GINS to MCM-2-7 double hexamers during DNA replication initiation.

Consistent with its role during initiation (Figure 2), and its presence in pre-initiation complexes with MUS-101 (Figure 3, Figure S4), depletion of DNSN-1 delayed chromosome decondensation during early S-phase (Figure S5) suggesting that DNSN-1 is required for some aspect of CMG helicase assembly. However, mCherry-CDC-45 was still recruited to condensed chromosomes during early S-phase in embryos lacking GFP-DNSN-1 (Figure 4D, 100% embryos, n = 5), analogous to the effect of depleting GFP-PSF-1. The same was true in *GFP-sld-2* embryos treated with *GFP* RNAi, whereas depletion of GFP-TRES-1 or GFP-MUS-101 impaired the recruitment of mCherry-CDC-45 to chromatin (Figure 4D). Similarly, RNAi depletion of DNSN-1, SLD-2, TRES-1 or MUS-101 delayed chromosome decondensation upon entry into S-phase of the second cell cycle, in worms expressing GFP-histone H2B and mCherry-CDC-45, but CDC-45 persisted on condensed chromatin in response to *dnsn-1* or *sld-2* RNAi, whereas depletion of TRES-1 or MUS-101 impaired chromatin association of CDC-45 (Figure S6). Overall, these findings indicated that DNSN-1 and SLD-2 are dispensable for CDC-45 chromatin recruitment in early S-phase, in contrast to both TRES-1 and MUS-101.

mCherry-PSF-1 co-localized with GFP-DNSN-1 on condensed chromatin during early S-phase (Figure 4E and Figure S7, Control, 80% embryos, n = 10). In contrast, chromatin association of mCherry-PSF-1 was lost upon depletion of DNSN-1 (Figure 4E and Figure S7, *GFP* RNAi, 100% embryos, n = 14), indicating that DNSN-1 is required for stable incorporation of GINS into CMG helicase complexes on chromatin during S-phase. To ensure that the failure to detect GINS on chromatin in the absence of DNSN-1 was not due to premature ubiquitylation and disassembly of the CMG helicase, we depleted DNSN-1 in combination with NPL-4 (Figure S8). Consistent with previous observations (35), NPL-4 depletion led to persistence of mCherry-PSF-1 on chromatin during mitosis (Figure S8A-B, *npl-4* RNAi), reflecting a failure of CMG disassembly during DNA replication termination. However, chromatin association of mCherry-PSF-1 was reduced around five-fold when GFP-DNSN-1 was depleted in addition to NPL-4 (Figure S8A-C, *npl-4* + GFP RNAi), similar to co-depletion of NPL-4 and TRES-1 (Figure S8A-C, *npl-4* + *tres-1* RNAi), NPL-4 and MUS-101 (Figure S8D-F, *npl-4* + *mus-101* RNAi), or NPL-4 and SLD-2 (Figure S8D-F, *npl-4* + GFP RNAi). In contrast, chromatin-bound GFP-PSF-1 was not reduced by co-depletion of RPA-1 and NPL-4 (46), which blocked DNA replication after CMG assembly. These data indicated that DNSN-1 is required to assemble the CMG helicase, together with MUS-101, TRES-1 and SLD-2. Together with the data for recruitment of PSF-1 and CDC-45 during early S-phase (Figure 4), these findings also indicate that DNSN-1 is specifically required for chromatin recruitment of GINS but is dispensable for chromatin recruitment of CDC-45.

### Depletion of DNSN-1 or MUS-101 is synthetic lethal with *mcm-10Δ* or *cdc-7Δ*

**Figure 5:**
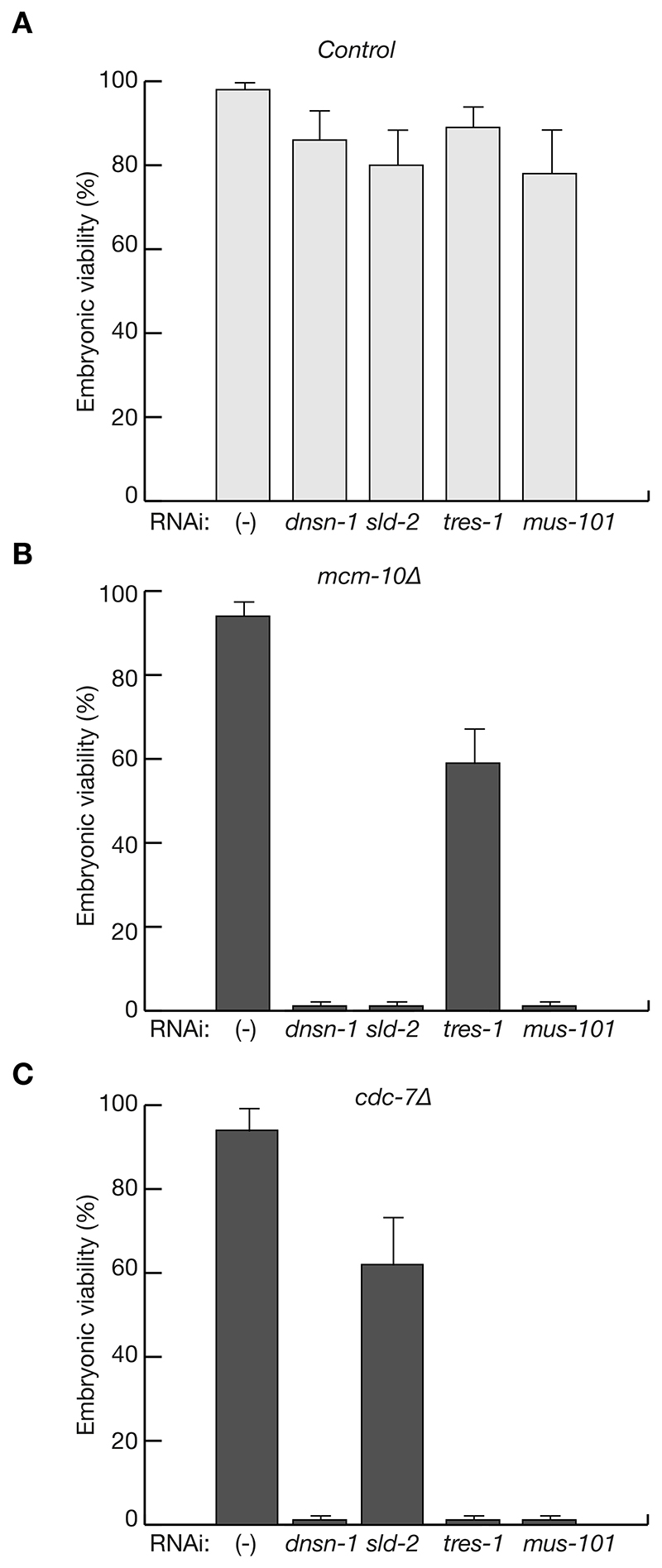
Depletion of DNSN-1 or other initiation factors causes synthetic lethality in embryos lacking MCM-10 or CDC-7. (**A**) Embryonic viability of control worms (N2) was analyzed after feeding on bacteria expressing the indicated RNAi, or empty vector as control. The data represent the mean values and standard deviations from three biological replicates. (**B**) Similar analysis of *mcm-10Δ* worms (KAL259). (**C**) Equivalent experiment with *cdc-7Δ* worms (KAL255).

As noted above, budding yeast Mcm10 is essential for activation of the newly assembled CMG helicase (22, 47, 48). However, deleting 95% of the coding sequence of *C. elegans mcm-10* (Figure S9A, D and E) was not lethal (Figure 5B), indicating that other initiation factors must support CMG activation in the absence of MCM-10. To explore how other initiation factors might compensate for the loss of MCM-10, we tested the effects of depleting such factors by RNAi in *mcm-10Δ* worms, taking advantage of the fact that RNAi depletion of DNSN-1, SLD-2, or TRES-1 is incomplete as discussed above, and so is viable as a single treatment (Figure 5A). Depletion of DNSN-1, MUS-101 or SLD-2 led to a loss of viability in combination with *mcm-10Δ*, whereas *tres-1* RNAi only caused reduced viability in the absence of MCM-10 (Figure 5A-B).

Previous work showed that worms lacking 68% of the coding sequence of *cdc-7* are viable (49), and we found that the same is true for worms in which 97% of the coding sequence was removed by CRISPR-Cas9 (Figure S9B + D; Figure 5C). These findings demonstrate that the CDC-7 kinase is not essential for DNA replication initiation in *C. elegans*. However, RNAi depletion of DNSN-1 or MUS-101 was lethal in *cdc-7Δ* worms (Figure 5C), similar to the effects of depleting DNSN-1 or MUS-101 in *mcm-10Δ*. RNAi depletion of TRES-1 was also lethal in *cdc-7Δ* worms, whereas depletion of SLD-2 reduced viability (Figure 5C). In addition, *cdc-7Δ mcm-10Δ* worms were unable to produce viable offspring (n = 7 double mutant worms).

These data are consistent with a role for MCM-10 and CDC-7 during the initiation of DNA replication in *C. elegans* but indicate some level of redundancy with other factors. Though DNSN-1, TRES-1, MUS-101 and SLD-2 are all important for CMG assembly (Figure 4 and Figure S8), the synthetic lethality data for *mcm-10Δ* suggest that DNSN-1, SLD-2 and MUS-101 might additionally contribute to the helicase activation step. These findings further indicate that the mechanism and regulation of DNA replication initiation are more complex in *C. elegans* than would have been predicted by the model derived from studies in budding yeast.

### Dimeric DNSN-1 binds the GINS and MCM-3 components of the CMG helicase

**Figure 6:**
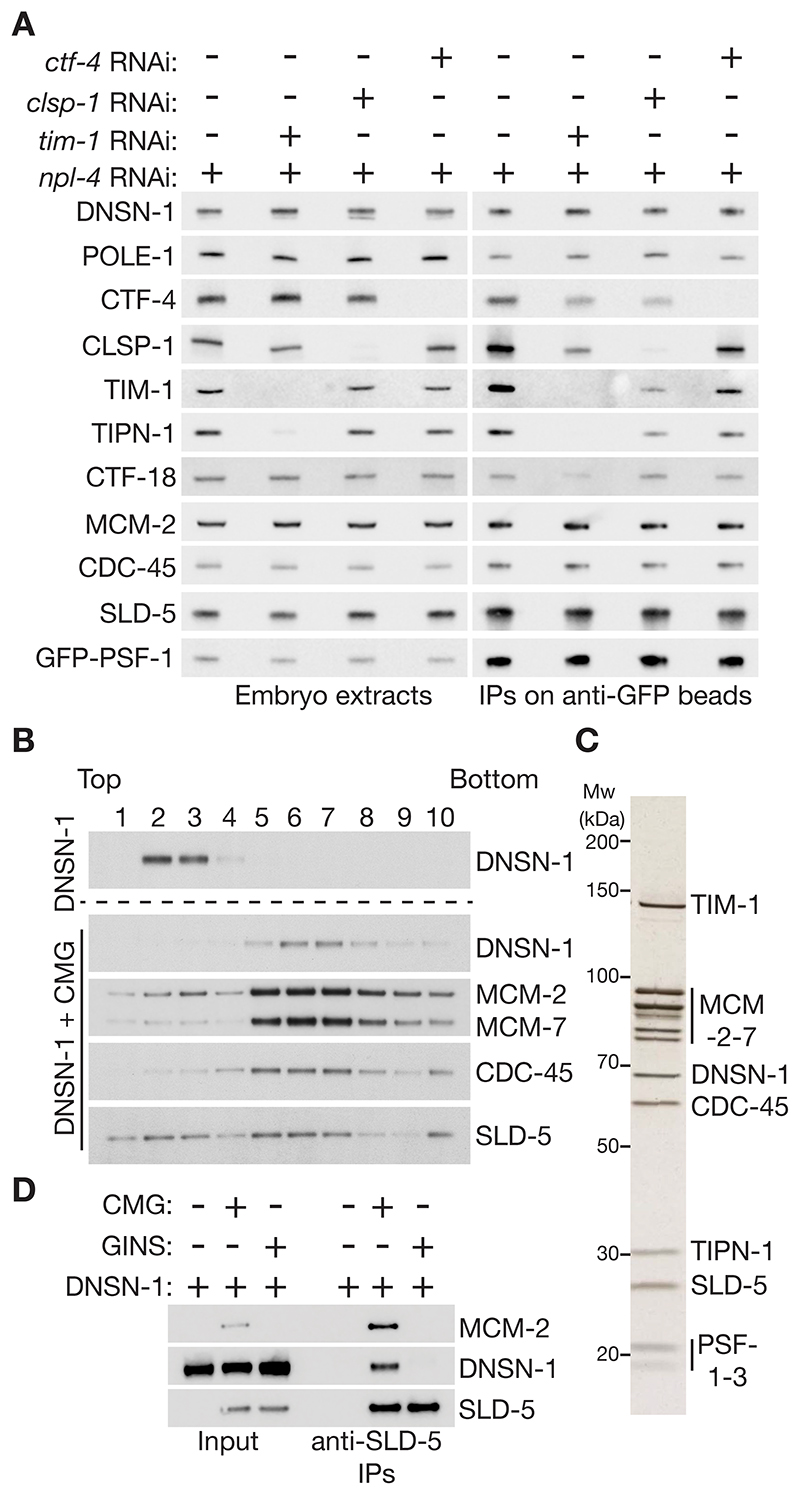
DNSN-1 binds directly to the CMG helicase. (**A**) *GFP-psf-1* worms (KAL1) were fed on bacteria expressing the indicated RNAi treatments before preparation of embryonic cell extracts and isolation of GFP-PSF-1 by immunoprecipitation on anti-GFP beads. The indicated proteins were monitored by immunoblotting. (**B**) Recombinant forms of *C. elegans* DNSN-1 (25 nM dimer) and CMG helicase (100 nM) were mixed and applied to a 10-30% glycerol gradient, in the presence of annealed oligonucleotides with 45 bp double-stranded DNA and a 3’ flap of 39nt single-stranded DNA (see Table S5). Migration of DNSN-1 and CMG was monitored via immunoblotting of gradient fractions. (**C**) In a similar experiment to that in (B), the peak fraction (fraction 8 from Figure S11A) containing the complex of DNSN-1 and CMG was resolved by SDS-PAGE and analyzed by silver staining. (**D**) The indicated recombinant proteins were mixed and incubated before isolation of CMG or GINS via immunoprecipitation on beads coated with anti-SLD-5 antibodies. The indicated factors were monitored by immunoblotting.

As a first step towards exploring whether DNSN-1 associates directly with components of the CMG helicase, we tested whether co-purification of DNSN-1 with GFP-PSF-1 from embryo extracts required the core replisome components CTF-4, TIM-1 and CLSP-1 (CLASPIN). RNAi depletion of these factors did not affect the co-purification of DNSN-1 with PSF-1 (Figure 6A, Table S3 and Data S3), suggesting that DNSN-1 either binds directly to CMG components or to other CMG partners such as DNA polymerase ε.

To assay for direct binding of DNSN-1 to CMG, we purified recombinant forms of the *C. elegans* CMG complex and DNSN-1 from budding yeast cells and bacteria respectively. The migration of DNSN-1 in a size exclusion column indicated self-association (Figure S10A), consistent with DNSN-1 homo-dimerization predicted by AlphaFold Multimer (Figure S10B). Subsequently, the proteins were analyzed by glycerol gradient ultracentrifugation, both individually and after mixing. This showed that DNSN-1 formed a stable complex with the CMG helicase (Figure 6B-C), under conditions where association of DNSN-1 with purified GINS could not be detected (Figure 6D). Although DNSN-1 can associate with GINS in embryo extracts in the absence of CMG (Figure 1A, *cdc-45* RNAi), these data indicate that DNSN-1 binds more tightly to CMG via additional interaction(s) with MCM-2-7 or CDC-45.

**Figure 7:**
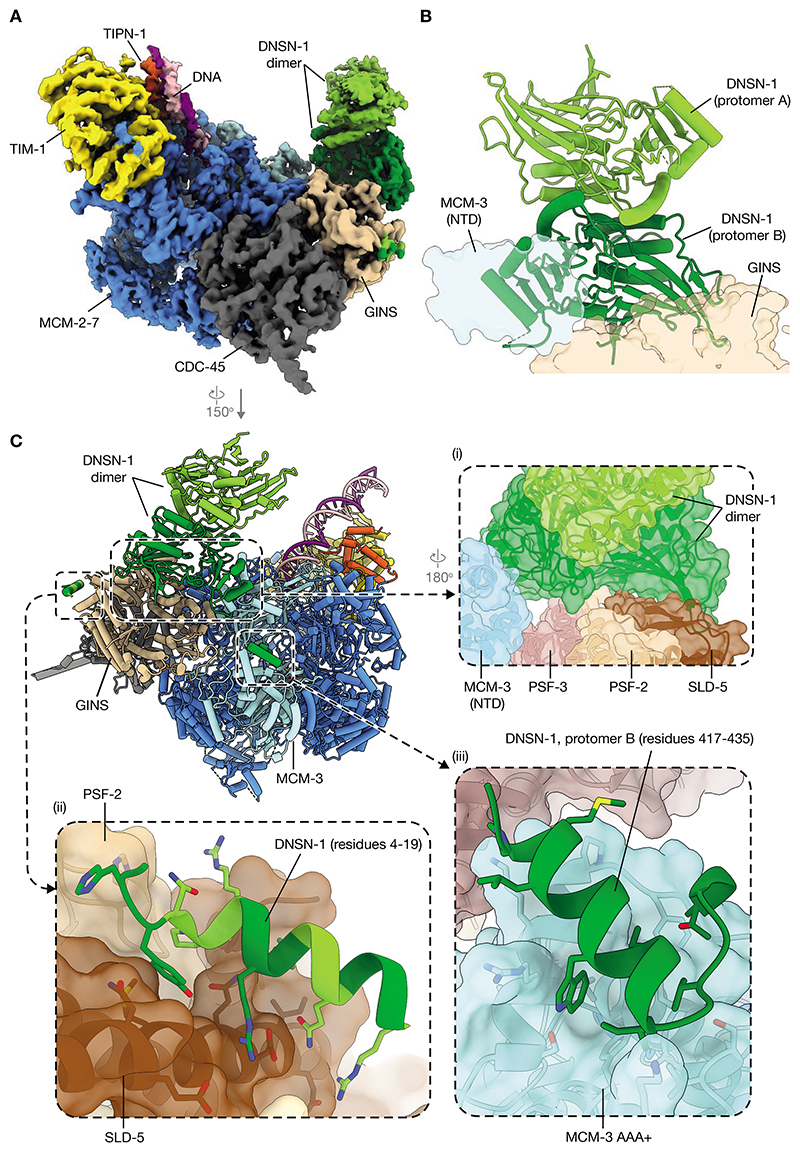
Cryo-EM analysis reveals binding of dimeric DNSN-1 to the GINS and MCM-3 components of CMG. (**A**) Cryo-EM density map colored according to subunit. (**B**) Atomic model of the DNSN-1 homodimer. Neighboring components of the CMG helicase that are bound by DNSN-1 are represented as transparent surfaces. (**C**) Full atomic model of the complex formed between DNSN-1 and the CMG helicase (associated with replisome components TIM-1 and TIPN-1, and fork DNA). The GINS (brown) and MCM-3 (light blue) components of CMG that bind DNSN-1 are indicated. Inset images display zoomed-in views of the DNSN-1 interaction sites with CMG: (i) the interface formed between one DNSN-1 protomer, the amino-terminal domain (NTD) of MCM-3, and the GINS subunits SLD-5, PSF-2 and PSF-3; (ii) the interface formed between the DNSN-1 amino-terminus and the GINS subunits SLD-5 and PSF-2; DNSN-1 is colored alternating shades of green to indicate uncertainty as to which DNSN-1 protomer this helix belongs (since it is linked to the folded domain by ~ 125 disordered residues); (iii) the interface formed between DNSN-1 and the MCM-3 AAA+ domain. For clarity, models are represented as cartoons, or cartoons within transparent surfaces.

The association of DNSN-1 with the CMG helicase was then analyzed by cryo-electron microscopy (EM), in the presence of replication fork DNA as well as a complex of TIM-1 and TIPN-1 that was included to stabilize association of CMG with DNA (50) (Figure 7, Figures S11-S14, Table S4). The resulting structure, which was determined using cryo-EM density maps with average nominal resolutions of 2.6 Å – 3.8 Å (FSC = 0.143 criterion), confirmed the existence of a DNSN-1 homodimer (Figure 7B) that associates with the GINS and MCM-3 components of the helicase. Specifically, the folded domain of one protomer of the DNSN-1 dimer forms a large interface on the amino terminal (forward-facing) side of the MCM-2-7 motor of the CMG helicase. In this way, extensive interactions with the PSF-2, PSF-3 and SLD-5 subunits of GINS serve to position the DNSN-1 dimer on CMG, together with further interactions with the MCM-3 amino terminal domain (Figure 7C (i), NTD, N terminal domain).

In addition to the folded domains of the DNSN-1 dimer, cryo-EM density corresponding to an α-helix is observed at two other positions within the complex. Firstly, one α-helix is bound to a distant region of GINS beside the N-terminus of SLD-5, where it contacts SLD-5 and PSF-2. Aided by an AlphaFold-Multimer prediction, the amino terminus of DNSN-1 (residues 4-19) can be unambiguously modelled into this density (Figure 7C (ii), Figure S13A-D). The second a-helix interacts with the AAA+ motor domain of MCM-3 and features clear density attributable to a tryptophan residue (Figure 7C (iii), Figure S13E). With the aid of AlphaFold-Multimer, this density was determined to correspond to DNSN-1 residues 417-435 (including W425), belonging to the same DNSN-1 protomer for which the folded domain interacts with GINS and the MCM3-NTD, as described above (Figure 7C (iii), Figure S13E-H). The interaction of DNSN-1 with the MCM-3 AAA+ domain and the MCM-3 NTD, in addition to multiple GINS subunits, likely explains the greater affinity of DNSN-1 for CMG compared to isolated GINS.

**Figure 8:**
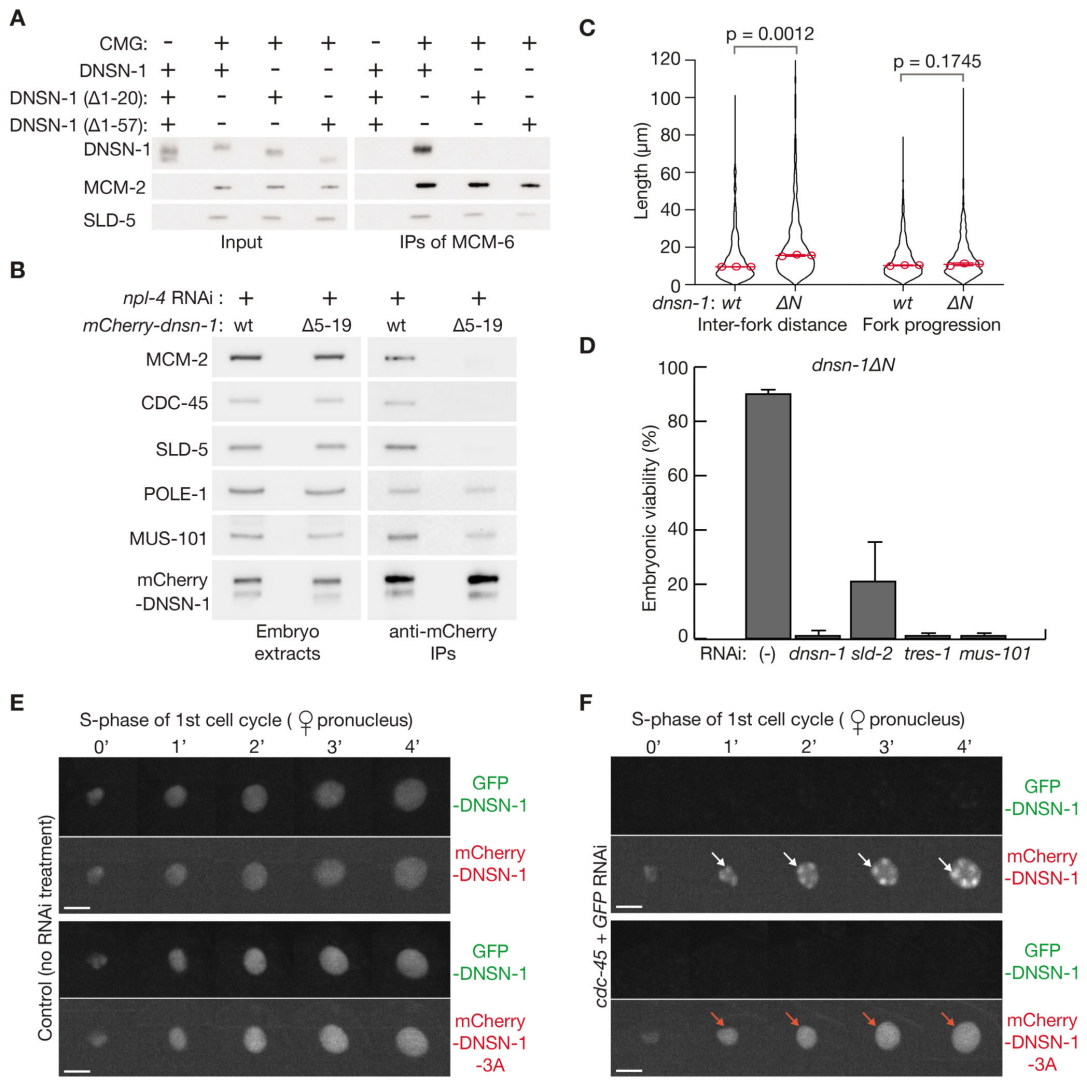
Interaction of DNSN-1 with GINS and MCM-3 is important for DNA replication initiation. (**A**) Recombinant wild type DNSN-1, or alleles with small truncations in the amino-terminal binding site for GINS, were mixed as shown with recombinant CMG and then incubated before isolation of CMG via immunoprecipitation on beads coated with anti-MCM-6 antibodies. The indicated proteins were monitored by immunoblotting. (**B**) Worms expressing mCherry-DNSN-1 (KAL256) or mCherry-DNSN-1Δ5-19 (*dnsn-1ΔN*, KAL257) from the endogenous *dnsn-1* locus were fed on bacteria expressing *npl-4* RNAi, before preparation of embryonic cell extracts and immunoprecipitation on beads coated with an antibody that recognized the mCherry tag. The indicated factors were monitored by immunoblotting. (**C**) Control worms (N2) and *dnsn-1ΔN* (KAL221) were analyzed by EdU incorporation and molecular combing of DNA fibers, as in Figure 2F above. The individual data points from three repeats are depicted in a violin plot. The median values are shown in red circles, whilst red bars and error bars represent the average of the median values and the associated standard deviations. The median values for each sample were then compared by a paired two-tailed t-test, yielding the indicated p values. (**D**) *dnsn-1ΔN* worms (KAL221) were fed on bacteria expressing the indicated RNAi, or empty vector as negative control. The data represent the means and standard deviations from three biological replicates. (**E**) Embryos expressing *GFP-dnsn-1/mCherry-dnsn-1* (progeny of KAL213 and KAL256) or *GFP-dnsn-1/mCherry-dnsn-1-3A* (progeny of KAL213 and KAL303), were analyzed during S-phase of the first cell cycle by time-lapse video microscopy. DNSN-1-3A has mutations in the binding site of DNSN-1 with the AAA+ domain of MCM-3 (see Figure 7C (iii), Figure S9F and Table S5). (**F**) Similar experiment after RNAi depletion of CDC-45 and GFP-DNSN-1. White arrows show the association of mCherry-DNSN-1 with pre-initiation complexes that persist on condensed chromosomes in the absence of CDC-45. Red arrows indicate lack of chromatin association for DNSN-1-3A. Scalebars correspond to 5*μ*m.

To explore the physiological significance of DNSN-1’s association with GINS and MCM-3, we generated worms with structure-guided mutations in DNSN-1 that were predicted either to interfere with binding to GINS, or to impair association with MCM-3. Firstly, we generated an allele that lacks amino acids 5-19 of DNSN-1 (*dnsn-1ΔN*, Figure S9C-E), within the amino terminal helix of DNSN-1 that binds near the amino-terminus of SLD-5 (Figure 7C (ii)) and is important for stable association of the DNSN-1 dimer with CMG (Figure 8A-B),

Worms homozygous for the *dnsn-1ΔN* allele are viable, but DNA combing showed that *dnsn-1ΔN* produced a defect in DNA replication initiation (Figure 8C, *dnsn-1ΔN* increased the inter-fork distance). As seen above for *cdc-7Δ* (Figure 5C), *dnsn-1ΔN* was synthetic lethal with RNAi specific to *mus-101*, *tres-1* or *dnsn-1* (Figure 8D), and *dnsn-1Δ* was also lethal in combination with both *mcm-10Δ* (homozygotes were sterile, n = 16 animals) and *cdc-7Δ* (homozygotes were sterile, n = 16 animals), likely reflecting additive defects in DNA replication initiation. These findings indicate that the association of DNSN-1 with GINS is important for initiation.

In addition, we generated the *dnsn-1-3A* allele (Figure S9F) with mutations in three conserved residues in DNSN-1 that contact the AAA+ domain of MCM-3 (Figure 7C (iii), Figure S15A). When homozygous, we found that 100% *dnsn-1-3A* worms were sterile (Figure S9F). To analyze the phenotype of *dnsn-1-3A* in the early embryo, we generated heterozygous *mCherry-dnsn-1-3A/GFP-dnsn-1* worms, together with *mCherry-dnsn-1/GFP-dnsn-1* control worms (Figure 8E), and then treated them with RNAi to *GFP* and *cdc-45*, to deplete GFP-DNSN-1 and block CMG assembly, thereby leading to the accumulation of pre-initiation complexes on condensed chromosomes, as described above (Figure 3). Whereas wild type mCherry-DNSN-1 accumulated on condensed chromosomes during S-phase under such conditions (Figure 8F, mCherry-DNSN-1), the DNSN-1-3A mutant was present in the nucleus but was not detected on condensed chromatin (Figure 8F, mCherry-DNSN-1-3A). These data indicate that the interaction of DNSN-1 with MCM-3 is essential in *C. elegans* and is required for association of DNSN-1 with pre-initiation complexes, which assemble on loaded MCM-2-7 double hexamers in early S-phase.

## Discussion

Together with past work (40, 41), our data demonstrate that CMG helicase assembly during S-phase in *C. elegans* requires TRES-1, MUS-101, SLD-2 and DNSN-1. Our data indicate that TRES-1 and MUS-101 are required to recruit CDC45 to the pre-initiation complexes that form on MCM-2-7 double hexamers (Figure 4), likely involving direct binding of the conserved Sld3/TRESLIN domain of TRES-1 (51) to CDC-45 (52). MUS-101 uses its BRCT1-2 domains to bind to CDK-phosphorylated TRES-1 (41) and recruits DNSN-1 (Figure S4), which together with SLD-2 is required to recruit GINS (Figure 4, Figure S8). Our data indicate that DNSN-1 binds simultaneously to GINS and to the MCM-3 subunit of MCM-2-7 double hexamers (Figures 7-8), thereby positioning GINS in a precise fashion to promote CMG assembly.

Cryo-EM demonstrates that DNSN-1 is a dimer (Figure 7). Almost all the observed contacts with GINS and MCM-3 can be assigned to a single DNSN-1 protomer (Figure 7), suggesting that the DNSN-1 dimer might simultaneously contribute to the production of two CMG complexes during initiation. Symmetrical engagement of both DNSN-1 protomers with MCM-3 and GINS would likely require initial disruption of the MCM-2-7 double hexamer, to allow rotation of the MCM-2-7 rings.

Orthologues of DNSN-1/DONSON are present in other metazoan species and AlphaFold-Multimer predicts that human DONSON is a dimer that interacts with the AAA+ domain of MCM3, the SLD5 subunit of GINS and the BRCT3 domain of TOPBP1/MUS101 (Figure S15). This suggests an evolutionarily conserved role for metazoan DONSON orthologues during DNA replication initiation, consistent with recent data for *Xenopus* DONSON (53, 54). DONSON orthologues are also present in plants but are absent from budding yeast and many fungi, suggesting that the role of DNSN-1/DONSON in DNA replication initiation emerged at an early step of eukaryotic evolution but was subsequently lost during fungal evolution.

The ability of DNSN-1 to bind to CMG suggests that it might also have roles after helicase assembly. One possibility is that DNSN-1 contributes to the helicase activation step that in yeast requires Mcm10, since worms lacking MCM-10 are viable but are highly sensitive to depletion of DNSN-1 (Figure 5). A further possibility would be that DNSN-1 is required to maintain the integrity of CMG at replication forks, but we disfavor this idea for several reasons. For example, mutation or depletion of DNSN-1 does not impair fork progression (Figure 2F, Figure 8C), recombinant CMG is inherently stable in the absence of DNSN-1, and a reconstituted human replisome supports efficient DNA synthesis in the absence of DONSON (25). Our data do not exclude some other role for DNSN-1/DONSON at replication forks, such as a role in the repair of inter-strand DNA crosslinks as reported previously for human DONSON (39). However, a recent study (55) has found that human DNA polymerase α-primase binds to CMG in a manner that would clash with DONSON binding (Figure S18), indicating that DONSON cannot remain bound to CMG at active forks in the same configuration observed by cryoEM.

Much remains to be learned in the future regarding the mechanism of metazoan DNA replication initiation, which clearly involves considerable additional complexity beyond the model established for budding yeast. Further insights in this area will inform our understanding of how DNA replication initiation is deregulated during tumor development, as well as providing further mechanistic insight into how DNA replication initiation defects might underpin the development of human disease syndromes that are associated with mutations in DONSON (33, 34) and the SLD-2 orthologue RECQL4 (56, 57).

## Materials And Methods

### Experimental Model And Subject Details

The *C. elegans* strains used in this study were derived from the ‘Bristol N2’ wild type and are described in Table S5. Alleles generated via the CRISPR-Cas9 genome editing system (InVivo Biosystems & Suny Biotech) were subsequently out-crossed eight times with the N2 wild type.

For expression of proteins in budding yeast, and as detailed in Table S5, one of the three *Saccharomyces cerevisiae* strains yJF1 (*MAT***a** *ade2-1 ura3-1 his3-11,15 trp1-1 leu2-3,112 can1-100 bar1Δ::hphNT pep4Δ::kanMX*), YSS3 (*MAT***a** *ade2-1 ura3-1 his3-11,15 trp1-1 leu2-3,112 can1-100 pep4Δ::ADE2*) or YSS4 (*MATa ade2-1 ura3-1 his3-11,15 trp1-1 leu2-3,112 can1-100 pep4Δ::ADE2*) was transformed with the indicated linearised plasmids using standard procedures. The codon usage of the expression constructs was optimised for high-level expression in *Saccharomyces cerevisiae*, as described previously (7). The codon optimized DNA sequences were synthesized by GenScript.

For expression of proteins in bacteria, the plasmids listed in Table S5 were transformed into the *E. coli* strain Rosetta™ (DE3) pLysS (70956, Novagen).

### Method Details

#### *C. elegans* maintenance

Worms were maintained according to standard procedures (58) and were grown on ‘Nematode Growth Medium’ (NGM: 3 g/l NaCl; 2.5 g/l peptone; 20 g/l agar; 5 mg/l cholesterol; 1 mM CaCl_2_; 1 mM MgSO_4_; 2.7 g/l KH_2_PO_4_; 0.89 g/l K_2_HPO_4_).

#### RNA interference

RNAi was performed by feeding worms with bacteria containing plasmids that express double-stranded RNA. RNAse III-deficient HT115 bacteria were transformed with an indicated L4440-derived plasmid. For microscopy experiments, worms were fed on 6cm plates containing the following medium: 3 g/l NaCl, 20 g/l agarose, 5 mg/l cholesterol, 1 mM CaCl_2_, 1 mM MgSO_4_, 2.7 g/l KH_2_PO_4_, 0.89 g/l K_2_HPO_4_, 1 mM IPTG and 100 mg/l Ampicillin. For immunoprecipitation experiments, worms were fed on 15cm plates containing NGM medium supplemented with 1 mM IPTG and 100 mg/l Ampicillin.

The plasmids expressing dsRNA were either derived from a commercial RNAi library (SourceBioscience, UK; *clsp-1*), or else were made by cloning PCR products into the vector L4440. In the latter case, we either amplified 1kb products from cDNA (*npl-4.2 isoform a*, *tim-1*, *dnsn-1c*, *sld-2*, *tres-1*, *mus-101*), or amplified full-length cDNA for open reading frames of *cdc-45* gene, or amplified full-transcript of *GFP-*tag sequences from KAL213, using a cDNA library that was kindly provided by Sarah-Lena Offenburger and Giulia Saredi. Details of sequences used in the RNAi vectors are provided in the Table S5.

To target more than one gene simultaneously by RNAi, as indicated above we either fed a mixture of bacteria expressing the corresponding dsRNA or else cloned contiguous 1kb fragments for each gene into a single L4440 plasmid. Empty L4440 vector was used as the control for RNAi experiments throughout this study.

#### Microscopy

Worms at the larval L4 stage were incubated on 6 cm RNAi feeding plates for 48 hours at 20°C. Adult worms were then dissected in M9 medium (6 g/L Na_2_HPO_4_, 3 g/L KH_2_PO_4_, 5 g/L NaCl, 0.25 g/L MgSO_4_) and five embryos were transferred onto a 2% agarose pad and recorded simultaneously from the one-cell stage to four cells. To record embryos during early S-phase, 1 or 2 embryos were transferred onto a thick 1% agarose pad and filmed until first mitosis and the procedure was repeated to obtain 5 embryos.

For the experiment in Figure 8E-F, we generated heterozygote *gfp-dnsn-1/mCherry-dnsn-1-3A* hermaphrodites, to allow the phenotype of *mCherry-dnsn-1-3A* to be examined after depletion of wild type GFP-DNSN-1 by *GFP* RNAi, since the *mCherry-dnsn-1-3A* allele is lethal when homozygous. As a control, we performed similar experiments with heterozygote *gfp-dnsn-1/mCherry-dnsn-1* hermaphrodites, in which both tagged versions of *dnsn-1* lacked the 3A mutations in the binding site of DNSN-1 with the AAA+ domain of MCM-3. To generate these strains, we crossed *gfp-dnsn-1/gfp-dnsn-1* males to *mCherry-dnsn-1-3A/dnsn-1* hermaphrodites or *mCherry-dnsn-1/mCherry-dnsn-1* hermaphrodites. The resulting *gfp-dnsn-1/mCherry-dnsn-1* and *gfp-dnsn-1/mCherry-dnsn-1-3A* heterozygote hermaphrodites were then treated with RNAi to *cdc-45* + *GFP*, or empty RNAi vector, before dissection of embryos. Only the embryos expressing mCherry-DNSN-1 (wild type or DNSN-1-3A) were analyzed.

Time lapse images were recorded at 24°C as described previously (35) taking images every 10 seconds, using a Zeiss Cell Observer SD microscope with a Yokogawa CSU-X1 spinning disk and a HAMAMATSU C13440 camera, fitted with a PECON incubator, a 60X/1.40 Plan Apochromat oil immersion lens (Olympus). A single optical section (z-layer) was imaged for each time point.

Images were captured using the ZEN blue software (Zeiss), processed and analyzed with ImageJ software (National Institute of Health). For each time-lapse experiment, the raw images were cropped, the intensity scale was adjusted similarly for all conditions, the ‘bit depth’ was changed from 16-bits to 8-bits and videos were assembled. For selected timepoints, images from videos were further cropped to focus on the ‘female’ nucleus or nuclei, or on the entire embryo. Each series of images was then combined into a contiguous sequence, and the images were subjected to Gaussian Blur with a radius of 1 pixel. Subsequently, the pixel density was adjusted to 300 dots per inch.

The signal intensity for mCherry-PSF-1 in Figure S8 was quantified using imageJ, by calculating the integrated density of an area containing the metaphase plate and then subtracting the background (integrated density of another area of the embryo that was the same size but lacked chromatin). The mean value for five embryos was then determined, together with the standard deviation.

#### Viability and synthetic lethality analysis in *C. elegans*

For synthetic lethal analysis involving RNAi, RNAse III-deficient HT115 bacteria were transformed with an L4440-derived plasmid, corresponding to the required RNAi treatment. For the experiment in Figure 2B, the bacterial cultures expressing *dnsn-1* or *gfp* double-stranded RNA or containing an empty plasmid were used to feed N2 or *GFP-dnsn-1* worms. For the experiment in Figure 5, the bacterial cultures expressing *dnsn-1*, *sld-2*, *tres-1* or *mus-101* double-stranded RNA or containing an empty plasmid were used to feed N2, *mcm-10Δ*, or *cdc-7Δ* worms. For Figure 8D, bacterial cultures expressing *dnsn-1*, *sld-2*, *tres-1* or *mus-101* double-stranded RNA or containing an empty plasmid were used to feed N2 or *dnsn-1ΔN* worms. All cultures were grown to OD600 = 1, and worms were then incubated on RNAi feeding plates for 48 hours at 20°C. For each condition, triplicate experiments were performed, in each of which 5 adult worms were allowed to produce embryos on a plate during a period of 180 minutes, after which the adults were removed, and the embryos were counted. Two days later, the number of embryos that had developed into viable adults was determined (between 50 and 150 embryos for each set of embryos from 5 worms). Embryonic viability was expressed as the ratio between the number of viable embryos and the total number of embryos, and the average and standard deviation were then determined for each triplicate set.

Viability of gene deletions, or viability of CRISPR-generated mutations in *dnsn-1*, was monitored by counting the progeny of worms that were subsequently genotyped by PCR, to confirm homozygosity. Note that the maternal contribution of a gene is often sufficient to support completion of embryogenesis, while the zygotic contribution is only required when the maternal contribution has runout, often after embryogenesis. An RNAi experiment depletes the maternal contribution, leading to embryonic lethality if the gene is essential and depletion is sufficient.

In contrast, a null mutant of an essential gene (e.g. complete deletion of the coding sequence by CRISPR-Cas9) only removes the zygotic contribution to a homozygous embryo from a heterozygote parent. This can lead to larval death when the maternal contribution eventually runs out, or to adult sterility, since cell division in the adult is restricted to the germline.

#### Protease inhibitor cocktails

The following cocktails of protease inhibitors were used as indicated in the sections below:

(1) 1X cocktail corresponded to one Roche EDTA free protease inhibitor tablet (000000011873580001, Roche) per 25 ml of buffer (one tablet dissolved in 1 ml water makes a 25× stock solution), plus 1 ml of Sigma protease inhibitor cocktail (P8215, Sigma-Aldrich) per 100 ml of buffer.
(2) 1X cocktail corresponded to one Roche EDTA free protease inhibitor tablet (000000011873580001, Roche) per 25 ml of buffer (one tablet dissolved in 1 ml water makes a 25× stock solution), together with 0.5 mM PMSF, 5 mM benzamidine HCl, 1 mM AEBSF (A8456, Sigma-Aldrich) and 1 mg/ml Pepstatin A (P5318, Sigma-Aldrich).
(3) 1X cocktail corresponded to one Roche EDTA free protease inhibitor tablet (000000011873580001, Roche) per 25 ml of buffer (one tablet dissolved in 1 ml water makes a 25× stock solution), plus 0.5 mM PMSF.

#### Extracts of worm embryos and immunoprecipitation of worm replisome

RNAse III-deficient HT115 bacteria were transformed with an L4440-derived plasmid, corresponding to the required RNAi treatment. A 10ml pre-culture was then grown overnight and used to inoculate a 450 ml culture in ‘Terrific Broth’ (12 g/l Tryptone, 24 g/l yeast extract, 9.4 g/l K_2_HPO_4_, 2.2 g/l KH_2_PO_4_, adjusted to pH 7). After 7 hours of growth in a baffled flask at 37°C with agitation, expression of dsRNA was induced overnight at 20°C by addition of 3mM IPTG. The bacteria were then pelleted and resuspended with one-fifth volume of 5xLCS buffer (M9 medium supplemented with 75 mg/l cholesterol; 100 mg/l ampicillin; 50 mg/l tetracycline; 12.5 mg/l amphotericin B; 3 mM IPTG).

For each experiment, 0.7ml of a synchronized population of L4 worms expressing TAP-PSF-1 (Table S3), GFP-PSF-1 (Figure 1A-B, Figure S2A-B, Figure 6A and Table S1), GFP-DNSN-1 (Figure 1B-C, S2C-D and Table S2), DNSN-1-GFP (Figure 1B, S2C-D and Table S2) or mCherry-DNSN-1 (Figure 8B) were fed for 50 hours at 20°C on a 15 cm RNAi plate (see above), supplemented with 10 g of bacterial pellet for the required RNAi treatment, prepared as described above. After feeding, the adult worms were washed in M9 medium and resuspended for 2 minutes at room temperature in 14 ml of ‘bleaching solution’ (for 100 ml: 36.5 ml H2O, 45.5 ml 2 M NaOH and 7 ml NaClO 10%), then pelleted for 1 minute at 300 g. This bleaching procedure was repeated two more times, corresponding to a total of 12 minutes in bleaching solution, to lyse the adult worms and release embryos (about 0.6-0.8 g). After bleaching, the embryos were washed twice with M9 medium.

The remaining steps were performed at 4°C and are previously described methods (35). Embryos were washed twice with lysis buffer (100 mM HEPES-KOH pH 7.9, 100 mM potassium acetate, 10 mM magnesium acetate, 2 mM EDTA, 0.02% IGEPAL CA-630, 10% glycerol), and then resuspended with three volumes of lysis buffer that was supplemented with 2 mM sodium fluoride, 2 mM sodium β-glycerophosphate pentahydrate, 1 mM dithiothreitol (DTT), 1X Protease Inhibitor Cocktail 1 and 5 *μ*M Propargylated ubiquitin (Ub-PrG) to inhibit de-ubiquitylase enzymes (kindly provided by Axel Knebel and Clare Johnson; DU49003, MRC PPU reagents and services). The mixture was transferred dropwise into liquid nitrogen to prepare ‘popcorn’, which was stored at -80°C. We then ground ~ 2.5 g of the frozen popcorn in a SPEX SamplePrep 6780 Freezer/Mill. After thawing, we added one-quarter volume of lysis buffer (with additional 1 mM DTT, 2 mM sodium fluoride, 2 mM sodium β-glycerophosphate pentahydrate, plus 1X protease inhibitor cocktail 1). Chromosomal DNA was digested with 1600 U of Universal Nuclease (Pierce™, 88702, ThermoFisher Scientific) for 30 minutes at 4°C. Extracts were centrifuged at 25000 x g for 30 minutes and then at 100000 x g for 1 hour. 50 *μ*l of extract was added to 100 *μ*l of 1.5 X Laemmli buffer and stored at -80°C. The remaining ~2 ml of extract was then incubated for 90 minutes with 40 *μ*l slurry of GFP-Trap Magnetic Particles M-270 (gtd, Chromotek), RFP-Trap Magnetic Particles M-270 (rtdk, Chromotek) or 200 *μ*l slurry of magnetic beads (Dynabeads M-270 Epoxy; 14302D, ThermoFisher Scientific) coupled to rabbit immunoglobulin G (S1265, Sigma-Aldrich) as described below. The beads were washed four times with 1 ml of wash buffer (lysis buffer supplemented with 1 mM DTT, 2 mM sodium fluoride, 2 mM sodium β-glycerophosphate pentahydrate, plus 1X protease inhibitor cocktail 1) and the bound proteins were eluted at 95°C for 5 min in 100 *μ*l of 1X Laemmli buffer (or 50 *μ*l when used for mass spectrometry analysis) and stored at -80°C.

#### Mass spectrometry

Samples were purified from worm embryos as above and eluted in 50 *μ*l Laemmli buffer, of which 30*μ*l was resolved by SDS-polyacrylamide gel electrophoresis (SDS-PAGE) using NuPAGE Novex 4-12% Midi Bis-Tris gels (NP0321, Life Technologies) with NuPAGE MOPS SDS buffer (NP000102, Life Technologies). Subsequently, gels were stained with ‘SimplyBlue SafeStrain’ colloidal Coomassie (LC6060, Invitrogen), and each lane was cut into 40 slices that were digested with trypsin before processing for mass spectrometry (MS Bioworks, USA). Data were analyzed using Scaffold software (Proteome Software Inc, USA).

#### Purification of *C. elegans* proteins

Proteins purified in this study are listed in the Table S5. TEV protease (DU6811, MRC PPU Reagents and Services) and PreScission (DU34905, MRC PPU Reagents and Services) protease were kindly provided by Dr. Axel Knebel. The proteins were produced as described in the following sections. The other proteins were produced as described in the following sections, using the buffer below: Buffer A: 25 mM Hepes KOH pH 7.6, 10% glycerol, 0.02% IGEPAL CA-630, 1 mM TCEP.

#### Expression of proteins in budding yeast

The *Saccharomyces cerevisiae* strains used in this study are shown in Table S5. Yeast cells were grown at 25°C in YP medium (1 % Yeast Extract, 21275, Becton Dickinson; 2 % bacteriological peptone, LP0037B, Oxoid) supplemented with 2 % Raffinose. In each case, a 12-litre exponential culture was grown to 2-3 x 10^7^ cells/ml and then induced for 6 h at 20°C by addition of Galactose to a final concentration of 2 %. Cells were collected by centrifugation and washed once with lysis buffer (indicated below for each purification) lacking protease inhibitors. Cell pellets (~ 30 g) were then resuspended in 0.3 volumes of the indicated lysis buffer containing protease inhibitors. The resulting suspensions were then frozen dropwise in liquid nitrogen and stored at – 80°C. Subsequently, the entire sample of frozen yeast cells were ground in the presence of liquid nitrogen, using a SPEX CertiPrep 6850 Freezer/Mill with 3 cycles of 2’ at a rate of 15. The resulting powders were then stored at – 80 °C.

##### CMG helicase

Purification of CMG was modified as previously described (35). Yeast cell powder was thawed in buffer A/0.2 M KCl/2 mM Mg(OAc)_2_/1X protease inhibitor cocktail 2. Universal Nuclease (Pierce™, 88702, ThermoFisher Scientific) was then added to 250 U/ml to the whole cell extract and the sample was incubated at 4 °C for 0.5 h with rotation. The mixture was centrifuged at 100,000 x g at 4°C for 0.5 h, followed by another step of centrifugation at 235,000 x g at 4°C for 1 h. After spinning, the soluble extract was recovered and mixed with 2 ml IgG resin (17096901, GE). The mixture was incubated at 4 °C for 2 h with rotation.

Resin was collected and washed extensively with buffer A/0.2 M KCl/2 mM Mg(OAc)_2_/1X protease inhibitor cocktail 2. The resin was then incubated with 10ml buffer A/0.2 M KCl/10 mM Mg(OAc)_2_/1X protease inhibitor cocktail 2/2 mM ATP at 4 °C for 10 minutes to remove chaperones, then washed extensively with buffer A/0.2 M KCl/2 mM Mg(OAc)_2_. The purified proteins were then eluted by overnight incubation with rotation in 2 ml buffer A/0.2 M KCl/2 mM Mg(OAc)_2_ containing 100 *μ*g PreScission protease.

The supernatant was collected, and the resin was further eluted twice with 2ml buffer A/0.2 M KCl/2 mM Mg(OAc)_2_. The pooled eluate was diluted to 10 ml and loaded onto a 1 ml HiTrap Q column in buffer A/0.2 M KCl/2 mM Mg(OAc)_2_. CMG was eluted with a 30 ml gradient from 0.2 – 1 M KCl in buffer E/2 mM Mg(OAc)_2_. The peak fractions were concentrated and loaded onto a 24 ml Superose 6 column in buffer A/0.3 M KOAc/2 mM Mg(OAc)_2_. Peak fractions containing CMG were pooled, aliquoted, and snap frozen in liquid nitrogen and stored at – 80 °C.

##### GINS

Yeast cell powder was thawed in buffer A/0.5 M NaCl/1X protease inhibitor cocktail 2. Universal Nuclease (Pierce™, 88702, ThermoFisher Scientific) was then added to 250 U/ml to the whole cell extract and the sample was incubated at 4 °C for 0.5 h with rotation. The mixture was centrifuged at 100,000 x g at 4°C for 0.5 h, followed by another step of centrifugation at 235,000 x g at 4°C for 1 h. After spinning, the soluble extract was recovered and mixed with 2 ml IgG resin (17096901, GE). The mixture was incubated at 4 °C for 2 h with rotation.

Resin was collected and washed extensively with buffer A/0.5 M NaCl/1X protease inhibitor cocktail 2. The resin was then incubated with 10ml buffer A/0.5 M NaCl/10 mM Mg(OAc)_2_/1X protease inhibitor cocktail 2/2 mM ATP at 4 °C for 10 minutes to remove chaperones, then washed extensively with buffer A/0.5 M NaCl. The purified proteins were then eluted by overnight incubation with rotation in 2 ml buffer A/0.5 M NaCl containing 100 *μ*g PreScission protease.

The supernatant was collected, and the resin was further eluted twice with 2ml buffer A/0.5 M NaCl. The pooled eluate concentrated and loaded onto a 24 ml Superdex 200 column in buffer A/0.5 M NaCl. Peak fractions containing CMG were pooled, aliquoted, and snap frozen in liquid nitrogen and stored at – 80 °C.

##### TIM-1 TIPN-1

Purification of TIM-1_TIPN-1 was carried out as previously described (59). Briefly, yeast cell powder was thawed in buffer A/0.2 M NaCl/1X protease inhibitor cocktail 2. After centrifugation, the soluble yeast extract was mixed with calmodulin affinity resin(17052901, GE) and TIM-1_TIPN-1 was subsequently eluted in buffer A/0.2 M NaCl/2mM EDTA/2mM EGTA. The purified proteins were then eluted with addition of 100 μg PreScission protease to the eluate fraction, followed by overnight incubation with rotation at 4 °C. The purified sample was concentrated and loaded onto a 24 ml Superdex 200 column in buffer A/0.3 M KOAc. The peak fractions were then pooled, concentrated and reloaded onto a 24 ml Superdex 200 column in buffer A/0.3 M KOAc. Finally, the peak fractions were pooled, concentrated, aliquoted and snap frozen in liquid nitrogen, before storage at - 80 °C.

#### Expression of proteins in bacterial cells

The plasmids for bacterial expression used in this study were shown in Table S5. Each plasmid was transformed into Rosetta (DE3) pLysS (70956, Novagen), which was grown in LB medium supplemented with 50 *μ*g/ml ampicillin (pET15b based plasmids) or 50 *μ*g/ml kanamycin (pK27SUMO based plasmids). Subsequently, a 10 ml culture was grown overnight at 37°C with shaking at 200 rpm. The following morning, the culture was diluted 50-fold into 500 ml of selective medium and then left to grow at 37°C until an OD600 of 1 was reached. At this point, 1 mM IPTG was added and expression was induced overnight at 18°C. Cells were harvested by centrifugation for 10 min in a JLA-9.1000 rotor (Beckman) at 5000 rpm. The cell pellets were then stored at -80 °C.

#### DNSN-1 (full-length and truncated proteins)

Pellets were resuspended in 20 ml of buffer A/0.5 M NaCl/20 mM imidazole/1X protease inhibitor cocktail 3 with 500 *μ*g/ml Lysozyme, then the mixture was incubated at 4 °C for 0.5 h with rotation. Subsequently, the sample was sonicated twice for 90 s (15 s on, 30 s off) at 40% on a Branson Digital Sonifier. The mixture was centrifuged at 100,000 x g at 4°C for 0.5 h. After spinning, the soluble extract was recovered and mixed with 1 ml Ni-NTA resin (30210, QIAGEN). The mixture was incubated at 4 °C for 2 h with rotation.

Resin was collected and washed extensively with buffer A/0.5 M NaCl/20 mM imidazole/1X protease inhibitor cocktail 3. The resin was then incubated with 10ml buffer A/0.5 M NaCl/20 mM imidazole/1X protease inhibitor cocktail 3/10 mM Mg(OAc)_2_/2 mM ATP at 4 °C for 10 minutes to remove chaperones, and then washed extensively with buffer A/0.5 M NaCl/20 mM imidazole. Proteins were eluted with 5 ml of buffer A/0.5 M NaCl/250 mM imidazole. Ulp1 protease (10 μg/ml) was added to cleave the His-SUMO tag from UBC-12 and the mixture was incubated for 1 h on ice.

The sample was concentrated and loaded onto a 24 ml Superdex 200 column in buffer A/0.5 M NaCl. The peak fractions were pooled, concentrated, aliquoted, snap frozen in liquid nitrogen and stored at - 80 °C.

#### Isolation of *C. elegans* embryonic cells

RNAse III-deficient HT115 bacteria were transformed with an L4440-derived plasmid, corresponding to the required RNAi treatment. 20 ml culture in ‘Terrific Broth’. After 7 hours of growth in a baffled flask at 37°C with agitation, expression of dsRNA was induced overnight at 20°C by addition of 3mM IPTG. The bacteria were then pelleted and resuspended with one-fifth volume of 5X LCS buffer.

For each experiment, 10 *μ*l of a synchronized population of L4 worms were fed for 48 hours at 20°C on a 6 cm NGM plate, supplemented with bacterial pellet for the required RNAi treatment, prepared as described above. After feeding, the adult worms were washed in M9 medium and resuspended for 10 minutes at room temperature in 1 ml of ‘bleaching solution’, then pelleted for 1 minute at 300 g. Release embryos were washed twice with M9 medium and resuspended in 1 ml of Dulbecco’s Modified Eagle Medium (DMEM, 11960044, Thermo Fisher), supplemented with 10% Fetal Bovine Serum (FBS, FCSSA/500, LabTech). To remove the eggshell of the embryos, 16 *μ*l of Chitinase (25 U/ml, C6137-25UN, Sigma-Aldrich) was added and gently rotated for 45 minutes at room temperature. Cells were dissociated by gently pipetting for 3 minutes. The remaining embryos and larvae were removed by 4 *μ*m filter.

#### EdU cell proliferation assay

Embryonic cells were isolated as described above. Cells were incubated with 20 *μ*M 5-Ethynyl-2’-deoxyuridine (EdU) for 20 minutes at room temperature with gentle rotation. Cells were pelleted for 1 minute at 1000 g, 4°C, then washed in 1 ml cold PBS and resuspended. Cells were pelleted for 1 minute at 1000 g, 4°C, then resuspended within 20 *μ*l cold PBS. 5 *μ*l cells slurry was transferred on a poly-lysine coated slide, covered by a coverslip, and then quickly froze the slide on dry ice. The coverslip was removed before the fixation of the slide in cold methanol. The slides were fixed in methanol overnight at -20°C.

The slides were thawed in PBS for 10 minutes at room temperature, additional PBS was removed from the slides. The incorporated EdU was then labelled using the “Click-iT™ Plus Alexa Fluor™ 647 Picolyl Azide Toolkit” (C10643, Invitrogen), according to the manufacturer’s instructions. Nuclear DNA was stained with 5 μg/ml Hoechst 33342 (H1399, Invitrogen) for 10 minutes at room temperature. The slides were washed twice by PBS/T (PBS supplemented with 0.1% Tween) followed by PBS for 5 minutes each time at room temperature. The slides were air-dried, mounted in 90 % glycerol in PBS, and sealed. Microscopy was performed using a Zeiss Cell Observer SD microscope with a Yokogawa CSU-X1 spinning disk and a HAMAMATSU C13440 camera, fitted with a PECON incubator. Images were captured using the ZEN blue software (Zeiss) and analyzed with FIJI software (National Institute of Health). More than one hundred Hoechst-positive cells were detected, The EdU-positive population was calculated by dividing the number of cells positive for EdU-Alexa Fluor647 by the total number of Hoechst-positive cells. The average and standard deviation were then determined for each triplicate set.

#### Molecular combing of DNA fibers

Embryonic cells were isolated as described above. Cells were incubated with 200 *μ*M EdU for 30 minutes at room temperature with gentle rotation. Cells were pelleted for 1 minute at 1000 g, 4°C, then washed in 1 ml cold PBS and resuspended. Cells were pelleted for 1 minute at 1000 g, 4°C, then resuspended within 90 *μ*l cold PBS. Combing was performed with the FiberPrep^®^ DNA extraction Kit (EXT-001A, Genomics Vision). A 90 *μ*l aliquot of the cell slurry was used to prepare two agarose plugs and subjected to Proteinase K digestion overnight at 60°C. The following day, the plugs were washed three times with a kit wash buffer for one hour per wash and followed by an additional wash step for 3 hours. The two plugs from the same sample were then transferred to 2 ml, round bottom tubes (HP37094D, Eppendorf) and stained by YOYO-1 in kit staining buffer for one hour at room temperature. Then plugs were melted at 68°C for 20 minutes, before subsequently equilibration at 42°C for 10 minutes, after which β-agarase was added and the samples were incubated for 14 hours at 42°C in the dark. The following day, DNA fibers were combed onto silanized CombiCoverslips™ coverslips (COV-002-RUO, Genomics Vision) with FiberComb^®^ Molecular Combing System (MCS-001, Genomics Vision). The combed coverslips were heated at 60°C for 2 hours.

For detection of labelled DNA, the coverslips were dehydrated consecutively in ethanol at 70%, 90%, and 100%, for 1 minute each. Air-dried coverslips were blocked by block-aid (B10710, Invitrogen) at 37°C for 30 minutes. The incorporated EdU was then labelled using the “Click-iT™ Plus Alexa Fluor™ 647 Picolyl Azide Toolkit” (C10643, Invitrogen), according to the manufacturer’s instructions. The coverslips were washed 3 times by PBS/T for 5 minutes each time at room temperature, dehydrated in ethanol 70%, 90%, and 100%, air dried, mounted in 90% glycerol in PBS, and sealed. Microscopy was performed using a Zeiss Cell Observer SD microscope with a Yokogawa CSU-X1 spinning disk and a HAMAMATSU C13440 camera, fitted with a PECON incubator. Images were captured using the ZEN blue software (Zeiss) and analyzed with FIJI software (National Institute of Health). Fork progression was defined as the length of EdU labelled tracks, 200 EdU labelled DNA fibers were measured per sample. The inter-fork distance is defined as the distance between two EdU labelled tracks on the same fiber, 200 inter-fork distances were measured per sample. The average of the medians of each experiment and standard deviation were then determined for each triplicate set.

#### Immunoprecipitation of reconstituted complexes

Reactions (typically 10 *μ*l in volume) containing 25 mM Hepes-KOH (pH 7.6), 0.02% IGEPAL CA-630, 0.1 mg/ml BSA, 1 mM DTT, 100mM KOAc, 10 mM Mg(OAc)_2_, 0.5 mM AMP-PNP, 50nM leading DNA substrate (comprising 46bp double-stranded DNA and a 39nt ‘3’-flap’ of single-stranded DNA - the sequences are shown in Table S5) and 3.3 *μ*l protein mix were assembled on ice for 30 minutes. The protein mix contained 300 mM KOAc, so the final KOAc concentration of the reactions was 200 mM. Each sample was then incubated at 4°C with 5 *μ*l magnetic beads (Dynabeads M-270 Epoxy; 14302D, ThermoFisher Scientific) that had been coupled to anti-SLD-5 antibodies as described below. After 1 hour, protein complexes bound to the magnetic beads were washed twice with 1 ml of buffer containing 25 mM Hepes-KOH (pH 7.6), 0.02% IGEPAL CA-630, 0.1 mg/ml BSA, 1 mM DTT, 10 mM Mg(OAc)_2_ and 200 mM KOAc. The bound proteins were eluted at 95°C for 5 minutes in 30 *μ*l of 1X Laemmli buffer.

For the experiment in Figure 6D, a 10 *μ*l volume with the indicated components corresponding to 15 nM CMG, 15 nM GINS, and 30 nM DNSN-1 (as a dimer). For the experiment in Figure 8A, a 10 *μ*l volume with the indicated components corresponding to 15 nM CMG and 50 nM DNSN-1 variants (as a dimer).

#### Glycerol gradient analysis

Protein mixtures were prepared in 25 mM Hepes-KOH (pH 7.6), 200 mM KOAc, 0.02% IGEPAL CA-630, 1 mM DTT, 10 mM Mg(OAc)_2_, 0.5 mM AMP-PNP, 500 nM leading DNA substrate and then incubated on ice for one hour. To assemble glycerol gradients, five different concentrations of glycerol buffers were used (10%, 15%, 20%, 25%, 30%), each containing 25 mM Hepes-KOH (pH 7.6), 200 mM KOAc, 0.02% IGEPAL CA-630, 1 mM DTT, 10 mM Mg(OAc)_2_. Gradients were assembled by consecutively layering 40 *μ*l of each of the five concentrations of glycerol buffers (30% to 10%) in an ultra-centrifuge tube (P200915MGSG, Beckman). Subsequently, 5 *μ*l of the protein mixture was added to the top of the gradient, before spinning for one hour at 55,000 rpm (249,000 x g in a Beckman TLS55 rotor) at 4°C. Ten fractions of 20 *μ*l each were then collected from top to the bottom of the gradient. After addition of 10 *μ*l 3X Laemmli buffer, the samples were analyzed by SDS-PAGE and immunoblotting.

#### Immunoblotting

Protein samples were resolved by SDS–PAGE using the following systems: NuPAGE Novex 4 - 12% Bis-Tris gels (NP0321 and WG1402A, ThermoFisher Scientific) with NuPAGE MOPS SDS buffer (NP0001, ThermoFisher Scientific) or NuPAGE MES SDS buffer (NP0002, ThermoFisher Scientific); NuPAGE Novex 3 - 8% Tris-Acetate gels (EA0375BOX and WG1602BOX, ThermoFisher Scientific) with NuPAGE Tris-Acetate SDS buffer (LA0041, ThermoFisher Scientific). The resolved proteins were either stained with colloidal Coomassie blue dye (‘Instant Blue’, ab119211, Abcam), or else were transferred onto a nitrocellulose iBlot2 membrane (2NR290123-01, ThermoFisher Scientific) with the iBlot2 Dry Transfer System (IB21001, Invitrogen), according to the manufacturer’s instructions.

The antibodies used for immunoblotting in this study are described in Table S5. Chemoluminescent signals were detected by azure-biosystems 300Q with ECL Western Blotting Detection Reagent (17039552, GE Healthcare).

#### Preparation of antibody-coated magnetic beads

A slurry of activated magnetic beads (Dynabeads M-270 Epoxy; 14302D, ThermoFisher Scientific) was prepared by resuspending 300 mg beads in 10 ml dimethyl formamide. Each coupling reaction involved 425 *μ*l slurry of activated magnetic beads, which corresponded to ~ 1.4 x 10^9^ beads. After removing the supernatant, the beads were washed twice with 1 ml of 0.1M NaPO_3_ pH7.4. Subsequently, the beads were incubated with 300 *μ*g of rabbit immunoglobulin G (S1265, Sigma-Aldrich), *C. elegans* MCM-6 antibody (SA417, MRC PPU Reagents and Services), or *C. elegans* SLD-5 antibody (SA419, MRC PPU Reagents and Services), 300 *μ*l of 3M (NH_4_)2SO_4_, plus 0.1M NaPO_3_ pH 7.4 up to a total volume of 900 *μ*l. The mixture was then incubated at 4 °C for 2 days with rotation.

Subsequently, the supernatant was removed, and the beads were washed four times with 1 ml PBS. The beads were then incubated for 10 minutes in 1 ml PBS/0.5% IGEPAL CA-630 with rotation at room temperature, before washing twice with 1 ml PBS. Finally, the washed beads were resuspended with 900 *μ*l PBS containing 5 mg/ml BSA.

#### Yeast two-hybrid assays

Two-Hybrid analysis based on the Gal4 transcription factor was performed by co-transformation of derivatives of pGADT7 (630442, Takara; Gal4 activation domain; LEU2 marker) and pGBKT7 (630443, Takara; Gal4 DNA binding domain; TRP1 marker) into the yeast strain PJ69–4A. For transformation and selection, synthetic complete dropout medium (SC-media) was used with the required supplements. For each assay, five independent transformed colonies were mixed together in dH2O and used to make serial dilutions, before spotting 10-fold dilutions from 50 000 to 50 cells onto SC medium lacking tryptophan and leucine (selective for pGADT7 and pGBKT7, but non-selective for the two-hybrid interaction) or SC medium lacking tryptophan, leucine, histidine (selective for the two-hybrid interaction).

#### Cryo-EM sample preparation

The DNA substrate was annealed by mixing equal volumes of the leading strand template (5’-[Cy3]TAGAGTAGGAAGTGA[iBiodT]GGTAAGTGATTAGAGAATTGGAGAGTGTGTTTTTTTTTTTTTTTTTTTTTTTTTTTTTTTTTTT*T*T*T*T*T; *=phosphorothioate linkage) and the lagging strand template (5’-GGCAGGCAGGCAGGCACACACTCTCCAATTCTCTAATCACTTACCA[iBiodT]CAC TTCCTACTCTA), each at 53 μM in 25 mM HEPES-NaOH, pH 7.5, 150 mM NaOAc, 0.5 mM TCEP, 2 mM Mg(OAc)_2_, before allowing to cool gradually from approx. 90°C to room temperature.

CMG was mixed with 1.3-fold molar excess DNA in reconstitution buffer (25 mM HEPES-NaOH, pH 7.5, 100 mM NaOAc, 0.5 mM TCEP, 10 mM Mg(OAc)_2_, 0.5 mM AMP-PNP) in a 100 μL reaction volume, and incubated on ice for 30 min. To this was added a mixture of TIM-1/TIPN-1 and DNSN-1 isoform c (each at 1.3-fold molar excess over CMG) in reconstitution buffer giving a final reaction volume of 250 μL and resulting in a final CMG concentration of 125 nM. The reaction was incubated on ice for a further 30 min before loading 100 μL onto each of two GraFix gradients (60) (with the remainder applied to a gradient with crosslinking agents omitted). Glycerol gradients were prepared using a modified form of a previously described protocol (50), by layering equal volumes of a 10% glycerol buffer (40 mM HEPES-NaOH, pH 7.5, 100 mM NaOAc, 0.5 mM TCEP, 0.5 mM AMP-PNP, 10 mM Mg(OAc)_2_, 10% v/v glycerol) on top of a 30% glycerol buffer (40 mM HEPES-NaOH, pH 7.5, 100 mM NaOAc, 0.5 mM TCEP, 0.5 mM AMP-PNP, 10 mM Mg(OAc)_2_, 30% v/v glycerol, 0.22% glutaraldehyde, 2 mM bis(sulfosuccinimidyl)suberate) in a 2.2 mL TLS-55 centrifuge tube (Beranek Laborgerate). Gradients were prepared using a gradient-making station (Biocomp Instruments, Ltd.) before cooling on ice.

Gradient sedimentation was performed by centrifugation using a Beckman TLS-55 rotor (200,000*g*, 2 h, 4°C) and 100 μL fractions manually collected. After analysis of each fraction by silver-stained SDS-PAGE, peak fractions (fractions 7-9 in Figure S11A) were pooled across both crosslinking gradients (total volume ~550 μL) and buffer exchanged in buffer containing 25 mM HEPES-NaOH, pH 7.5, 150 mM NaOAc, 0.5 mM TCEP, 2 mM Mg(OAc)_2_, 0.1 mM AMP-PNP, 0.005% v/v TWEEN 20 [Sigma, Cat#P8341] during six rounds of ultrafiltration in 0.5 mL 30K MWCO centrifugal filters (Amicon) using a bench-top centrifuge (21,000*g*, 4°C, 1 min/round). Sample was concentrated to ~27 μL and immediately used for cryo-EM grid preparation using Quantifoil R2/2 copper 400-mesh grids commercially coated with a ~2nm thick ultrathin continuous carbon support, glow discharged using a PELCO easiGlow glow discharge cleaning system (15 mA, 5 s) prior to vitrification in liquid-nitrogen-cooled ethane using a cold-room situated manual plunger.

#### Cryo-EM data collection

A total of 10,825 raw micrographs were acquired in a single dataset using a 300 keV Titan Krios microscope (FEI) equipped with a K3 direct electron detector (Gatan) operated in electron counting mode using the EPU automated acquisition software (ThermoFisher) with “Faster Acquisition” mode (AFIS) enabled. A slit width of 20 eV was used for the BioQuantum energy filter. Data were collected in super-resolution mode and with a binning-factor of 2 yielding an effective pixel size of 1.09 Å/pixel (nominal magnification of 81,000 X), using a defocus range of -1.0 to -2.5 μm and dose-fractionating into 41 fractions per micrograph. An exposure time of 1.7 s achieved a dose of 40.1 e-/Å2 per micrograph.

#### Cryo-EM data processing

Data processing used RELION-4.0 (61). The 41-fraction movies were aligned and dose-weighted (0.977 e-/Å2/fraction, 5 x 5 patches, 300 Å2 B-factor) using RELION’s implementation of a MotionCor2-like program (62). CTF parameters were estimated using CTFFIND-4.1 (63). Particles were picked in a template-free manner using Gautomatch v0.56 (https://www2.mrc-lmb.cam.ac.uk/download/gautomatch-056/) leading to extraction of ~4,540,000 particles using a box size of 410 Å. During extraction, data were down-sampled to a pixel size of 4.36 Å/pixel and the dataset divided into three roughly equal parts (Parts A-C) which were subsequently processed independently up until completion of the first 3D classification without alignment, to ease computational demand (refer to Figure S12). The dataset was subjected to two iterative rounds of 2D classification (regularization parameter, T=2), yielding a total of ~3,140,000 particles in well-aligned classes. These particles were submitted for a single round of 3D classification with alignment (T=4), yielding well-aligned classes containing a total of ~3,040,000 particles representing the best-quality particles from our dataset; these classes each contained CMG helicase plus a heterogeneous occupancy of TIM-1/TIPN-1 and DNSN-1. These particles were subsequently re-extracted at a pixel size of 1.50 Å/pixel. After using 10,000 particles from one third of the dataset to determine optimized parameters, each third of the dataset was submitted for per-particle motion correction using Bayesian polishing (63); during this process, particles were re-extracted at a pixel size of 1.30 Å/pixel. The resulting particles were submitted for iterative beamtilt and trefoil correction, anisotropic magnification correction, per-particle defocus correction and per-micrograph astigmatism correction. These particles are henceforth referred to as the Polished Dataset.

To enrich the Polished Dataset for particles containing DNSN-1, signal subtraction was used to focus on the region of the map encompassing the homodimer of DNSN-1 folded domains plus neighboring regions of MCM3 and GINS that form the interface with the folded domain of DNSN-1. Subsequent 3D subclassification without alignment (T=10) yielded a total of ~170,000 particles with good DNSN-1 occupancy. After 3D refinement and map sharpening, these particles yielded a reconstruction of the CMG/DNSN-1 complex at a resolution of 3.25 Å (all resolutions henceforth quoted at the Fourier shell correlation = 0.143 criterion following 3D refinement and map sharpening with B-factors in the range -15 to -20 Å^2^). To further improve this reconstruction, multi-body refinement was used to define the following four rigid-bodies: MCM-2-7 NTDs/TIM-1/TIPN-1/dsDNA (3.20 Å resolution); MCM-2-7 AAA+/ssDNA (3.17 Å); CDC-45/GINS (2.88 Å); and DNSN-1 dimer (3.44 Å). These multi-body-derived maps were used for building models of MCM-2-7, ssDNA, CDC-45, GINS and DNSN-1.

To isolate particles containing CMG in complex with both DNSN-1 and TIM-1/TIPN-1, the above CMG/DNSN-1 particles were further 3D subclassified without alignment (T=10) after using signal subtraction to focus on the region encompassing TIM-1/TIPN-1/dsDNA. This approach produced a class containing ~33,900 particles, representing CMG/TIM-1/TIPN-1/DNSN-1 complexes (3.75 Å resolution). This reconstruction confirmed DNSN-1 and TIM-1/TIPN-1 can simultaneously associate with CMG, and each does not influence the mode of interaction of the other.

To improve the resolution of the TIM-1/TIPN-1 region of the complex, a comparable signal subtraction/3D subclassification without alignment was performed, again focusing on TIM-1/TIPN-1/dsDNA, this time using the complete Polished Dataset as the input. This approach yielded a total of ~922,000 particles representing complexes with good TIM-1/TIPN-1 occupancy (2.75 Å resolution). Multi-body refinement was used to further improve the resolution of TIM-1/TIPN-1, defining TIM-1/TIPN-1/dsDNA/MCM N-tier as a single rigid-body (2.64 Å resolution). After removing density from beyond 25 Å around TIM-1/TIPN-1 (UCSF Chimera (64), vop zone), this map was fitted to the reconstruction of the complex containing CMG/TIM-1/TIPN-1/DNSN-1 and used for TIM-1/TIPN-1/dsDNA model building.

The local resolution maps for each of the reconstructions described above were generated using RELION’s implementation of a local resolution calculation program.

#### Model building and refinement

To build the model of the CMG/TIM-1/TIPN-1/DNSN-1 complex bound to a fork DNA substrate, initial models for individual subunits were taken from the AlphaFold Protein Structure Database (65, 66) and fitted as rigid-bodies to our cryo-EM density using UCSF Chimera (64) for MCM-2-7 subunits, the NTDs, AAA+ domains and winged-helix (WH) domains were fitted separately; for both CDC45 and the complete tetrameric GINS complex, models were predicted using AlphaFold-Multimer (36, 65, 67). Additionally, the dsDNA bound by TIM-1/TIPN-1 and the ssDNA bound by the MCM-2-7 AAA+ domains were built into density using COOT (68), though the resolution of our maps was insufficient to determine the exact position of the final base pair of dsDNA. Furthermore, COOT (68) was used to add Zn^2+^ ions to the relevant MCM zinc finger domains, and AMP-PNP•Mg^2+^ ligands were built where density was observed at the interfaces between MCM-3/MCM-5, MCM-7/MCM-3, MCM-4/MCM-7 and MCM-6/MCM-4 (Figure S14A).

During this process, the MCM-3 and MCM-6 WH domains were fitted to cryo-EM density (Figure S14B) corresponding to their observed positions in a prior structure of the human CMG helicase (PDB: 6XTX (69)); additional density for a third WH domain was observed across the CMG exit channel beside MCM-5 (Figure S14C), however the local resolution was insufficient to confidently assign this to a particular MCM-2-7 subunit. A region of α-helical density was observed to interact with the PSF-2 and SLD-5 subunits of the CMG helicase; AlphaFold-Multimer (36, 70) confidently predicted the association of an N-terminal α-helix of DNSN-1 at this location and the local resolution of our cryo-EM reconstruction was sufficient to confidently build DNSN-1 residues 4-19 into this density (Figure S13A-D). Similarly, α-helical cryo-EM density with an unambiguous tryptophan side chain was observed interacting with the MCM-3 AAA+ domain (Figure S13E); this density is absent from complexes lacking DNSN-1 (Figure S12). AlphaFold-Multimer (36, 70) predicted the association of DNSN-1 residues 419-431 at this location, placing Trp425 in the tryptophan cryo-EM density (Figure S13E-H). As no other tryptophan residues present in any subunit of our complex could account for the tryptophan observed in our cryo-EM density, we were able to confidently build DNSN-1 residues 417-435. Due to distance constraints, we determine this DNSN-1 α-helix to belong to the same protomer that forms the interface with GINS and the MCM-3 NTD via its folded domain.

Finally, additional density was observed to bind the TIM-1 N-terminus, displaying some α-helical character; AlphaFold-Multimer (36, 70) predicted an interaction between this region of TIM-1 and part of the N-terminal extension of MCM-2 (Figure S14E-G), predicting placement of the M105-Y106 pair where we observe cryo-EM density for two bulky side-chains. This enabled us to build MCM-2 residues 100-111; additional density was observed nearby between TIM-1 residues 14-35 and MCM-2 residues 142-152, however the density was of insufficient resolution to confidently assign sequence (Figure S14H).

To improve the fit of the model to our cryo-EM density, COOT (68) was used to remove regions of the proteins for which density was not resolved, before using an iterative combination of ISOLDE (71) (within UCSF ChimeraX (70, 72)) and COOT to adjust and refine models to density on a residue-by-residue basis, using the best-resolved map for any given region of the complex.

After the completion of model building, the model was refined in the cryo-EM reconstruction encompassing the complete CMG/TIM-1/TIPN-1/DNSN-1 complex (3.75 Å resolution), using Phenix real-space refinement (73), enabling secondary structure restraints and using the input model as a reference to generate restraints with a sigma value of 0.1, and performing global minimisation with an nonbonded weight of 2000 and a weight of 0.5. Model validation was performed using the MolProbity server (74), Phenix Comprehensive validation (cryo-EM) (73) and the wwPDB OneDep validation server (75). Model-to-map FSCs were plotted using Xmipp (76), having used EMAN’s pdb2mrc (77) to generate a map from our model and removing solvent density from the relevant full- and half-maps using multiplication in RELION’s relion_image_handler (77).

#### In silico modelling of protein:protein interactions using AlphaFold-Multimer

To predict the interaction between TIM-1 and the N-terminus of MCM2, AlphaFold-Multimer (36, 70) was used to model MCM-2 residues 82-171, and TIM-1 residues 1947 (except replacing residues 521-834 with a GGSGGSGGSGGS linker).

To predict the interaction between the *C. elegans* GINS tetramer and the N-terminus of DNSN-1, AlphaFold-Multimer (36, 65, 67) was used to model full-length PSF-1, PSF-2, PSF-3 and SLD-5, plus DNSN-1 (residues 1-90). To predict the interaction between DONSON and GINS in humans, AlphaFold-Multimer (36, 65, 67) was used to model PSF1, PSF2, PSF3, SLD5 and two copies of DONSON, all full-length.

To predict the interaction between *C. elegans* DNSN-1 and the MCM-3 AAA+ domain, AlphaFold-Multimer (36, 65, 67) was used to model full-length MCM-3 and DNSN-1. To predict the interaction between DONSON and MCM3 in humans, AlphaFold-Multimer (36, 65, 67) was used to model MCM-3 and two copies of DNSN-1, all full-length.

To predict the existence of a *C. elegans* DNSN-1 homodimer, AlphaFold-Multimer (36, 65, 67) was used to model two copies of full-length DNSN-1. To predict the interaction between *C. elegans* DNSN-1 and MUS-101, AlphaFold-Multimer (36, 65, 67) was used to model two copies of full-length DNSN-1 plus MUS-101 residues 434-561 encompassing the MUS-101 BRCT3 domain. To predict the interaction between human DONSON and TOPBP1, AlphaFold-Multimer (36, 65, 67) was used to model two copies of full-length DONSON with full-length TOPBP1.

#### Statistics and Reproducibility

GraphPad Prism (GraphPad Software) was used to perform statistical analysis. For the EdU cell proliferation assay in Figure 2D, and the DNA combing assay in Figure 2F, data were analyzed via a Kruskal–Wallis test followed by Dunn’s. In Figure 8C, data were analyzed via a paired two-tailed t-test.

In microscopy experiments involving worms treated with RNAi, at least five embryos were examined for each treatment and found to behave similarly to each other, unless stated otherwise in the text.

## Supplementary Material

Supplementary Materials

## Data Availability

Cryo-EM density maps of the DNSN-1_CMG_TIM-1_TIPN-1 complex on a model replication fork have been deposited in the Electron Microscopy Data Bank (EMDB) under the following accession numbers: EMD-17204 (full complex); EMD-17198 (full complex prior to enrichment of particles containing TIM-1_TIPN-1); EMD-17199 (Multi-body refinement [MBR]: DNSN-1 homodimer); EMD-17200 (MBR: GINS_CDC-45_DNSN-1 N-terminus); EMD-17201 (MBR: MCM-2-7 AAA+_ssDNA); EMD-17202 (MBR: MCM-2-7 NTDs); EMD-17203 (MBR: TIM-1_TIPN-1_MCM-2-7 NTDs_dsDNA). Atomic coordinates for the DNSN-1_CMG_TIM-1_TIPN-1 complex on a model replication fork have been deposited in the Protein Data Bank (PDB) under the accession code 8OUW. Mass spectrometry proteomics data have been deposited to the ProteomeXchange Consortium via the PRIDE partner repository (78) with the dataset identifier PXD044240 and 10.6019/PXD044240. Otherwise, all data are available in the manuscript or the supplementary materials. Materials generated in this study are listed in Table S5 and are available from MRC PPU Reagents and Services (https://mrcppureagents.dundee.ac.uk) or upon request from the corresponding authors.

## One sentence summary

*C. elegans* DNSN-1 works with the BRCT protein MUS-101/TOPBP1 to build the CMG helicase during DNA replication initiation.
