# Supplementary material for: DNSN-1 recruits GINS for CMG helicase assembly during DNA replication initiation in C. elegans
Source: Science. Author manuscript; Available in PMC 2023 Sep 23. (PMC7615117; doi:10.1126/science.adi4932)
Supplement: Supplementary Materials [file EMS187426-supplement-Supplementary_Materials.pdf]

Science
AAAS

Supplementary Materials for

DNSN-1 recruits GINS for CMG helicase assembly during DNA replication initiation in *C. elegans*

Yisui Xia, Remi Sonneville, Michael Jenkyn-Bedford, Liqin Ji, Constance Alabert, Ye Hong, Joseph T.P. Yeeles, and Karim P.M. Labib

Correspondence to: kpmlabib@dundee.ac.uk; jyeeles@mrc-lmb.cam.ac.uk; hongye@sdu.edu.cn

This PDF file includes:

Figs. S1 to S18
Tables S1 to S5
Captions for Data S1 to S3

Other Supplementary Materials for this manuscript include the following:

Data S1 to S3

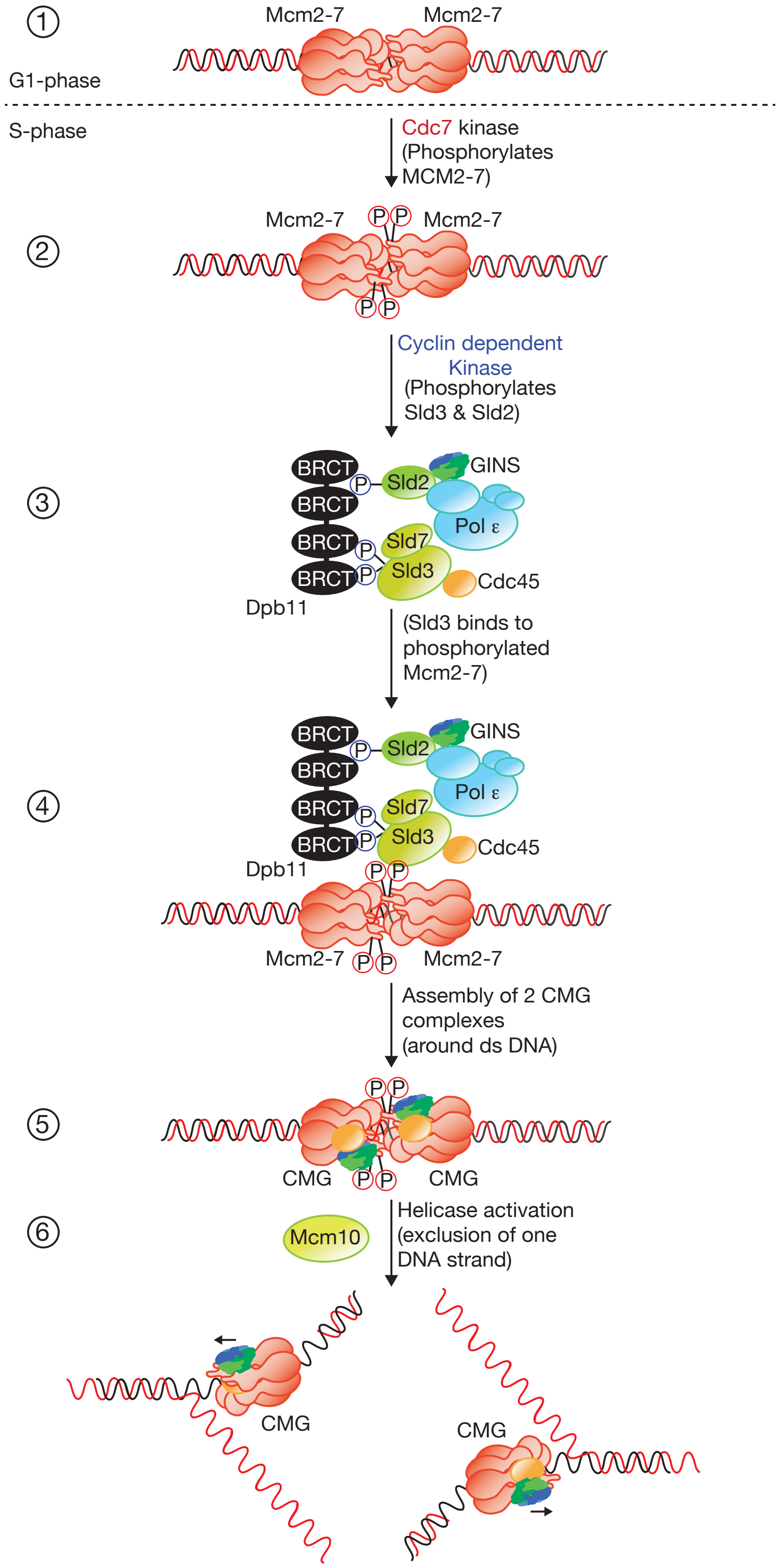

Fig. S1.

Assembly and activation of the CMG helicase during DNA replication initiation in budding yeast.

Model for assembly and activation of the yeast CMG helicase – see text for details.

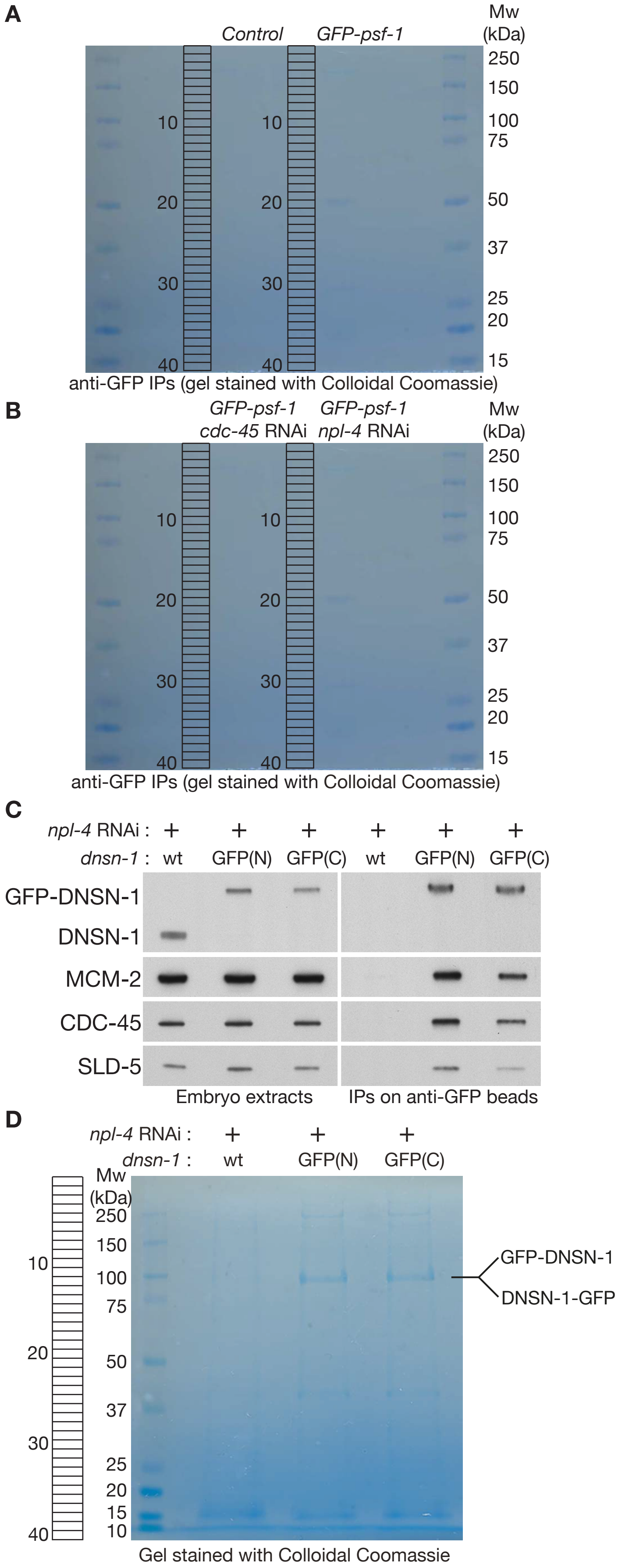

Fig. S2.

Preparation of samples for mass spectrometry analysis of factors associating with the worm replisome and DNSN-1. (**A-B**) The remainder of each sample from the experiment in Figure 1A was resolved in a 4-12% gradient gel that was then stained with colloidal Coomassie blue. Each lane was cut into 40 bands as indicated before analysis by mass spectrometry. (**C**) Control worms (N2), *GFP-dnsn-1* (KAL213) and *dnsn-1-GFP* (KAL214) were grown as above on bacteria expressing *npl-4* RNAi. Embryonic cell extracts were then incubated with anti-GFP beads and the indicated factors were monitored by immunoblotting. (**D**) The remainder of the samples from (C) were processed for mass spectrometry as above.

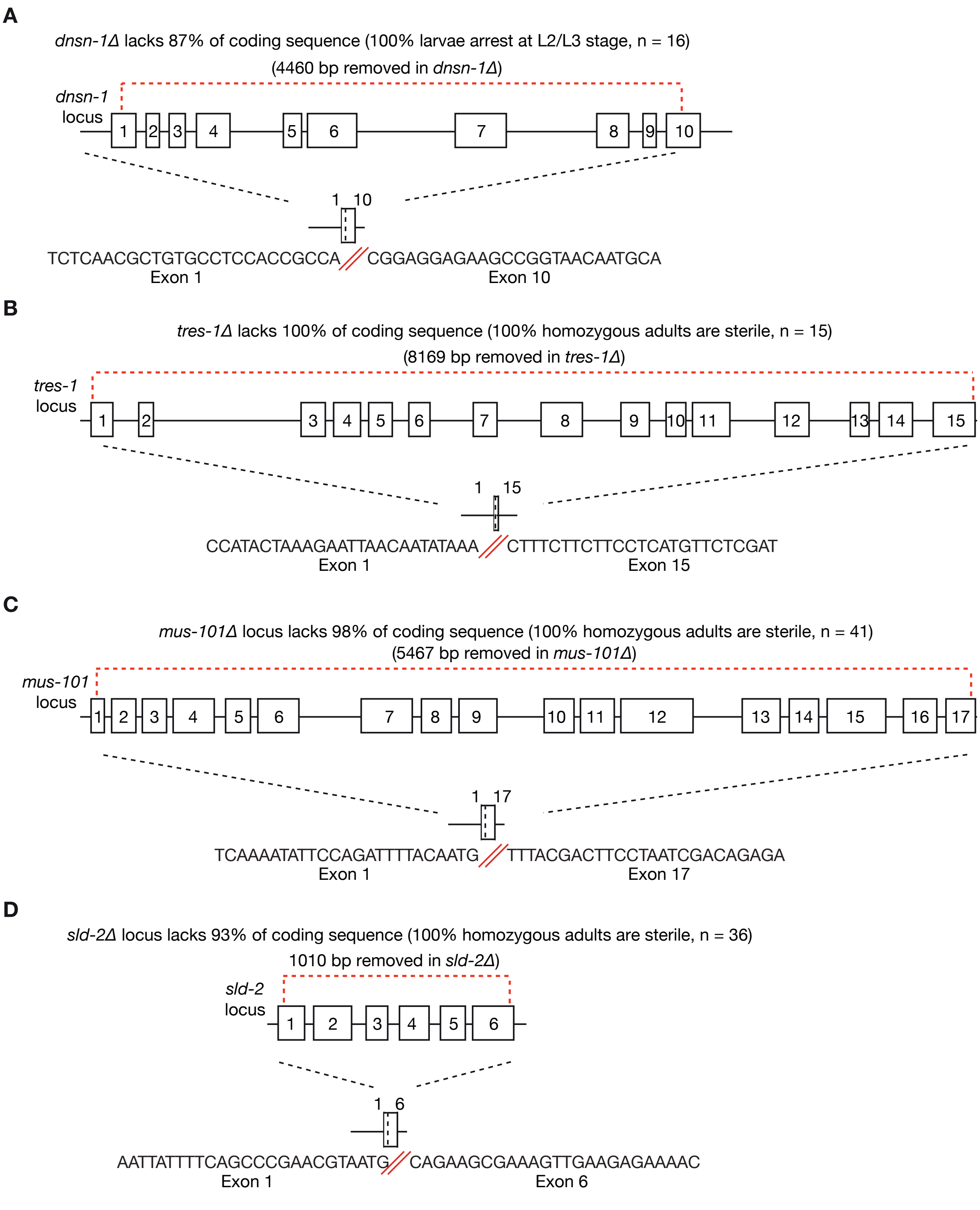

Fig. S3.
Deletion of *dnsn-1*, *mus-101*, *tres-1* and *sld-2* by CRISPR-Cas9.
(**A**) Illustration of the *dnsn-1* locus in the *C. elegans* genome, together with details of the residues removed in *dnsn-1*Δ and the associated phenotype. (**B-D**) Similar details for *mus-101*Δ, *tres-1*Δ and *sld-2*Δ.

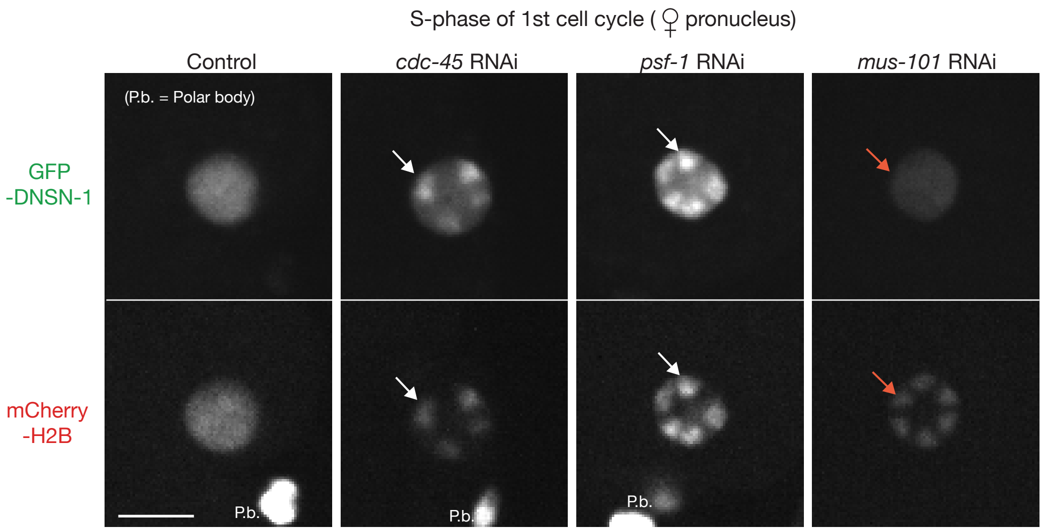

Fig. S4.

Association of DNSN-1 with pre-initiation complexes requires MUS-101

Images are shown of the female pronucleus during early S phase of the first embryonic cell cycle, from worms expressing GFP-DNSN-1 plus mCherry-Histone H2B (KAL267) and fed with bacteria expressing the indicated RNAi. White arrows correspond to chromatin-bound DNSN-1. Red arrows indicate absence of DNSN-1 from condensed chromosomes upon depletion of MUS-101. Scalebars correspond to 5µm. Note that difference in brightness between images can reflect the variable depth at which the female pronucleus is located within the embryo.

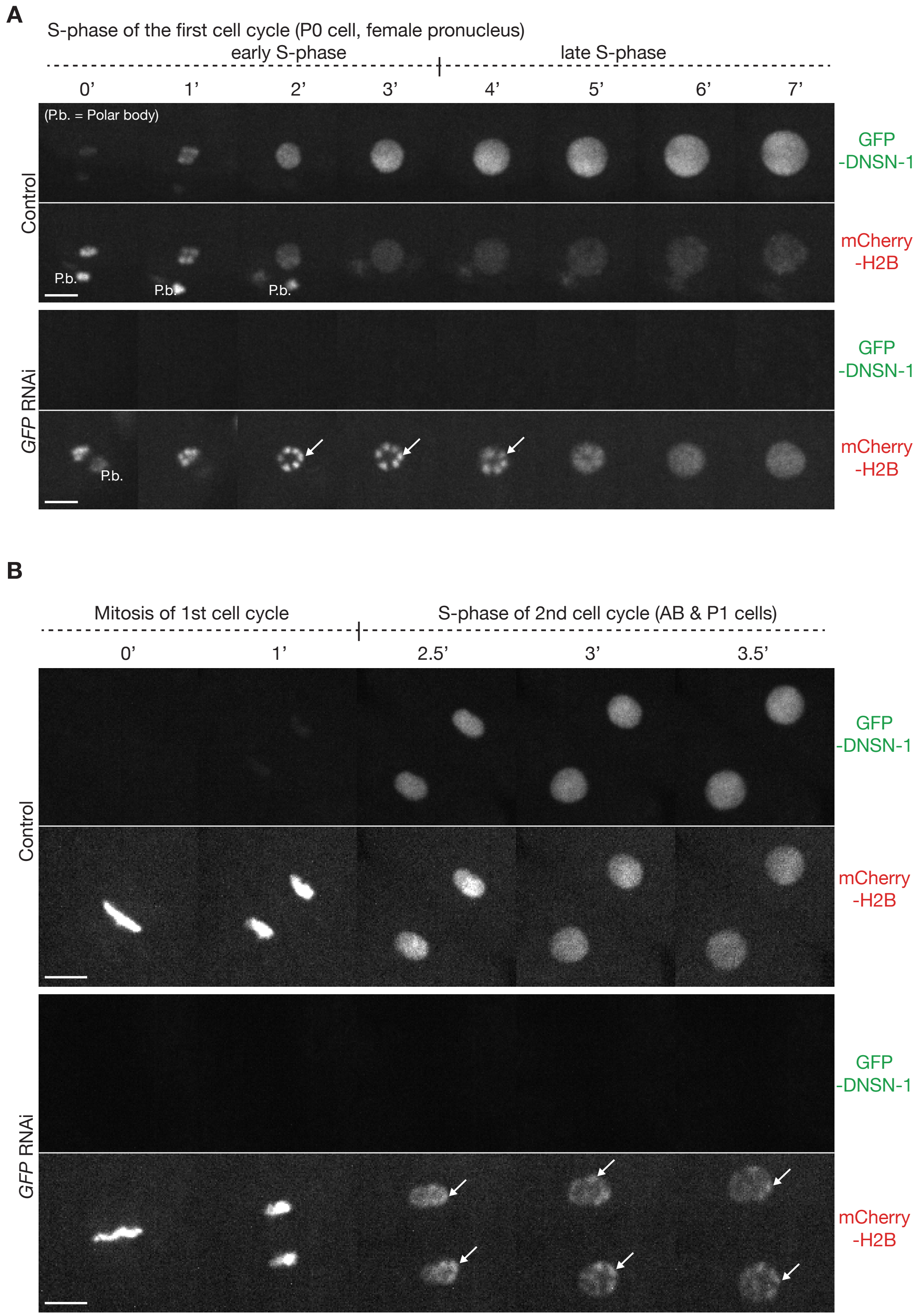

Fig. S5.

DNSN-1 is required for rapid chromatin decondensation upon entry into S phase.

Embryos expressing GFP-DNSN-1 and mCherry-Histone H2B (KAL267) were fed on bacteria expressing GFP RNAi, or containing empty vector as negative control, before analysis by video microscopy. The images show progression through S-phase of the first cell cycle (**A**), or through the first embryonic mitosis into S phase of the second cell cycle (**B**). Arrows indicate the persistence of condensed chromosomes upon depletion of GFP-DNSN-1. Scalebars correspond to 5μm.

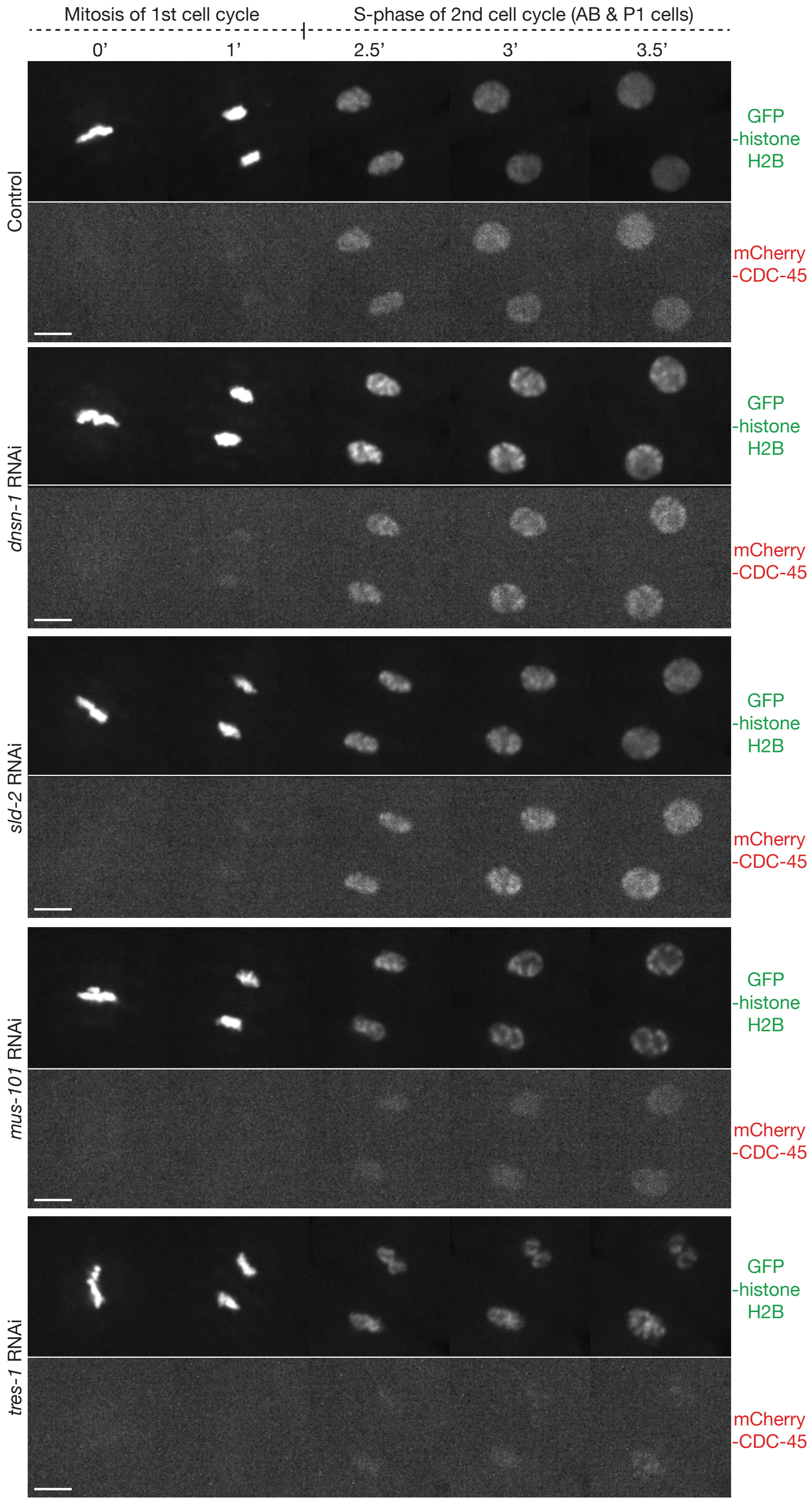

Fig. S6.
DNSN-1 and SLD-2 are dispensable for recruitment of CDC-45 to chromatin during S-phase, unlike TRES-1 and MUS-101.
Embryos expressing GFP-Histone H2B and mCherry-CDC-45 (KAL265) were fed on bacteria expressing the indicated RNAi, or containing empty vector as negative control, before analysis by video microscopy. The images show progression through the first embryonic mitosis into S phase of the second cell cycle. Scalebars correspond to 5µm.

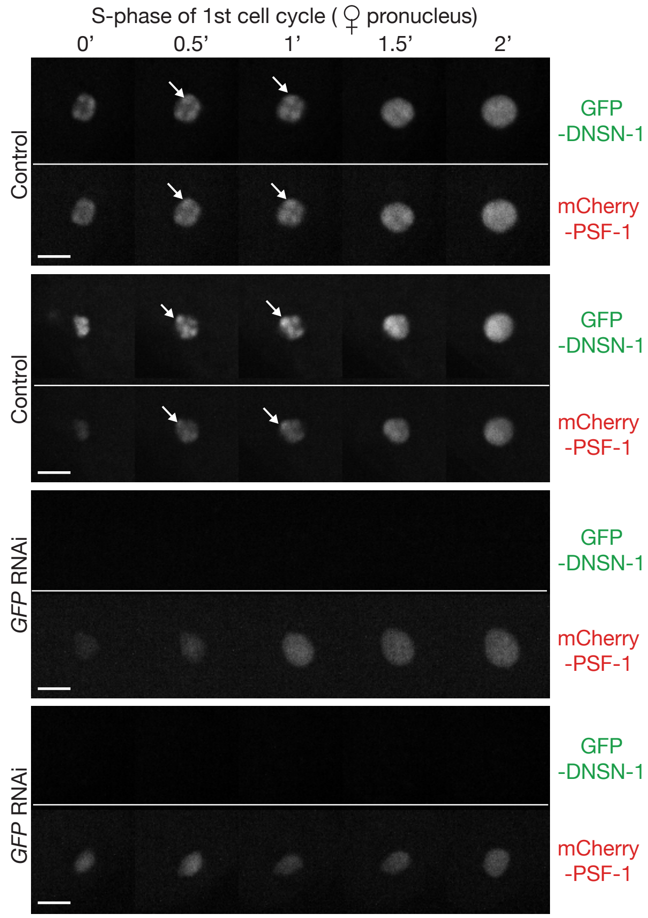

Fig. S7.

DNSN-1 is required for chromatin association of GINS in the C. elegans early embryo.

Additional examples are shown from the experiment in Figure 4E. Arrows denote chromatin association of mCherry-PSF-1 and GFP-DNSN-1 during early S-phase in control embryos.

Scalebars correspond to 5µm.

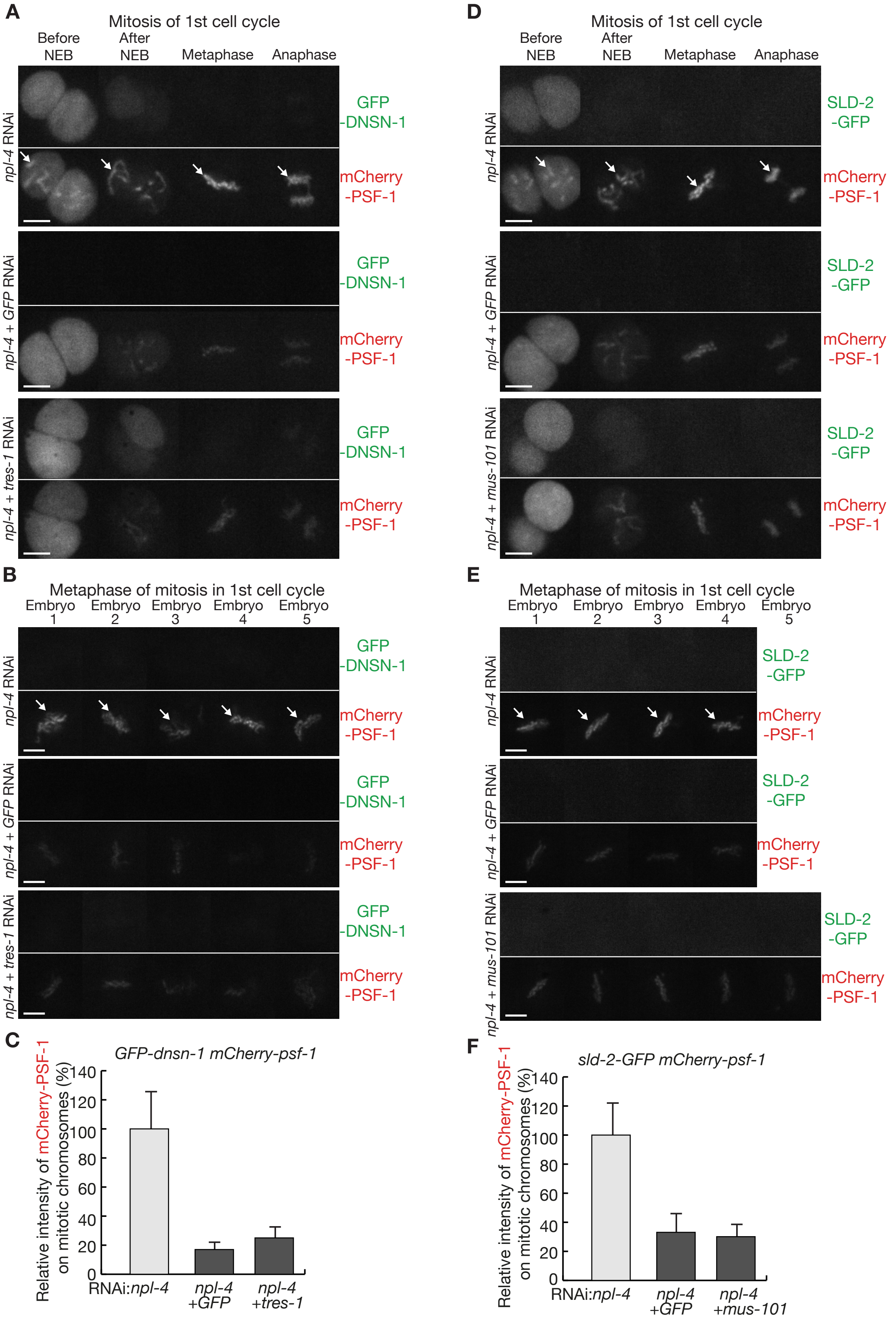

Fig. S8.

DNSN-1, SLD-2, MUS-101 and TRES-1 are all required for CMG assembly in the C. elegans early embryo.

(**A**) Worms expressing GFP-DNSN-1 and mCherry-PSF-1 (KAL269) were fed on bacteria expressing the indicated RNAi, before analysis by video microscopy during passage through mitosis of the first embryonic cell cycle. Arrows denote chromatin-bound mCherry-PSF-1 in embryos treated with single RNAi to *npl-4*. (**B**) Images of five such embryos during metaphase. (**C**) Quantitation of chromatin-bound mCherry-PSF-1 during metaphase, showing mean and standard deviation for five embryos. The data were normalized to the control treated with *npl-4* RNAi alone. (**D-F**) Equivalent experiment with embryos expressing GFP-SLD-2 and mCherry-PSF-1 (KAL272). Note that the mitotic spindle places the chromosomes in he center of the cell, always at the same position, leading to very similar brightness of the signal. This facilitates accurate quantification of chromatin-bound proteins such as mCherry-PSF-1. Scalebars correspond to 5μm.

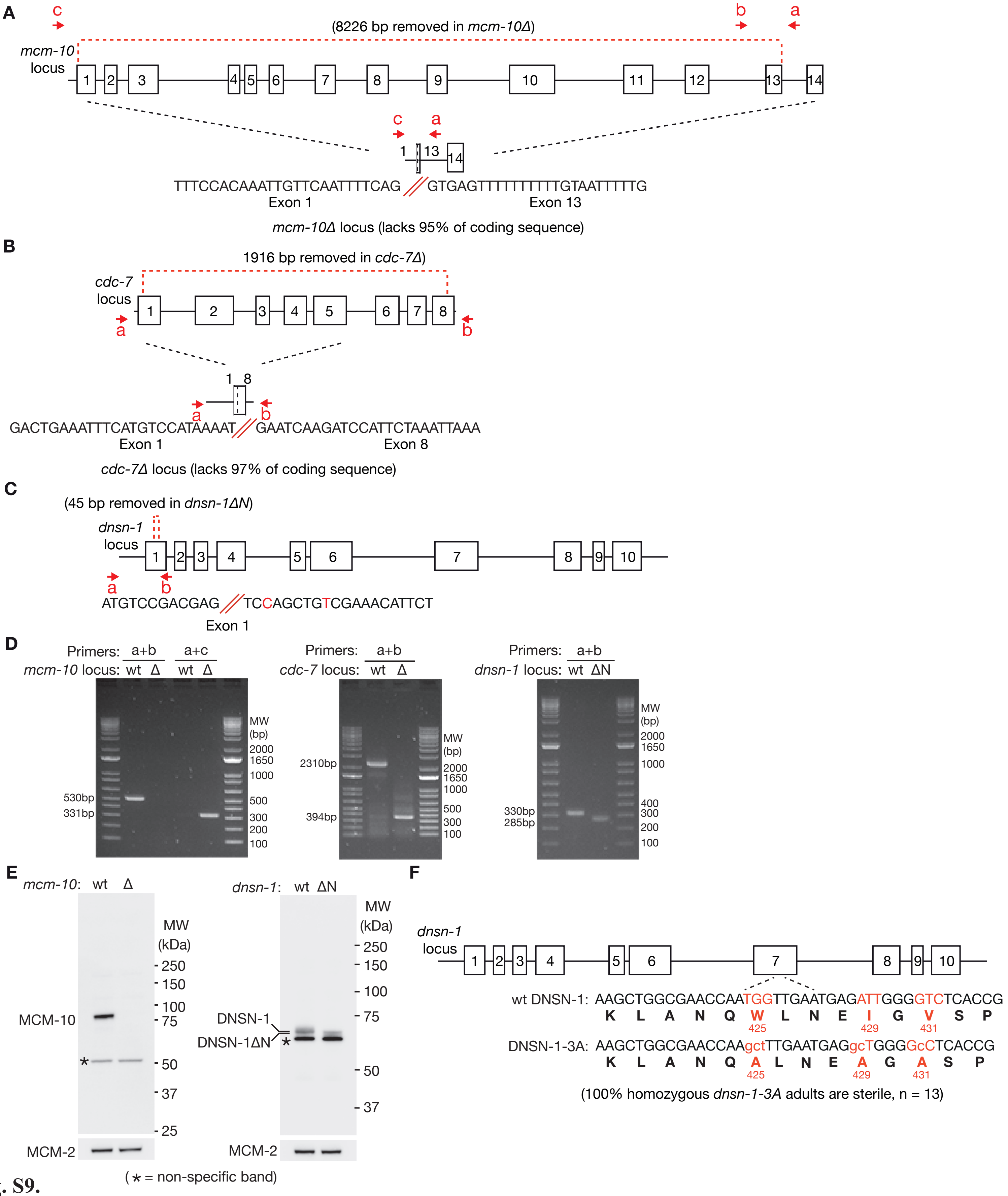

Fig. S9.

Generation and characterization of *mcm-10*Δ, *cdc-7*Δ, *dnsn-1*Δ*N* and *dnsn-1-3A*.
(**A**) Illustration of the *mcm-10* locus in the *C. elegans* genome, together with details of the residues removed in *mcm-10*Δ. The location of oligonucleotides used to monitor the locus by PCR analysis is indicated (a-c). (**B**) Equivalent details for the *cdc-7* locus and *cdc-7*Δ allele. (**C**) Analogous representation of the *dnsn-1* locus and *dnsn-1*Δ*N* allele. (**D**) PCR analysis of genomic DNA from *mcm-10*Δ, *cdc-7*Δ and *dnsn-1*Δ*N* worms. (**E**) Analysis of *mcm-10*Δ and *dnsn-1*Δ*N* by immunoblotting of embryonic extracts with the indicated antibodies. (**F**) Illustration of the mutations within exon 7 of *dnsn-1* that were made to create the *dnsn-1-3A* allele, corresponding to mutations in the interface between DNSN-1 and the AAA+ domain of MCM-3 (see Figure 7C (iii)).

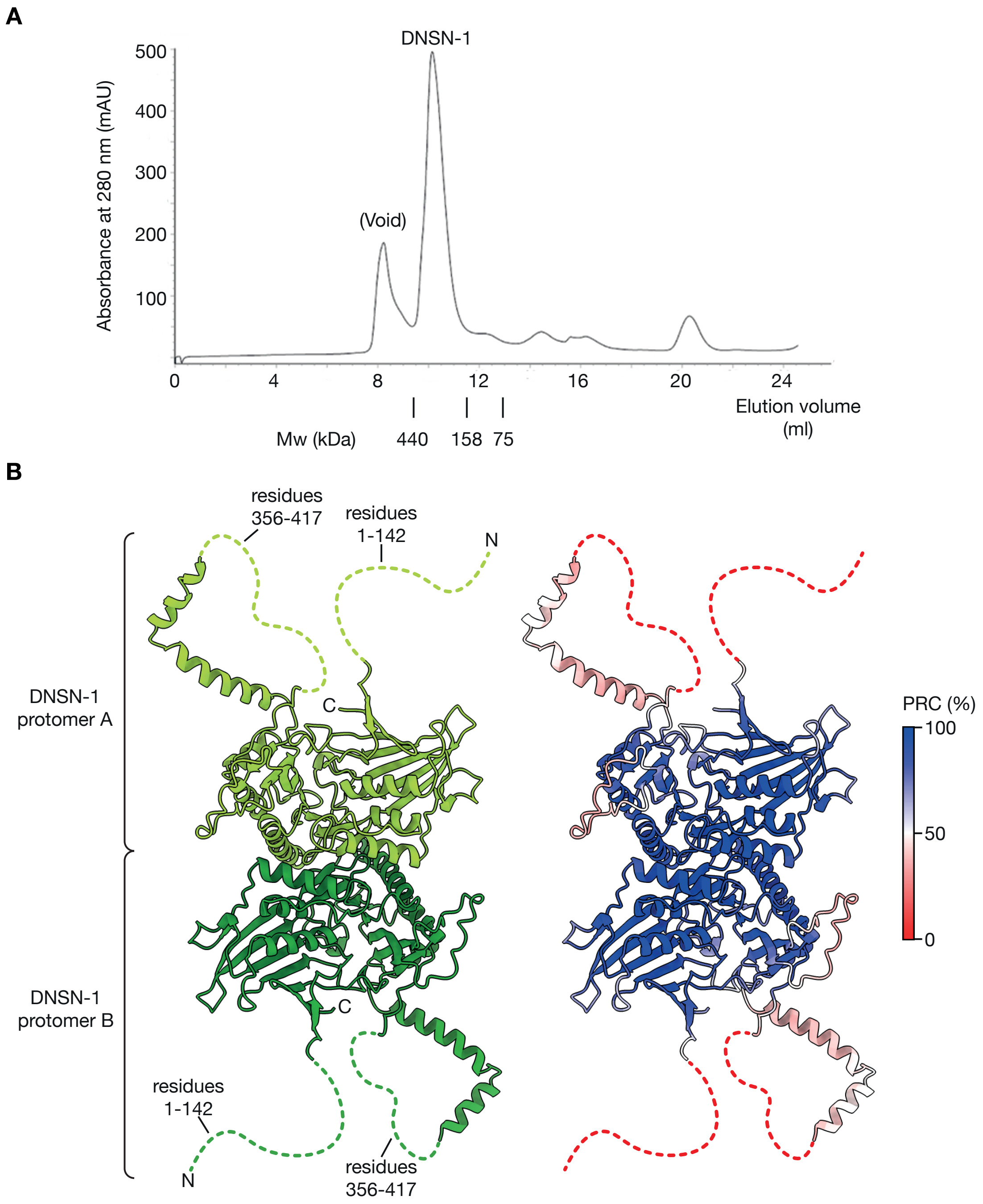

Fig. S10.
DNSN-1 is a homodimer.
(**A**) Migration of recombinant DNSN-1 through a size-exclusion chromatography column was compared with the indicated marker proteins. (**B**) AlphaFold-Multimer prediction of full-length DNSN-1 dimer colored by subunit (left) and by 'Per Residue Confidence' or PRC (right). For clarity, long disordered loops are illustrated as dashed lines. Amino- (N) and carboxy- (C) termini are labelled.

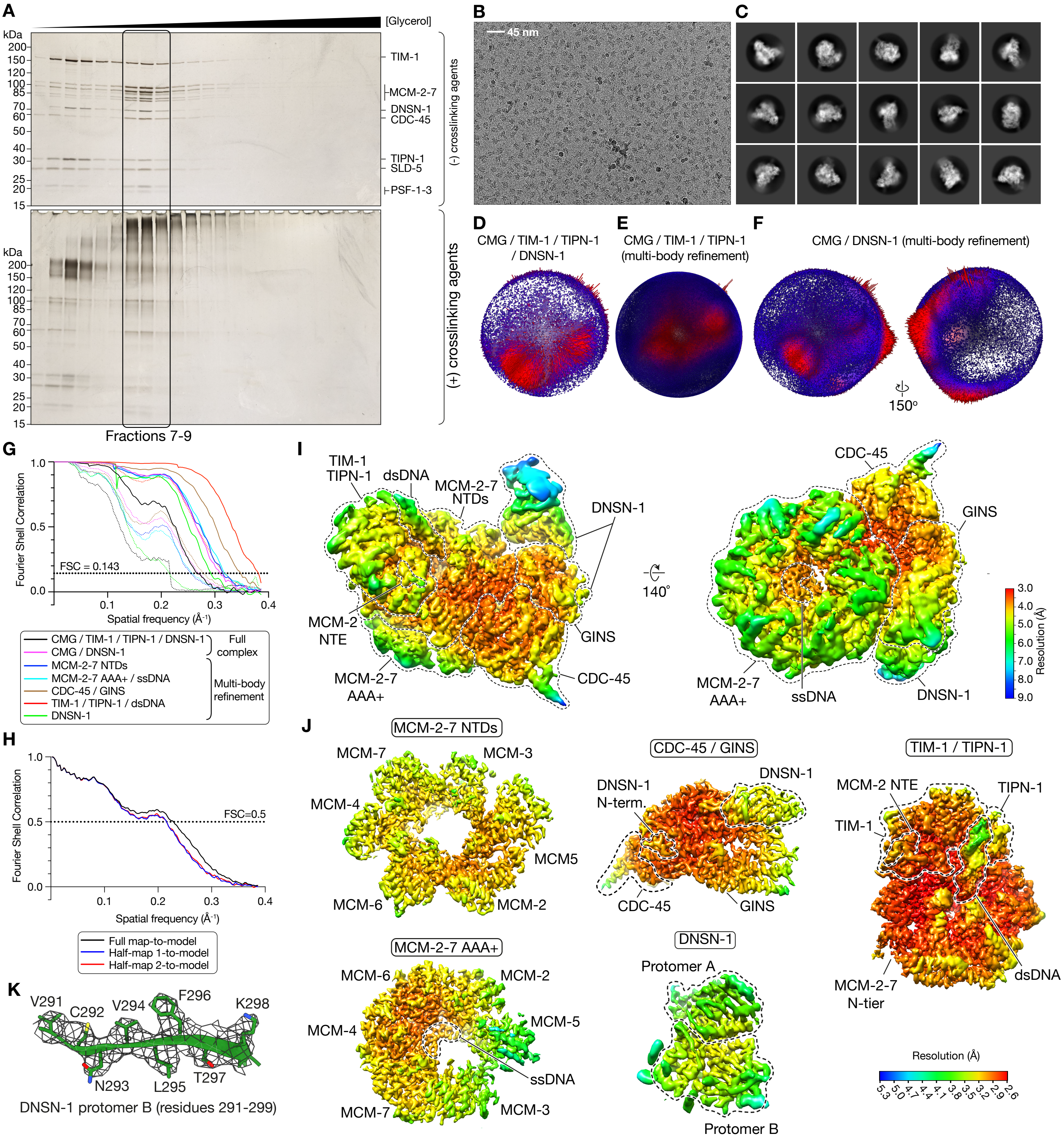

Fig. S11. Cryo-EM sample preparation and data processing.
(**A**) A complex of CMG, DNSN-1, TIM-1, TIPN-1 and fork DNA was assembled and applied to a 10 - 30% glycerol gradient, as in Figure 6C, but in the absence or presence of a gradient of cross-linking agents (0 - 0.22% glutaraldehyde plus 0 - 2 mM BS3) as indicated. Fraction 8 of the gradient without crosslinker is presented in Figure 6C and Fractions 7 – 9 of the gradient with crosslinker were pooled for cryo-EM sample preparation. (**B**) Representative cryo-EM micrograph. (**C**) Representative cryo-EM 2D class averages (box width = 41 nm). (**D-F**) Angular distribution of particles contributing to the reconstructions of the cryo-EM density maps representing the full CMG/TIM-1/TIPN-1/DNSN-1 complex (D), the CMG/TIM-1/TIPN-1 complex used for multi-body refinement or MBR (E) and the CMG/DNSN-1 complex used for MBR (F, also refer to Figure S12). The orientation shown in (D), (E), and (F, left) is equivalent to that shown in panel (I, top). (**G**) Cryo-EM density map Fourier shell correlation (FSC) curves. Masked and unmasked FSC curves are displayed as solid and dotted lines respectively. (**H**) Map-to-model FSC curve for the cryo-EM density map representing the complete CMG/TIM-1/TIPN-1/DNSN-1 complex on DNA. (**I-J**) Cryo-EM density maps colored by local resolution representing the complete CMG/TIM-1/TIPN-1/DNSN-1 complex on DNA that was used for model refinement (I) or the maps derived from multi-body refinement that were used for model building (J, also refer to Figure S12). (**K**) Cryo-EM density (mesh representation) for the indicated region of the folded domain of DNSN-1 protomer B, which contacts GINS and the MCM-3 NTD (included to illustrate the quality of the cryo-EM density).

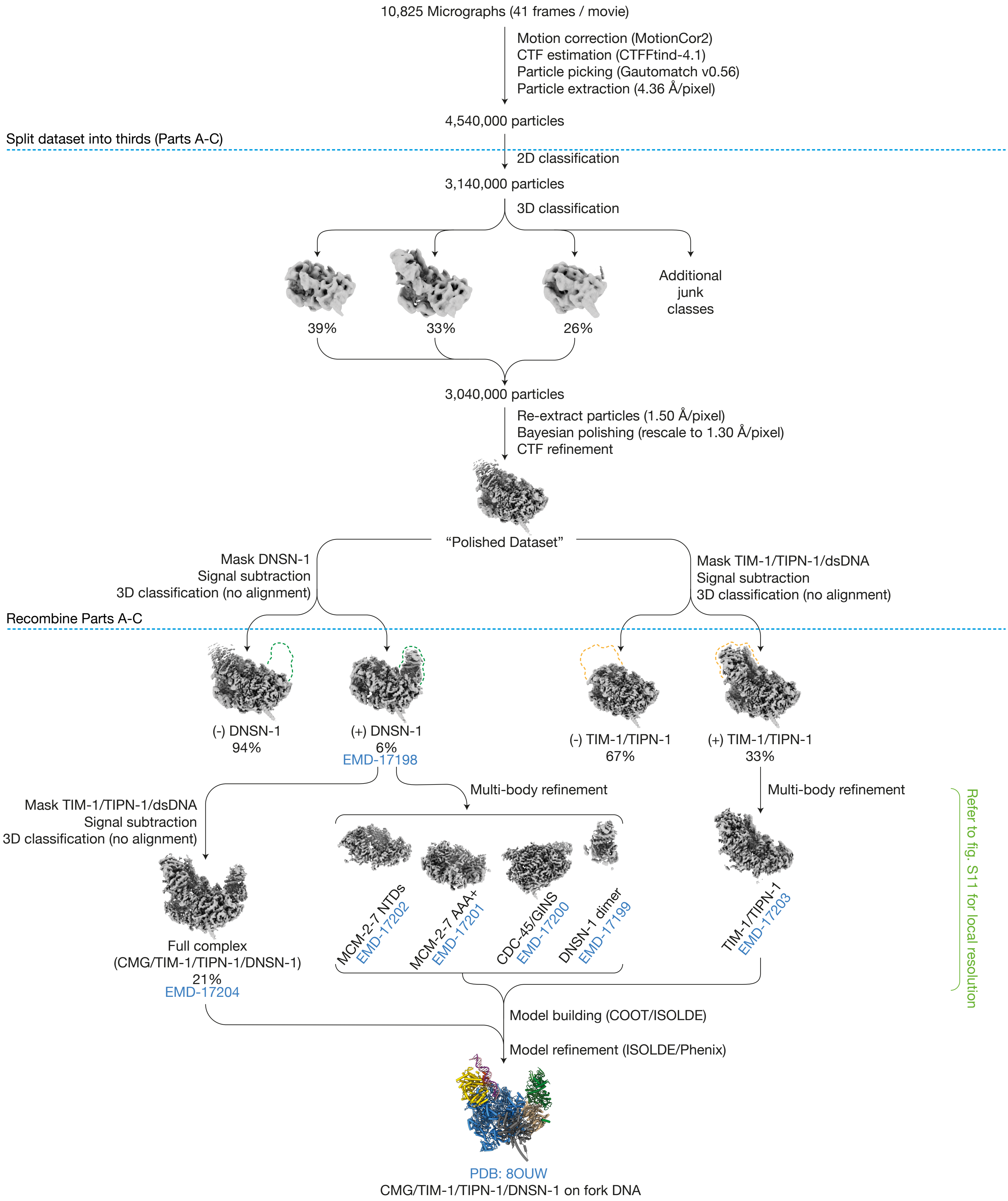

Fig. S12.

Pipeline for cryo-EM data processing.

Yellow and green dashed outlines highlight the positions of TIM-1/TIPN-1 and the DNSN-1 homodimer, respectively. Accession codes are shown for maps deposited in the Electron Microscopy Data Bank (EMDB) and for the atomic model deposited in the Protein Data Bank (PDB).

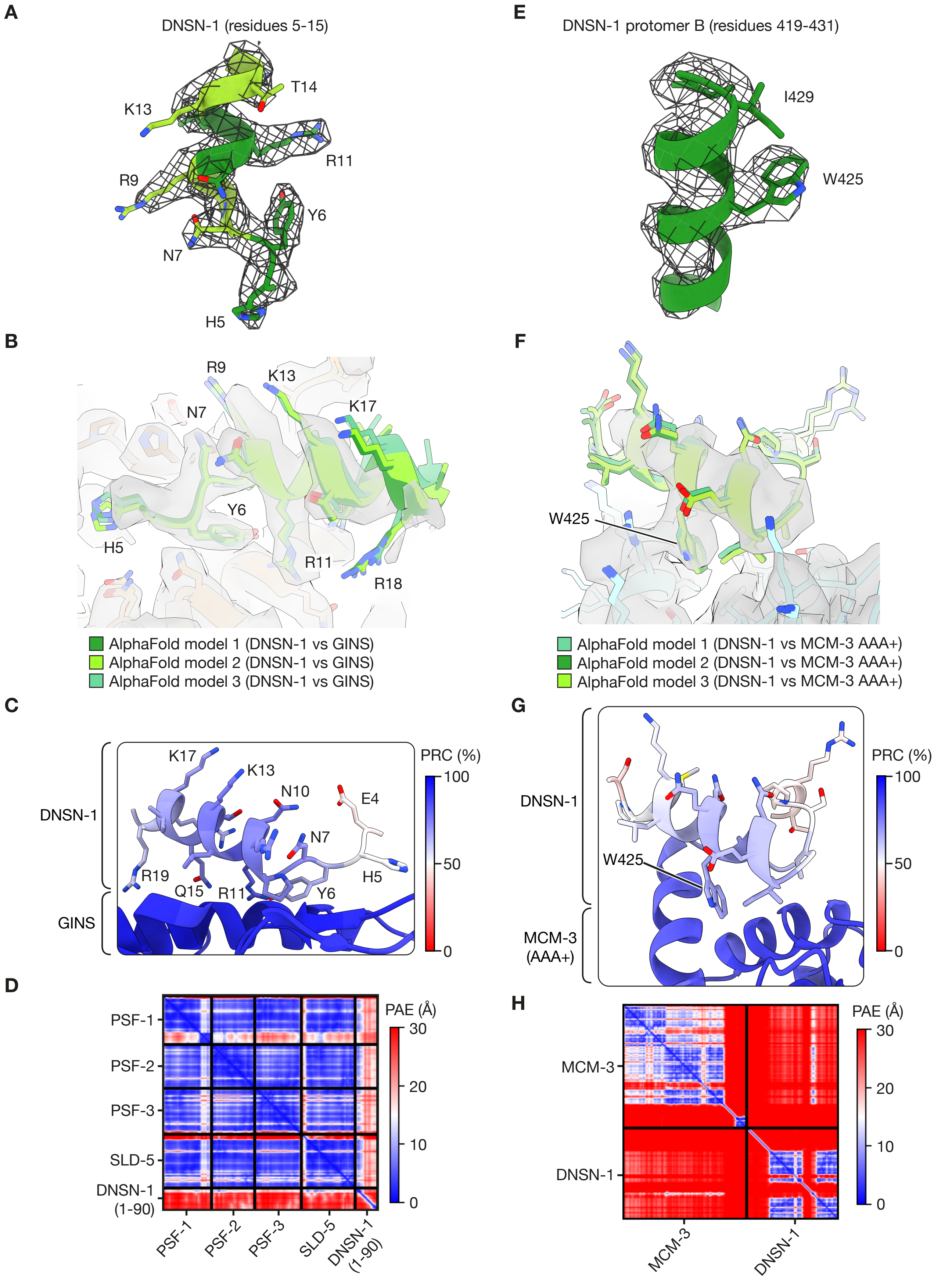

Fig. S13.

Supporting figure for the cryo-EM reconstruction of the CMG/TIM-1/TIPN-1/DNSN-1 complex, related to modelling of DNSN-1.

(**A**) Cryo-EM density (mesh representation) for residues 5-15 of DNSN-1. (**B**) AlphaFold-Multimer was used to predict the interaction between residues 4-19 of DNSN-1 and GINS. The top-three ranked predictions were then fitted as rigid-bodies to the cryo-EM density (DNSN-1 is colored shades of green with annotation of selected residues; GINS is colored shades of brown). (**C**) The top-ranked prediction from (B), colored by 'Per Residue Confidence' (PRC). (**D**) Predicted Aligned Error (PAE) plots for the top-ranked prediction in (B). (**E**) Cryo-EM density (mesh representation) for residues 419-431 of DNSN-1 protomer B. (**F**-**H**) Analogous analysis to that in (B-D) for the interaction between residues 417-435 of DNSN-1 protomer B and the MCM-3 AAA+ domain (colored in blue in panel (F)). See Materials and Methods for further details.

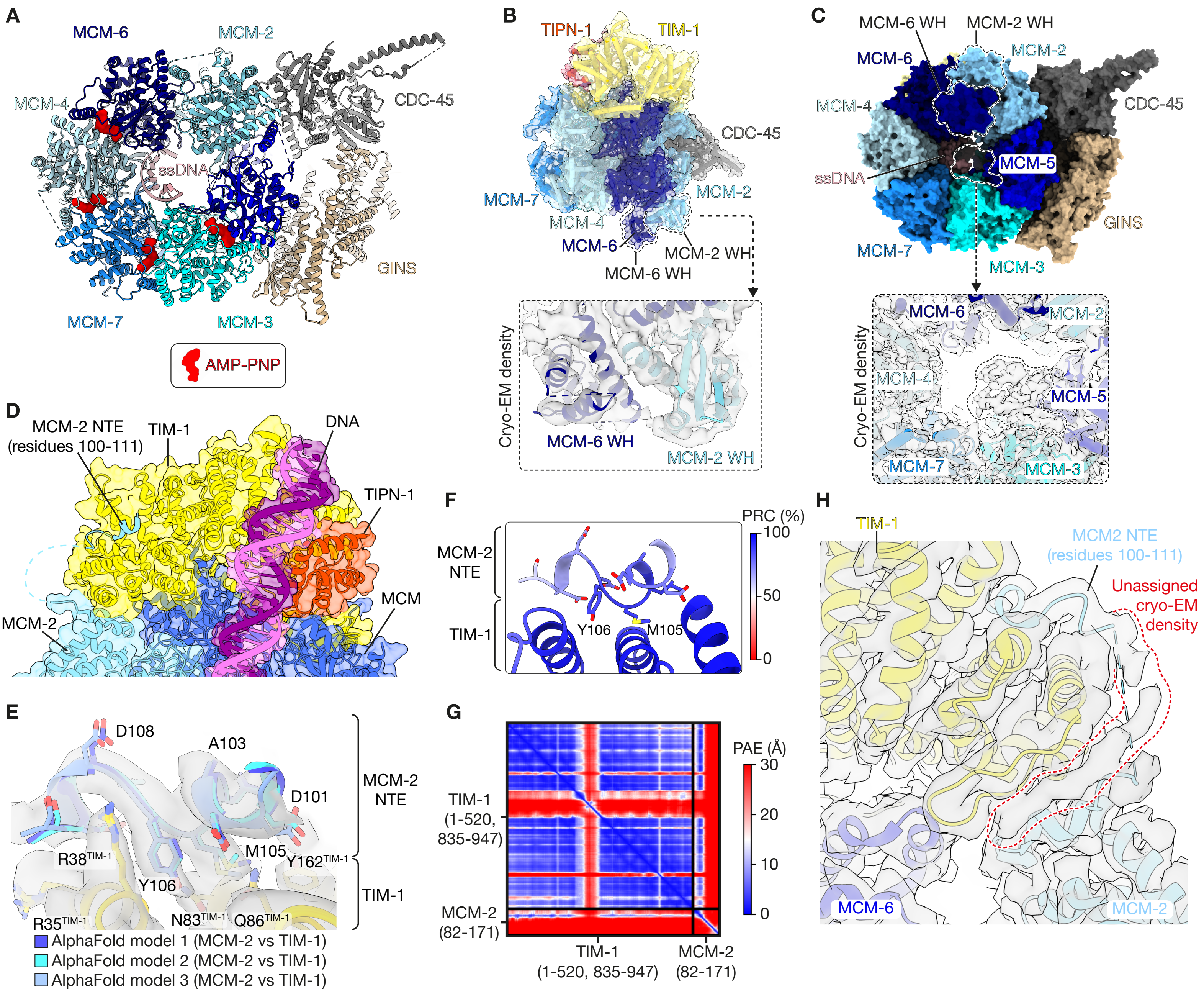

Fig. S14.

Supporting figure for the cryo-EM reconstruction of the CMG/TIM-1/TIPN-1/DNSN-1 complex, related to modelling of MCM-2-7 subunits.

(**A**) Atomic model illustrating AMP-PNP occupancy of the MCM-2-7 AAA+ domains. (**B**) Atomic model, rendered as a cartoon within transparent surface, illustrating the position of the C-terminal winged-helix (WH) domains of MCM-2 and MCM-6. The inset shows the observed cryo-EM density. (**C**) Atomic model rendered as a surface to illustrate the position of a third, unassigned MCM-2-7 WH domain (dashed outline). The inset again shows the observed cryo-EM density. (**D**) Atomic model illustrating the position of the MCM-2 amino-terminal extension (NTE) interacting with the amino-terminus of TIM-1. (E-G) AlphaFold-Multimer predictions for the MCM-2 NTE:TIM-1 interaction (refer to Materials and Methods for details): (**E**) top-three ranked predictions fitted as rigid-bodies to cryo-EM density; (**F**) Top-ranked prediction colored by per residue confidence (PRC); (**G**) Predicted Aligned Error (PAE) plot for the top-ranked prediction; modelled residues are indicated. (**H**) Unassigned cryo-EM density (red dashed outline) observed adjacent to TIM-1 and MCM-2. Cryo-EM density represented as transparent grey surface.

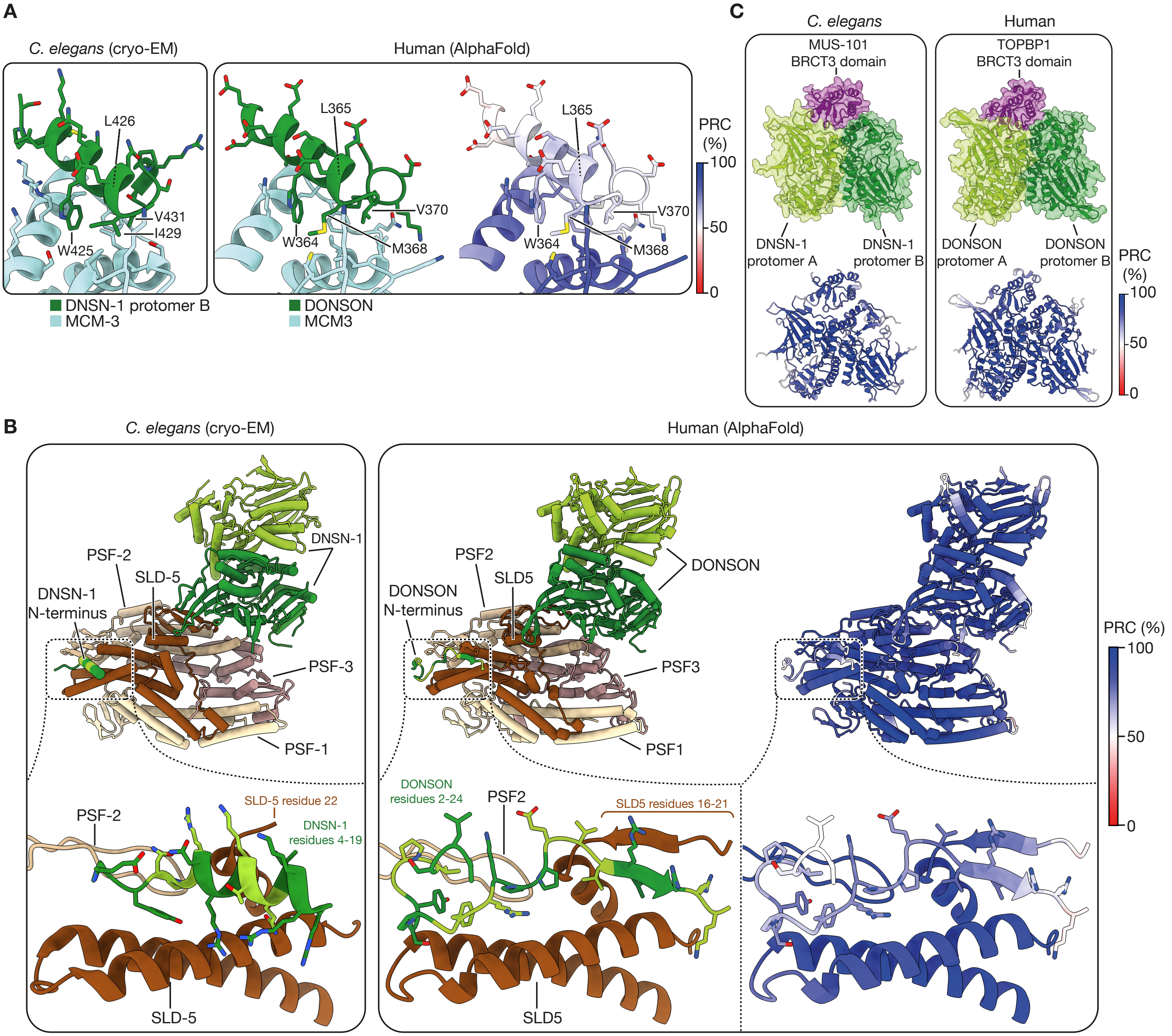

Fig. S15.

Conserved interactions of C. elegans DNSN-1 and human DONSON with CMG and MUS-101/TOPBP1.

For details regarding sequences used for AlphaFold-Multimer predictions, refer to Materials and Methods. (**A**) Comparison of the observed interaction between *C. elegans* DNSN-1 protomer B and the MCM-3 AAA+ domain via cryoEM, with the AlphaFold-Multimer prediction for the interaction of human DONSON with MCM3. For the AlphaFold prediction, proteins are colored by subunit (left) and Per Residue Confidence or PRC (right). (**B**) Analogous comparison of the observed interaction between *C. elegans* DNSN-1 and GINS via cryoEM, with the predicted interaction of human DONSON with GINS by AlphaFold-Multimer. For the AlphaFold prediction, proteins are colored by subunit (left) and Per Residue Confidence or PRC (right). The panels show an overview (top) and a view focusing on the DNSN-1 / DONSON amino-terminus (bottom). Note that the amino terminus of *C. elegans* DNSN-1 forms an α-helix, whereas that of human DONSON adopts a more extended conformation and forms an anti-parallel β-sheet with SLD5 residues 18-21. (**C**) Comparison of the interactions predicted by AlphaFold -Multimer for the *C. elegans* DNSN-1 dimer and the MUS-101 BRCT3 domain (left), or the human DONSON dimer with BRCT3 of TOPBP1 (right). Models are colored by subunit (top) and Per Residue Confidence or PRC (bottom). In both species, BRCT3 simultaneously contacts both protomers of DNSN-1/DONSON at the homodimer interface.

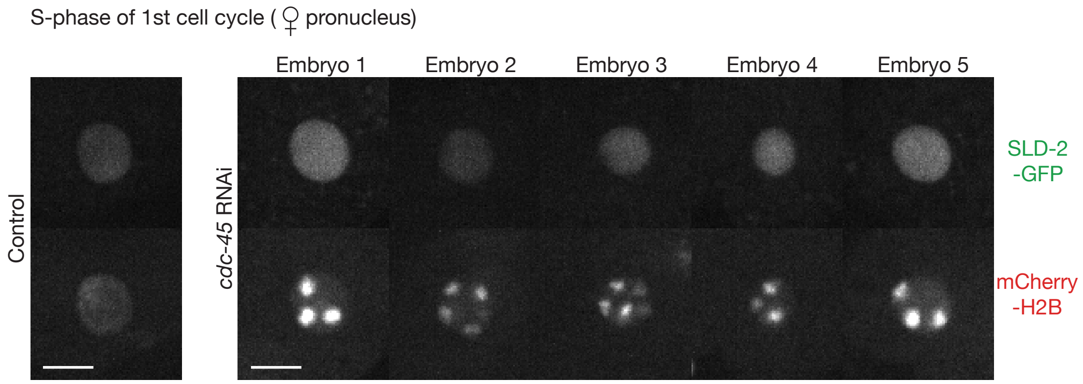

Fig. S16.
SLD-2 is not detected on pre-initiation complexes
Embryos expressing SLD-2-GFP and mCherry-Histone H2B (KAL270) were fed on bacteria expressing *cdc-45* RNAi or containing an empty vector (control), before analysis of entry into S phase of the first embryonic cell cycle (female pronucleus) by spinning disk confocal microscopy. Scalebar corresponds to 5µm.
Note that difference in brightness between images can reflect the variable depth at which the female pronucleus is located within the embryo.

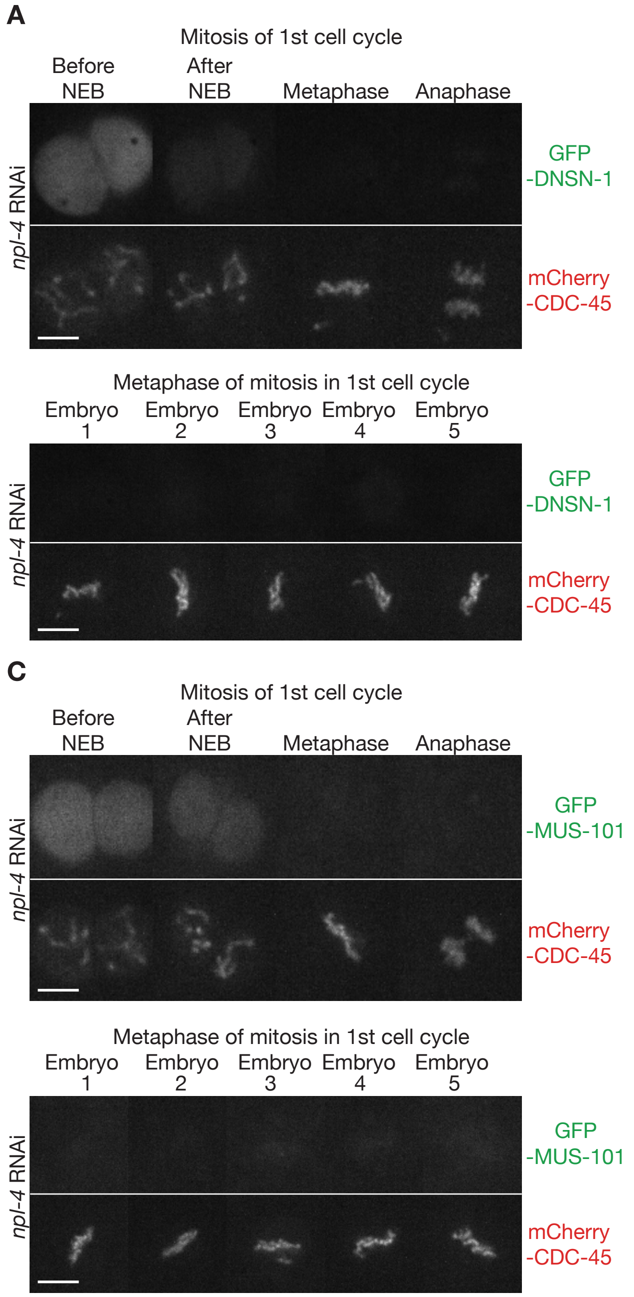

Fig. S17.

Unlike the CMG helicase, the initiation factors DNSN-1, TRES-1, MUS-101 and SLD-2 are not detected on mitotic chromosomes after depletion of NPL-4.

(**A**) Embryos expressing GFP-DNSN-1 and mCherry-CDC-45 (KAL268) were treated with *npl-4* RNAi and analyzed by video microscopy, upon entry into mitosis of the first embryonic cell cycle. The lower panel shows 5 such embryos during metaphase. (**B**-**D**) Similar experiments with *tres-1-GFP mCherry-cdc-45* embryos (KAL274), *GFP-mus-101 mCherry-cdc-45* (KAL277) and *sld-2-GFP mCherry-cdc-45* (KAL271). Scalebars correspond to 5µm.

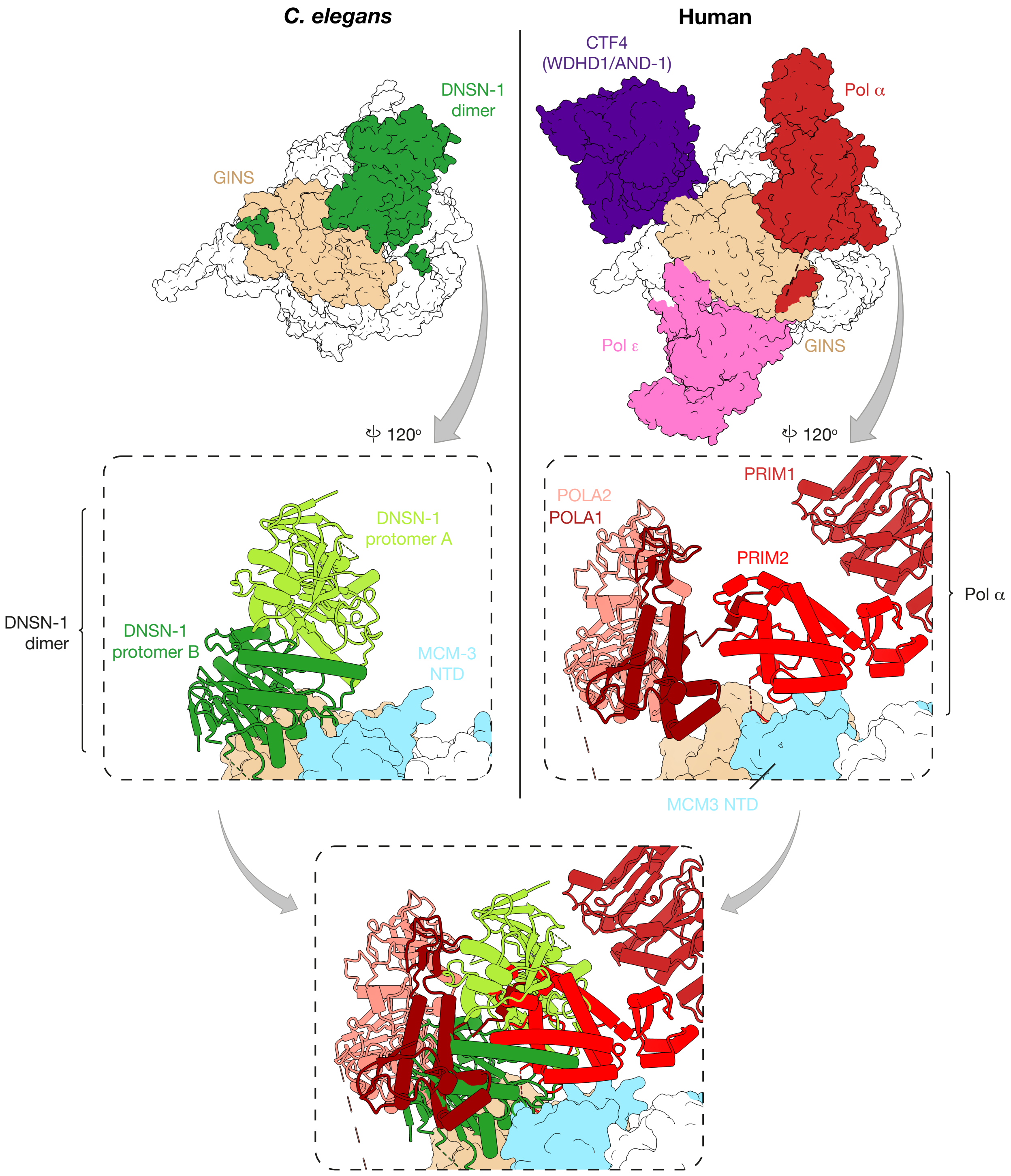

Fig. S18.

DONSON and DNA polymerase α bind to overlapping sites on the replisome.

Top: Comparison of the cryo-EM structure of *C. elegans* DNSN-1 dimer bound to the CMG helicase complex (this study) with prior cryo-EM structures of human replisome subcomplexes. The human complex was produced from a composite of the following data: the complex of CMG helicase-Pol ε-CTF4-TIM-TIPN-DNA, PDB: 7PFO (*79*) and the complex of CMG helicase-Pol α-CTF4-TIM-TIPN-DNA, PDB: 8B9D (*55*). Middle: expanded and rotated views for the interfaces between DNSN-1 / Pol α with GINS and MCM-3. Individual subunits are colored shades of green (DNSN-1), red (Pol α) and blue (MCM-3). Bottom: superposition of the expanded views shown above, highlighting the clash that would occur between the observed positions of DNSN-1 and Pol α.

Table S1.
MS analysis showing that DNSN-1 and the initiation factors MUS-101 and TRES-1 co-purify with GFP-PSF-1 and worm replisome components.

Protein	Replisome sub-complex	Spectral counts (control)	Spectral counts (GFP-PSF-1)	Spectral counts (GFP-PSF-1) *cdc-45* RNAi	Spectral counts (GFP-PSF-1) *npl-4* RNAi
GFP-PSF-1 (54 kDa)	CMG (GINS)	174	2112	2017	3203
PSF-2 (20 kDa)	CMG (GINS)	43	596	353	703
PSF-3 (22 kDa)	CMG (GINS)	22	876	522	1038
SLD-5 (26 kDa)	CMG (GINS)	21	874	750	1239
CDC-45 (66 kDa)	CMG (CDC-45)	8	77	6	534
MCM-2 (99 kDa)	CMG (MCM-2-7)	48	345	126	1169
MCM-3 (91 kDa)	CMG (MCM-2-7)	362	493	102	1368
MCM-4 (92 kDa)	CMG (MCM-2-7)	180	425	130	1273
MCM-5 (85 kDa)	CMG (MCM-2-7)	91	373	73	1663
MCM-6 (91 kDa)	CMG (MCM-2-7)	92	356	102	1049
MCM-7 (82 kDa)	CMG (MCM-2-7)	167	444	85	1805
TIM-1 (157 kDa)	TIMELESS-TIPIN	0	201	0	510
TIPN-1 (27 kDa)	TIMELESS-TIPIN	0	18	0	43
CLSP-1 (85 kDa)	CLASPIN	0	13	7	41
SPT-16 (117 kDa)	FACT (SPT16)	0	94	15	246
HMG-3 (78 kDa)	FACT (SSRP1)	0	25	3	82
HMG-4 (79 kDa)	FACT (SSRP1)	0	19	0	60
POLE-1 (245 kDa)	POL epsilon	14	102	53	181
POLE-2 (61 kDa)	POL epsilon	5	16	15	64
LRR-1 (51 kDa)	CUL-2_LRR-1	0	4	0	138

CTF-18 (97 kDa)	CTF18-RFC	13	44	12	45
DSCC-1 (51 kDa)	CTF18-RFC	6	8	6	28
CTF-8 (12 kDa)	CTF18-RFC	0	3	0	3

The samples presented in Figure S2 were analysed by mass spectrometry as described in Materials and Methods. The above table presents spectral counts for the indicated factors in this experiment, corresponding to previously characterised components of the worm replisome.

**Protein**	**Replisome sub-complex**	**Spectral counts (control)**	**Spectral counts (GFP-PSF-1)**	**Spectral counts (GFP-PSF-1) *cdc-45* RNAi**	**Spectral counts (GFP-PSF-1) *npl-4* RNAi**
TNSL-1 (137 kDa)	TONSL	0	0	0	46
TRES-1 (98 kDa)	TRESLIN	0	2	4	61
MUS-101 (134 kDa)	TOPBP1	9	13	13	39
DNSN-1 (66 kDa)	DONSON	7	42	34	205

Data from the same experiment, identifying four additional proteins that co-purify with GFP-PSF-1 under the indicated conditions. The complete dataset is presented in Data S1. Raw data have been deposited to the ProteomeXchange Consortium via the PRIDE partner repository (*77*) with the dataset identifier PXD044240 and 10.6019/PXD044240.

Table S2.
MS analysis of DNSN-1 partners.

Protein	Replisome sub-complex	Spectral counts (control)	Spectral counts (GFP-DNSN-1)	Spectral counts (DNSN-1-GFP)
DNSN-1 +GFP (94 kDa)	DONSON	13	3371	3507
PSF-1 (23 kDa)	CMG (GINS)	18	158	138
PSF-2 (20 kDa)	CMG (GINS)	14	108	84
PSF-3 (22 kDa)	CMG (GINS)	20	131	119
SLD-5 (26 kDa)	CMG (GINS)	7	120	105
CDC-45 (66 kDa)	CMG (CDC-45)	26	186	149
MCM-2 (99 kDa)	CMG (MCM-2-7)	103	599	519
MCM-3 (91 kDa)	CMG (MCM-2-7)	58	408	303
MCM-4 (92 kDa)	CMG (MCM-2-7)	129	684	510
MCM-5 (85 kDa)	CMG (MCM-2-7)	107	575	454
MCM-6 (91 kDa)	CMG (MCM-2-7)	111	485	390
MCM-7 (82 kDa)	CMG (MCM-2-7)	156	889	803
TIM-1 (157 kDa)	TIMELESS-TIPIN	51	219	235
TIPN-1 (27 kDa)	TIMELESS-TIPIN	6	25	19
CLSP-1 (85 kDa)	CLASPIN	25	64	50
POLE-1 (245 kDa)	POL epsilon	48	246	272
POLE-2 (61 kDa)	POL epsilon	17	76	94
POLE-3 (15 kDa)	POL epsilon	6	17	21
POLE-4 (21 kDa)	POL epsilon	2	17	18
LRR-1 (51 kDa)	CUL-2_LRR-1	29	84	83

CTF-18 (97 kDa)	CTF18-RFC	16	71	66
DSCC-1 (51 kDa)	CTF18-RFC	13	51	41
CTF-8 (12 kDa)	CTF18-RFC	0	4	6
TNSL-1 (137 kDa)	TONSL	12	31	29
TRES-1 (98 kDa)	TRESLIN	22	83	110
MUS-101 (134 kDa)	TOPBP1	58	352	404

The data correspond to the samples in Figure S1D. The complete dataset is presented in Data S2. Raw data have been deposited to the ProteomeXchange Consortium via the PRIDE partner repository (*77*) with the dataset identifier PXD044240 and 10.6019/PXD044240.

Table S3.
MS analysis of purified worm replisomes after RNAi depletion of TIM-1, CTF-4 or CLSP-1.

Protein	Replisome sub-complex	Spectral counts (*npl-4* RNAi)	Spectral counts (*npl-4* + *tim-1* RNAi)	Spectral counts (*npl-4* + *ctf-4* RNAi)	Spectral counts (*npl-4* + *clsp-1* RNAi)
TAP-PSF-1 (45 kDa)	CMG (GINS)	1075	1006	1181	1041
PSF-2 (20 kDa)	CMG (GINS)	220	194	254	178
PSF-3 (22 kDa)	CMG (GINS)	417	408	398	341
SLD-5 (26 kDa)	CMG (GINS)	469	443	503	491
CDC-45 (66 kDa)	CMG (CDC-45)	581	668	613	642
MCM-2 (99 kDa)	CMG (MCM-2-7)	1137	1321	1318	1218
MCM-3 (91 kDa)	CMG (MCM-2-7)	990	1146	1132	1071
MCM-4 (92 kDa)	CMG (MCM-2-7)	1137	1232	1321	1133
MCM-5 (85 kDa)	CMG (MCM-2-7)	1206	1265	1315	1191
MCM-6 (91 kDa)	CMG (MCM-2-7)	962	1141	1135	1036
MCM-7 (82 kDa)	CMG (MCM-2-7)	1331	1288	1546	1297
TIM-1 (157 kDa)	TIMELESS-TIPIN	490	12	692	546
TIPN-1 (27 kDa)	TIMELESS-TIPIN	38	0	82	47
CLSP-1 (85 kDa)	CLASPIN	118	49	209	26
CTF-4 (123 kDa)	CTF-4	96	65	3	116
SPT-16 (117 kDa)	FACT (SPT16)	617	487	755	584
HMG-3 (78 kDa)	FACT (SSRP1)	218	132	274	215
HMG-4 (79 kDa)	FACT (SSRP1)	116	36	158	90
POLE-1 (245 kDa)	POL epsilon	99	90	121	124
POLE-2 (61 kDa)	POL epsilon	14	12	12	25

LRR-1 (51 kDa)	CUL-2_LRR-1	97	14	179	114
CTF-18 (97 kDa)	CTF18-RFC	48	20	67	84
DSCC-1 (51 kDa)	CTF18-RFC	20	7	22	30
CTF-8 (12 kDa)	CTF18-RFC	9	0	10	11

TNSL-1 (137 kDa)	TONSL	68	64	80	47
TRES-1 (98 kDa)	TRESLIN	5	10	6	10
MUS-101 (134 kDa)	TOPBP1	27	36	42	28
DNSN-1 (66 kDa)	DONSON	311	501	412	397

TAP-psf-1 worms were treated with the indicated RNAi treatments and TAP-PSF-1 was then isolated from embryo extracts before mass spectrometry analysis. The table presents spectral counts for the indicated factors in this experiment. The complete dataset is presented in Data S3. Raw data have been deposited to the ProteomeXchange Consortium via the PRIDE partner repository (*77*) with the dataset identifier PXD044240 and 10.6019/PXD044240.

Table S4. Cryo-EM data collection and refinement statistics.

	CMG/TIM-1/ TIPN-1/ DNSN-1	CMG/ DNSN-1	DNSN-1 MBR	GINS/ CDC-45 MBR	MCM-2-7 AAA+ MBR	MCM-2-7 NTD MBR	MCM NTD/ TIM-1/ TIPN-1 MBR
	PDB 8OUW EMD-17204	EMD-17198	EMD-17199	EMD-17200	EMD-17201	EMD-17202	EMD-17203
**Data collection and processing**							
Microscope	Titan Krios	Titan Krios	Titan Krios	Titan Krios	Titan Krios	Titan Krios	Titan Krios
Voltage (keV)	300	300	300	300	300	300	300
Camera	K3	K3	K3	K3	K3	K3	K3
Magnification	81,000	81,000	81,000	81,000	81,000	81,000	81,000
Pixel size at detector (Å/pixel)	1.09	1.09	1.09	1.09	1.09	1.09	1.09
Total electron exposure (e-/Å2)	40.1	40.1	40.1	40.1	40.1	40.1	40.1
Exposure rate (e-/pixel/sec)	28.0	28.0	28.0	28.0	28.0	28.0	28.0
Number of frames collected	41	41	41	41	41	41	41
Defocus range (μm)	-1.0 to -2.5	-1.0 to -2.5	-1.0 to -2.5	-1.0 to -2.5	-1.0 to -2.5	-1.0 to -2.5	-1.0 to -2.5
Automation software	EPU	EPU	EPU	EPU	EPU	EPU	EPU
Energy filter slit width (eV)	20	20	20	20	20	20	20
Micrographs collected	10,825	10,825	10,825	10,825	10,825	10,825	10,825
Total extracted particles	4,540,000	4,540,000	4,540,000	4,540,000	4,540,000	4,540,000	4,540,000
Final particle images	33,900	170,000	170,000	170,000	170,000	170,000	922,000
Point-group symmetry	C1	C1	C1	C1	C1	C1	C1
Resolution (global, Å)							
FSC 0.5 (unmasked)	8.13	4.64	7.24	4.48	6.87	7.01	3.77
FSC 0.5 (masked)	4.41	3.77	3.97	3.32	3.64	3.66	3.01
FSC 0.143 (unmasked)	4.62	3.70	4.65	3.59	3.89	3.92	3.15
FSC 0.143 (masked)	3.75	3.25	3.44	2.88	3.17	3.20	2.64
Map sharpening *B* factor ($Å^2$)	-20	-15	-20	-20	-20	-20	-20
**Model composition**							
Non-hydrogen atoms	53,760						
Protein residues	6,564						
Ligands (AMPPNP/Mg^{2+}/Zn^{2+})	4/4/5						
DNA residues	67						
**Model refinement**							
Refinement package (real space)	Phenix 1.19						
Model resolution cutoff (Å)	4						
Model-Map CC (CC_{mask}/CC_{box}/ CC_{peaks}/CC_{volume})	0.64/0.76/ 0.61/0.64						
Model-Map FSC, threshold 0.50 (masked/unmasked, Å)	4.3/4.2						
**B-factors**							
Average B-factors (A^2)							
Protein	84.65						
Ligand	33.51						
Nucleotide	20.00						
**RMSDs**							
Bond lengths (Å)	0.005						
Bond angles (°)	0.881						
**Validation**							
MolProbity score	0.57						
CaBLAM outliers	0.99						
Clashscore	0.19						
Poor rotamers (%)	0.38						
C-beta deviations	0.00						
**Ramachandran plot**							
Favored (%)	98.05						
Outliers (%)	0.00						

MBR: Multi-body refinement

Table S5. Reagents and resources from this study.

REAGENT or RESOURCE	SOURCE	IDENTIFIER
**Antibodies**		
Anti-MCM-2 [antigen 1-222 sheep]<br>Use 1 in 2,000 for immunoblotting	Ref. (*35*)	S750D
Anti-MCM-7 [antigen 1-222 sheep]<br>Use 1 in 3,000 for immunoblotting	Ref. (*35*)	S797D
Anti-CDC-45 [antigen 1-222 sheep]<br>Use 1 in 500 for immunoblotting	Ref. (*35*)	S782D
Anti-PSF-1 [antigen full-length sheep]<br>Use 1 in 500 for immunoblotting	Ref. (*35*)	S789D
Anti-SLD-5 [antigen 1-222 sheep]<br>Use 1 in 1,500 for immunoblotting	Ref. (*59*)	SA419
Anti-CLSP-1 [antigen 1-222 sheep]<br>Use 1 in 3,000 for immunoblotting	Ref. (*59*)	SA426
Anti-CTF-4 [antigen 1-222 sheep]<br>Use 1 in 1,000 for immunoblotting	Ref. (*59*)	SA416
Anti-TIPN-1 [antigen 1-222 sheep]<br>Use 1 in 3,000 for immunoblotting	Ref. (*59*)	SA421
Anti-POLE-1 [antigen 1-222 sheep]<br>Use 1 in 500 for immunoblotting	This study, MRC PPU Reagents & Services	SA573
Anti-CTF-18 [antigen 1-222 sheep]<br>Use 1 in 500for immunoblotting	This study, MRC PPU Reagents & Services	SA576
Anti-DNSN-1 [antigen 1-222 sheep]<br>Use 1 in 1000 for immunoblotting	This study, MRC PPU Reagents & Services	SA577
Anti-MUS-101 [antigen 1-318 sheep]<br>Use 1 in 500 for immunoblotting	This study, MRC PPU Reagents & Services	DA248
anti-sheep IgG HRP [from donkey]<br>Use 1 in 10,000 for immunoblotting	Sigma-Aldrich	A3415
**Bacterial and Virus Strains**		
Escherichia coli: Rosetta™ (DE3) pLysS cells: F^- *ompT*<br>*hsdS*$_B$(r_B^- m_B^-) *gal dcm* (DE3) pLysSRARE (Cam^R)	Novagen	70956
**Chemicals, Peptides, and Recombinant Proteins (unique isoforms unless mentioned otherwise)**		
CMG (including MCM-2 isoform a, MCM-4 isoform a, MCM-6 isoform a and SLD-5 isoform a; other components only have one isoform)	Ref. (*59*)	N/A
TIM-1_TIPN-1	Ref. (*59*)	N/A
DNSN-1	This study	N/A
GINS (including SLD-5 isoform a; other components only have one isoform)	This study	N/A
Dynabeads M-270 Epoxy	ThermoFisher Scientific	14302D
Ni-NTA agarose	Qiagen	30210
Calmodulin sepharose 4B	GE Healthcare	17052901
IgG sepharose 6 Fast Flow	GE Healthcare	17096901
GFP-Trap Agarose	Chromotek	gta
GFP-Trap Magnetic Particles M-270	Chromotek	gtd

RFP-Trap Magnetic Particles M-270	Chromotek	rtdk
Roche cOmplete EDTA-free protease inhibitor cocktail	Roche	000000011873580001
Sigma protease inhibitor cocktail	Sigma-Aldrich	P8215
AEBSF	Sigma-Aldrich	A8456
Pepstatin A	Sigma-Aldrich	P5318
PreScission protease	Axel Knebel, MRC PPU Reagents & Services	DU34905
TEV protease	Axel Knebel, MRC PPU Reagents & Services	DU6811
Ubquitin PrG	Axel Knebel, MRC PPU Reagents & Services	DU49003
AcTEV	ThermoFisher Scientific	12575015
Hoechst 33342	Invitrogen	H1399
Adenosine 5′-(β,γ-imido)triphosphate lithium salt hydrate (AMP-PNP)	Sigma	A2647
Glutaraldehyde	Sigma	G5882
Suberic acid bis(3-sulfo-N-hydroxysuccinimide ester) sodium salt (BS^3)	Sigma	S5799
TWEEN® 20	Sigma	P8341
QUANTIFOIL Copper 400 mesh R2/2 holey carbon TEM grids with 2nm ultrathin continuous carbon support	Electron Microscopy Sciences	Q425CR2-2nm
**Critical Commercial Assays**		
Click-iT™ Plus Alexa Fluor™ 647 Picolyl Azide Toolkit	Invitrogen	C10643
FiberPrep® DNA extraction Kit	Genomics Vision	EXT-001A
CombiCoverslips™	Genomics Vision	COV-002-RUO
FiberComb® Molecular Combing System	Genomics Vision	MCS-001
**Experimental Models: Organisms/Strains**		
C. elegans: Strain N2 Bristol (wild type)		N2
C. elegans: Strain KAL1, *GFP-psf-1*: *psf-1(lab1[gfp::TEV::S-tag::psf-1 + loxP unc-119(+) loxP])*	Ref. (*35*)	KAL1
C. elegans: Strain KAL3, *GFP-psf-1; mCherry-histone H2B:* *psf-1(lab1[gfp::TEV::S-tag::psf-1 + loxP unc-119(+) loxP]); ltIs37[pie-1p::mCherry::his-58 + unc-119(+)]*	Ref. (*35*)	KAL3
C. elegans: Strain KAL17, *TAP-psf-1*: *psf-1(lab2[tap::psf-1 + loxP unc-119(+) loxP])*	Ref. (*59*)	Knu190
C. elegans: Strain KAL213, *GFP-dnsn-1*: *dnsn-1(syb5085 [gfp::dnsn-1])*	This study, from SunyBiotech	syb5085
C. elegans: Strain KAL214, *dnsn-1-GFP*: *dnsn-1(syb5036 [dnsn-1::gfp])*	This study, from SunyBiotech	syb5036
C. elegans: Strain KAL221, *dnsn-1ΔN*: *dnsn-1(syb6461 [dnsn-1 deletion of H5 to R19])*	This study, from SunyBiotech	syb6461

C. elegans: Strain KAL255, *cdc-7Δ*:<br>*cdc-7(syb7234 [1916 bp deletion])*	This study, from SunyBiotech	syb7234
C. elegans: Strain KAL256, *mCherry-dnsn-1*:<br>*dnsn-1(syb6574 [mCherry::dnsn-1])*	This study, from SunyBiotech	syb6574
C. elegans: Strain KAL257, *mCherry-dnsn-1ΔN*:<br>*dnsn-1(syb6840 [mCherry::dnsn-1 deletion of H5 to R19])*	This study, from SunyBiotech	syb6840
C. elegans: Strain KAL259, *mcm-10Δ*:<br>*mcm-10(syb2118 [8226 bp deletion])*	This study, from SunyBiotech	syb2118
C. elegans: Strain KAL265*, GFP-histone H2B; mCherry-cdc-45:*<br>*gtIs2512[Ppie-1::his-11::GFP unc-119+]; cdc-45(syb6509 [mCherry::cdc-45]);*	This study	KAL265
C. elegans: Strain KAL266*, GFP-psf-1; mCherry-cdc-45*:<br>*psf-1(lab1[gfp::TEV::S-tag::psf-1 + loxP unc-119(+) loxP]); cdc-45(syb6509 [mCherry::cdc-45])*	This study	KAL266
C. elegans: Strain KAL267*, GFP-dnsn-1; mCherry-histone H2B*:<br>*dnsn-1(syb5085 [gfp::dnsn-1]); ltIs37[pie-1p::mCherry::his-58 + unc-119(+)]*	This study	KAL267
C. elegans: Strain KAL268*, GFP-dnsn-1; mCherry-cdc-45*:<br>*dnsn-1(syb5085 [gfp::dnsn-1]); cdc-45(syb6509 [mCherry::cdc-45])*	This study	KAL268
C. elegans: Strain KAL269*, GFP-dnsn-1; mCherry-psf-1*:<br>*dnsn-1(syb5085 [gfp::dnsn-1]); psf-1(syb6425 [mCherry::psf-1])*	This study	KAL269
C. elegans: Strain KAL270*, sld-2-GFP; mCherry-histone H2B*:<br>*sld-2(syb6453 [sld-2::GFP]); ltIs37[pie-1p::mCherry::his-58 + unc-119(+)]*	This study	KAL270
C. elegans: Strain KAL 271*, sld-2-GFP; mCherry-cdc-45*:<br>*sld-2(syb6453 [sld-2::GFP]); cdc-45(syb6509 [mCherry::cdc-45])*	This study	KAL271
C. elegans: Strain KAL272*, sld-2-GFP; mCherry-psf-1*:<br>*sld-2(syb6453 [sld-2::GFP]); psf-1(syb6425 [mCherry::psf-1])*	This study	KAL272
C. elegans: Strain KAL274*, tres-1-GFP; mCherry-cdc-45*:<br>*tres-1(syb6420 [tres-1::GFP]); cdc-45(syb6509 [mCherry::cdc-45])*	This study	KAL274
C. elegans: Strain KAL276*, GFP-mus-101; mCherry-histone H2B*:<br>*mus-101(syb6412[GFP::mus-101]); ltIs37[pie-1p::mCherry::his-58 + unc-119(+)]*	This study	KAL276
C. elegans: Strain KAL277*, GFP-mus-101; mCherry-cdc-45*:<br>*mus-101(syb6412[GFP::mus-101]); cdc-45(syb6509 [mCherry::cdc-45])*	This study	KAL277
C. elegans: Strain KAL279, *GFP-sld-5; mCherry-histone H2B:*<br>*sld-5(syb6564[GFP::sld-5]); ltIs37[pie-1p::mCherry::his-58 + unc-119(+)]*	This study	KAL279
C. elegans: Strain KAL280*, mCherry-CDC-45*:<br>*cdc-45(syb6509 [mCherry::cdc-45])*	This study, from SunyBiotech	syb6509
C. elegans: Strain KAL281*, mCherry-PSF-1*:<br>*psf-1(syb6425 [mCherry::psf-1])*	This study, from SunyBiotech	syb6425

C. elegans: Strain KAL282*, sld-2-GFP*: *sld-2(syb6453 [sld-2::GFP])*	This study, from SunyBiotech	syb6453
C. elegans: Strain KAL283*, tres-1-GFP*: *tres-1(syb6420 [tres-1::GFP])*	This study, from SunyBiotech	syb6420
C. elegans: Strain KAL284*, GFP-mus-101*: *mus-101(syb6412 [GFP::mus-101])*	This study, from SunyBiotech	syb6412
C. elegans: Strain KAL285*, GFP-sld-5*: *sld-5(syb6564 [GFP::sld-5])*	This study, from SunyBiotech	syb6564
C. elegans: Strain KAL303*, mCherry-dnsn-1-3A*: *dnsn-1c(syb7840[mCherry::dnsn-1 W425A I429A V431A]) II / +*	This study, from SunyBiotech	syb7840
S. cerevisiae: Strain *yJF1*: *MAT***a** *ade2-1 ura3-1 his3-11,15 trp1-1 leu2-3,112 can1-100 bar1Δ::hphNT pep4Δ::kanMX*	Ref. (*80*)	N/A
S. cerevisiae: Strain *YSS3*: *MAT***a** *ade2-1 ura3-1 his3-11,15 trp1-1 leu2-3,112 can1-100 pep4Δ::ADE2*	Ref. (*59*)	N/A
S. cerevisiae: Strain *YSS4*: *MATα ade2-1 ura3-1 his3-11,15 trp1-1 leu2-3,112 can1-100 pep4Δ::ADE2*	Ref. (*59*)	N/A
S. cerevisiae: Strain *yYX1*(TIM-1_TIPN-1 purification): *MAT***a** *ade2-1 ura3-1 his3-11,15 trp1-1 leu2-3,112 LEU2::pRS305-CBP-PreScissionScission-tipn-1-GAL1,10-tim-1 can1-100 pep4Δ::ADE2*	Ref. (*59*)	N/A
S. cerevisiae: Strain *yYX36*(GINS and CDC-45 expression): *MAT***a** *ade2-1 ura3-1 URA3::pRS306-TAP-psf-1-GAL1,10-sld-5 his3-11,15 HIS3::pRS303-GAL1,10-cdc-45 trp1-1 leu2-3,112 LEU2::pRS305-psf-3-GAL1,10-psf-2 can1-100 pep4Δ::ADE2*	Ref. (*59*)	N/A
S. cerevisiae: Strain *yYX37*(CMG purification): *MATα/ MAT***a** *ade2-1 / ade2-1 ura3-1 URA3::pRS306-mcm-2-GAL1,10-mcm-3 / ura3-1 URA3::pRS306-TAP-psf-1-GAL1,10-sld-5 his3-11,15 / his3-11,15 HIS3::pRS303-GAL1,10-cdc-45 trp1-1 TRP1::pRS304-mcm-6-GAL1,10-mcm-7 / trp1-1 leu2-3,112 LEU2::pRS305-mcm-4-GAL1,10-mcm-5 / leu2-3,112 LEU2::pRS305-psf-3-GAL1,10-psf-2 can1-100 / can1-100 pep4Δ::ADE2 / pep4Δ::ADE2*	Ref. (*59*)	N/A
S. cerevisiae: Strain *PJ69-4A*: *MATa, trp1-901, leu2-3,112, ura3-52, his3200, gal4Δ, gal80Δ, GAL2-ADE2, LYS2::GAL1-ADE2, LYS2::GAL1-HIS3, met2::GAL7-lacZ*	Ref. (*81*)	N/A
**Oligonucleotides**		
RNAi of *dnsn-1* forward primer for construction of wRp-343: **9647**: cgactagtGTTGGAACAATGGCAAGAGTGCTACGCGGAG	This study	N/A

RNAi of *dnsn-1* reverse primer for construction of wRp-343: **9648**: cgtctagaTCAATTCTTAGTCCATTTCACACAATTCTCAG	This study	N/A
RNAi of *gfp* forward primer for construction of wRp-346: **9704**: gACTAGTAGTAAAGGAGAAGAACTTTTCACTGGAGTTG	This study	N/A
RNAi of *gfp* reverse primer for construction of wRp-346: **9705**: gcgTCTAGATTTGTATAGTTCATCCATGCCATGTGTAATC	This study	N/A
RNAi of *sld-2* forward primer for construction of wRp-354: **9948**: cgcgACTAGTATGGAAGAGTGGAAAACGTCTTTC	This study	N/A
RNAi of *sld-2* reverse primer for construction of wRp-354: **9949**: cgcgTCTAGATCAGTACTTCTTCTTGAACATTTG	This study	N/A
RNAi of *tres-1* forward primer for construction of wRp-352: **9950**: cgcgACTAGTGTCAGATTCAGGTGCTCCTAAC	This study	N/A
RNAi of *tres-1* reverse primer for construction of wRp-352: **9951**: cgcgTCTAGATGTCCATCTGATGTATCCACTG	This study	N/A
RNAi of *mus-101* forward primer for construction of wRp-353: **9952**: cgcgACTAGTGATGTAGCTATCTTATCAGTG	This study	N/A
RNAi of *mus-101* reverse primer for construction of wRp-353: **9953**: cgcgTCTAGATGGACACAATACGTGTGATATG	This study	N/A
RNAi of *ctf-18* forward primer for construction of wRp-312: **7085**: gactagtACTGCATTCTGGGTTTGCCGACG	This study	N/A
RNAi of *ctf-18* reverse primer for construction of wRp-312: **7086**: gactagtACCAGCCGGGCCAGAAAGAAGCA	This study	N/A
PCR check primer b of *mcm-10Δ*: **9958**: aagttttcaaaatttttgttcaaaaattac	This study	N/A
PCR check primer b of *mcm-10Δ*: **9959**: tacttttgtctgttcttttgtcaaaatttg	This study	N/A
PCR check primer b of *mcm-10Δ*: **9960**: ttaaaatttgaagcgaatttttgttattgc	This study	N/A
PCR check primer a of *dnsn-1ΔN*: **9890**: cagtctccctttgcgcgcaagca	This study	N/A
PCR check primer b of *dnsn-1ΔN*: **9891**: aagttcatttctctacTTTAAAC	This study	N/A
PCR check primer a of *cdc-7Δ*: **10011**: catgtggcaatgcgcgtaagcttcgccgtaac	This study	N/A
PCR check primer b of *cdc-7Δ*: **10012**: ttaaaataaaattttgaaaaaatggattc	This study	N/A

dnsn-1c FL forward primer for construction of pYXC106: **8378**: gaacagattggtggcATGTCCGACGAGCACTATAATCCT	This study	N/A
dnsn-1c FL reverse primer for construction of pYXC106: **8379**: gtgcggccgcttattaATTCTTAGTCCATTTCACACAATTCTCA	This study	N/A
dnsn-1c 1-20Δ forward primer for construction of pYXC112: **9706**: GATATTATTGAGGCTCACAGAGAACAGATTGGTGGCAGCTGCCGAAACATTCTCAACGCTGTGCCTCCAC	This study	N/A
dnsn-1c 1-57Δ forward primer for construction of pYXC110: **9702**: GATATTATTGAGGCTCACAGAGAACAGATTGGTGGCGTCTCTTCCCCGCAAAAGAAGCGACAAAAAC	This study	N/A
mus-101 1-427 forward primer for construction of pKL3652 and pKL3654: **10056**: gtagctGGTCTCgaattcATGGAGGCACCTCCGGCCCCGAAGAAGGC	This study	N/A
mus-101 1-427 reverse primer for construction of pKL3652: **10058**: gcatcaGGTCTCcccgggTCATGTTTGAGTAGTTGGATATCGAATCAG	This study	N/A
mus-101 1-543 reverse primer for construction of pKL3654: **10059**: atcgcaGGTCTCcccgggTCAAGTCCATTGATAACCTTCCACTG	This study	N/A
*5' oligo to make annealed substrate with 46bp double-strand DNA and 39nt of single-strand DNA (3' flap) TAGAGTAGGAAGTGATGGTAAGTGATTAGAGAATTGGAGAGTGTGTTTTTTTTTTTTTTTTTTTTTTTTTTTTTTTTT*T*T*T*T*T [* denotes phosphorothioate bonds]*	Ref. (*82*)	N/A
3' oligo to make annealed substrate with 46bp double-strand DNA and 39nt of single-strand DNA (3' flap) ACACACTCTCCAATTCTCTAATCACTTACCATCACTTCCTACTCTA	Ref. (*82*)	N/A
*Leading-strand template oligo used to make annealed substrate for cryo-EM sample preparation [Cy3]TAGAGTAGGAAGTGA[iBiodT]GGTAAGTGATTAGAGAATTGGAGAGTGTGTTTTTTTTTTTTTTTTTTTTTTTTTTTTTTTTT*T*T*T*T*T [* denotes phosphorothioate bonds]*	Ref. (*50*)	N/A

Lagging-strand template oligo used to make annealed substrate for cryo-EM sample preparation GGCAGGCAGGCAGGCACACACTCTCCAATTCTC TAATCACTTACCA[iBiodT]CACTTCCTACTCTA	Ref. (*50*)	N/A
**Recombinant DNA**		
pYXC1 [expresses TIM-1 and CBP-PreScission-TIPN-1, for TIM-1_TIPN-1 purification]	Ref. (*59*)	DU70332
pYXC14 [expresses MCM-2a and MCM-3, for CMG purification]	Ref. (*59*)	DU70334
pYXC15 [expresses MCM-4a and MCM-5, for CMG purification]	Ref. (*59*)	DU70335
pYXC17 [expresses CDC-45, for CMG purification]	Ref. (*59*)	DU70336
pYXC19 [expresses PSF-3 and PSF-2, for CMG purification]	Ref. (*59*)	DU70337
pYXC28 [expresses MCM-6a and MCM-7, for CMG purification]	Ref. (*59*)	DU70338
pYXC29 [expresses TAP-PSF-1 and SLD-5a, for CMG purification]	Ref. (*59*)	DU70339
pYXC106 [expresses 14HIS-SUMO-DNSN-1c, for DNSN-1c purification]	This study	DU75873
pYXC110 [expresses 14HIS-SUMO-*dnsn-1c 1-57Δ*, for DNSN-1c 1-57Δ purification]	This study	DU75874
pYXC112 [expresses 14HIS-SUMO-*dnsn-1c 1-20Δ*, for DNSN-1c 1-20Δ purification]	This study	DU75875
pFGET19-Ulp1(403-621)	Addgene	64697
L4440 [RNAi control plasmid]	Ref. (*35*)	DU70356
wRp-316 [RNAi of *npl-4 ctf-4*]	Ref. (*59*)	DU70392
wRp-317 [RNAi of *npl-4 clsp-1*]	Ref. (*59*)	DU70393
wRp-318 [RNAi of *npl-4 tim-1*]	Ref. (*59*)	DU70394
wRp-336 [RNAi of *npl-4*]	Ref. (*35*)	DU70399
wRp-343 [RNAi of *dnsn-1*]	This study	DU75876
wRp-346 [RNAi of *gfp*]	This study	DU75877
wRp-347 [RNAi of *npl-4 gfp*]	This study	DU75878
wRp-348 [RNAi of *atl-1*]	This study	DU75879
wRp-352 [RNAi of *tres-1*]	This study	DU75880
wRp-353 [RNAi of *mus-101*]	This study	DU75881
wRp-354 [RNAi of *sld-2*]	This study	DU75882
wRp-366 [RNAi of *cdc-45*]	This study	DU75883
wRp-367 [RNAi of *npl-4 cdc-45*]	This study	DU75884
pGADT7 (Gal4 activation domain vector)	Clontech (Takara)	630442
pGBKT7 (Gal4 DNA-binding domain vector)	Clontech (Takara)	630443
pKL2653 [pGADT7-*dnsn-1c*]	This study	DU75872
pKL3652 [pGBKT7-*mus-101 1-427*]	This study	DU75871
pKL3654 [pGBKT7-*mus-101 1-543*]	This study	DU75870

Software and Algorithms		
EPU (v2.0)	ThermoFisher Scientific (FEI)	https://www.fei.com/software/epuautomated-single-particles-softwarefor-life-sciences/
COOT (v0.9.5)	Paul Emsley (Medical Research Council Laboratory of Molecular Biology)	https://www2.mrc-lmb.cam.ac.uk/personal/pemsley/coot/
ISOLDE (v1.0.1)	Tristan Croll (Cambridge Institute for Medical Research)	https://isolde.cimr.cam.ac.uk/
Chimera (UCSF) (v1.8.1)	UCSF Resource for Biocomputing, Visualization, and Informatics	https://www.cgl.ucsf.edu/chimera/
ChimeraX (UCSF) (v1.0)	UCSF Resource for Biocomputing, Visualization, and Informatics	https://www.cgl.ucsf.edu/chimerax/
AlphaFold-Multimer (Colabfold v1.2.0)	DeepMind	https://colab.research.google.com/github/deepmind/alphafold/blob/main/notebooks/AlphaFold.ipynb#scrollTo=woIxeCPygt7K
RELION (v4.0)	Sjors Scheres (Medical Research Council Laboratory of Molecular Biology)	https://www3.mrc-lmb.cam.ac.uk/ relion/
MotionCor2 (v2.0)	University of California San Francisco (UCSF) EM Core	https://emcore.ucsf.edu/ucsfmotioncor2
CtfFind (v4.1)	The Grigorieff Lab (UMass Chan medical school)	https://grigoriefflab.umassmed.edu/ctffind4
Gautomatch (v0.56)	Kai Zhang (Medical Research Council Laboratory of Molecular Biology)	https://www.mrc-lmb.cam.ac.uk/kzhang/Gautomatch/
Xmipp (v3.0.1)	Centro Nacional de Biotecnologia (CNB) Instruct Image Processing Centre (I2PC)	http://xmipp.i2pc.es/
EMAN (v1.9)	Baylor College of Medicine	https://cryoem.bcm.edu/downloads/view_eman1_versions

Phenix (v1.19)	Cambridge University; Duke University; Lawrence Berkeley National Laboratory; Los Alamos National Laboratory	https://www.phenix-online.org/
MolProbity	Duke Univeristy	http://molprobity.biochem.duke.edu/
Prism (v9.31)	GraphPad	https://www.graphpad.com/scientificsoftware/prism/
FIJI	National Institute of Health	https://imagej.nih.gov/ij/
ImageJ	National Institute of Health	https://imagej.nih.gov/ij/
Other		

Data S1. (separate file)
Full mass spectrometry dataset for the experiment summarized in Table S1.
Raw data have been deposited to the ProteomeXchange Consortium via the PRIDE partner repository (*77*) with the dataset identifier PXD044240 and 10.6019/PXD044240.

Data S2. (separate file)
Full mass spectrometry dataset for the experiment summarized in Table S2.
Raw data have been deposited to the ProteomeXchange Consortium via the PRIDE partner repository (*77*) with the dataset identifier PXD044240 and 10.6019/PXD044240.

Data S3. (separate file)
Full mass spectrometry dataset for the experiment summarized in Table S3.
Raw data have been deposited to the ProteomeXchange Consortium via the PRIDE partner repository (*77*) with the dataset identifier PXD044240 and 10.6019/PXD044240.
